# Annexin A2 Causes Motor Incoordination via Muscle–Cerebellum Axis in Sarcopenia

**DOI:** 10.1002/jcsm.70203

**Published:** 2026-01-26

**Authors:** Xin Jiao, Zengguang Wang, Hanwen Chang, Yongjin Li, Binbin Wang, Xinfa Shao, Yuxin Zhang, Yixuan Lin, Xinlin Jia, Xianhao Zhou, Wentao Li, Dinghao Luo, Tanjun Deng, Xingzuan Lin, Chen Xu, Yaokai Gan, Dongyun Gu

**Affiliations:** ^1^ Shanghai Key Laboratory of Orthopedic Implant, Department of Orthopedic Surgery, Shanghai Ninth People's Hospital Shanghai Jiao Tong University School of Medicine Shanghai China; ^2^ Guangdong Key Laboratory for Biomedical Measurements and Ultrasound Imaging, National‐Regional Key Technology Engineering Laboratory for Medical Ultrasound, School of Biomedical Engineering Shenzhen University Medical School Shenzhen China; ^3^ Engineering Research Center of Digital Medicine and Clinical Translation, School of Biomedical Engineering & Med‐X Research Institute, Ministry of Education Shanghai Jiao Tong University Shanghai China; ^4^ Neuroscience and Neuroengineering Center, Med‐X Research Institute and School of Biomedical Engineering Shanghai Jiao Tong University Shanghai China; ^5^ Department of Oral Surgery, Shanghai Ninth People's Hospital, Shanghai Jiao Tong University School of Medicine; College of Stomatology, Shanghai Jiao Tong University; National Center for Stomatology; National Clinical Research Center for Oral Diseases; Shanghai Key Laboratory of Stomatology; Shanghai Research Institute of Stomatology; Research Unit of Oral and Maxillofacial Regenerative Medicine Chinese Academy of Medical Sciences Shanghai China; ^6^ Department of Sports Medicine, Institute of Sports Medicine of Peking University, Beijing Key Laboratory of Sports Injuries Peking University Third Hospital Beijing China; ^7^ Department of Neurosurgery, Shanghai Ninth People's Hospital Shanghai Jiao Tong University School of Medicine Shanghai China

**Keywords:** ANXA2, isoliquiritigenin, motor coordination, muscle–cerebellum axis, Purkinje cells, sarcopenia

## Abstract

**Background:**

Sarcopenia is a prevalent age‐related disorder characterized by progressive muscle atrophy. Impaired balance is one of its most critical clinical consequences, often leading to falling and even bone fractures. As the cerebellum plays a central role in regulating motor coordination, elucidating the molecular mechanisms underlying imbalance in sarcopenia, particularly those mediated by the muscle–cerebellum axis, remains an important yet unresolved question.

**Methods:**

4D label‐free proteomics was employed to identify the key secretory protein mediating the interaction between muscles and cerebellums in young and aged mice. Annexin A2 (ANXA2), the candidate protein, was subsequently overexpressed using adeno‐associated virus (AAV), and its effects on both muscle and cerebellum were systematically examined. RNA‐sequencing was conducted to elucidate the molecular mechanisms underlying ANXA2 function in muscle, while stereotactic injection was performed to investigate its impact on cerebellum and related mechanisms. Finally, we evaluated the therapeutic potential of isoliquiritigenin, an inhibitor of ANXA2, in improving motor coordination and muscle function in aged mice.

**Results:**

Aged mice showed obviously impaired motor coordination in the accelerated rotarod (AR) test (*p* < 0.01) and reduced strength performance in the grip strength assay (*p* < 0.05) compared to young mice. Proteomic analysis identified ANXA2 as a secretory protein predominantly produced by aged skeletal muscles (*p* < 0.05 in tibialis anterior, gastrocnemius muscle and quadriceps femoris) but not by other aged organs such as heart, liver, kidney, spleen and lung (all *p* > 0.05). Functionally, ANXA2 exacerbated muscle atrophy by upregulating atrophy‐related markers MuRF‐1 and Atrogin‐1 (both *p* < 0.05) and reducing the myotube diameter via regulation of Neuraminidase 2 (Neu2) (*p* < 0.05). Moreover, ANXA2 was transported into the cerebellum through the blood stream and targeted type 2 cannabinoid receptors (CB2R) in cerebellar Purkinje cells (PCs) of lobule IV/V, thereby contributing to motor incoordination as evidenced by impaired performance in AR tests (*p* < 0.05). Importantly, isoliquiritigenin, an extract from licorice, effectively inhibited ANXA2 expression in muscle (*p* < 0.05), alleviated muscle atrophy (*p* < 0.05) and motor incoordination (*p* < 0.05), while showing no adverse effects on anxiety‐like behaviours associated with CB2R (*p* > 0.05).

**Conclusions:**

ANXA2 is a key mediator of the muscle–cerebellum axis in sarcopenia, contributing to muscle atrophy by downregulating Neu2 and motor incoordination by targeting CB2R. Isoliquiritigenin was identified as an effective compound targeting ANXA2 to improve motor deficits. These findings highlight ANXA2 as a potential therapeutic target and suggest isoliquiritigenin as a promising strategy for alleviating motor incoordination associated with sarcopenia.

## 1 Introduction

Motor incoordination or imbalance is widely reported in patients with sarcopenia [1]. Clinically, it manifests as postural dysfunction [2] and an increased risk of falls [3], as evidenced by impaired center‐of‐pressure (CoP) measures [1], reduced one‐leg stance time [4] and prolonged Timed Up and Go performance [4]. The motor deficits observed in sarcopenia are likely associated with multiple factors, including muscle/fat mass [5], muscle strength [6] and parathyroid hormone levels [7]. Mechanically, peroxisome proliferator‐activated receptor γ coactivator 1α (PGC‐1α) has been widely implicated in modulating motor coordination. Xiong et al. demonstrated that Arctigenin derivative A‐1 ameliorated motor dysfunction in SOD1G93A transgenic mice via the AMP‐activated protein kinase (AMPK)/silent information regulator 1 (SIRT1)/PGC‐1α pathway [8]. More recently, PGC‐1α has been reported to influence motor coordination in sarcopenia, as muscle‐specific PGC‐1α‐knockout mice exhibited impaired performance in both rotarod and balance beam tests [9]. Despite these advances, the underlying mechanisms of motor incoordination in sarcopenia, particularly at the molecular level, remain largely unclear.

Skeletal muscle is increasingly recognized as a vital endocrine organ [10]. Muscle‐derived signalling molecules, known as myokines, play crucial roles in regulating metabolism, inflammation and diverse physiological processes [11]. Crosstalk between muscle and brain has also been widely reported [12], with particular attention to the role of myokines such as irisin [13]. Irisin is secreted by skeletal muscle following exercise and transported into the brain, where it enhances cognitive function [14]. In parallel, the cerebellum is well established as a critical structure for motor coordination [15]. Dysfunction of Purkinje cells, for instance due to genetic alterations, can result in motor incoordination or even ataxia [16]. However, whether a muscle–cerebellum axis contributes to motor incoordination in sarcopenia has not yet been investigated.

In this study, we carried out a series of cellular and animal experiments, including behavioural tests, 4D label‐free proteomics, Western Blot, quantitative real‐time polymerase chain reaction (qRT‐PCR), immunofluorescence and c‐Fos immunohistochemistry to reveal the mechanism by which the muscle–cerebellum axis contributes to motor incoordination in sarcopenia.

## 2 Methods

### 2.1 Animals

All the animal studies were approved by the Ethics Committee of Shanghai Jiao Tong University (2023032) and the Ethics Committee of Wetry Biotechnology (Shanghai) Co. Ltd (WTP20231211001). Male C57BL/6J mice (3 or 20 months old) were purchased from Vital River Laboratory Animal Technology Co. All mice were bred and kept under specific‐pathogen‐free (SPF) conditions with a 12‐hour dark–light cycle at 22°C and 55%–60% humidity and provided with sufficient water and food.

### 2.2 Cell Culture

C2C12 myoblasts were kindly provided by Stem Cell Bank, Chinese Academy of Sciences. C2C12 cells were cultured in Dulbecco's modified Eagle's medium (DMEM, HyClone) with 10% fetal bovine serum (Avantor, USA) and 1% penicillin/streptomycin (New Cell & Molecular Biotech, NCM Biotech, China) and incubated in a humidified condition with 95% air and 5% CO_2_ at 37°C. The medium was replaced with fresh medium every 2–3 days and cells were passaged when they reached 80% confluency.

### 2.3 4D‐Label Free Quantitative Proteomics

Proteomics was conducted to explore the potential mechanism by which muscle influenced cerebellum. The tibialis anterior (TA) muscles and cerebellums were harvested and frozen with liquid nitrogen immediately. The differentially expressed proteins in young and aged mice group were analysed by 4D label‐free quantitative proteomics (Shanghai OE Biotech Co. Ltd, China).

### 2.4 Adenovirus and Adeno‐Associated Virus (AAV) Construction

To overexpress Annexin A2 (ANXA2) in C2C12 cells, pAdEasy‐EF1‐mANXA2‐3flag‐CMV‐EGFP (overexpression, shown as OE or ANXA2‐OE) and pAdEasy‐EF1‐MCS‐3flag‐CMV‐EGFP (control, shown as NC or ANXA2‐NC) were constructed by Hanbio Biotechnology Co. Ltd. (Shanghai, China). To overexpress ANXA2 in muscles, AAV8‐tMCK‐mANXA2–3flag‐T2A‐EGFP‐WPRE (overexpression, shown as AAV‐OE) and AAV8‐tMCK‐Scramble‐3flag‐T2A‐EGFP‐WPRE (control, shown as AAV‐NC) were constructed by Genomeditech (Shanghai) Co. Ltd. To knock down the ANXA2 levels in muscles, AAV8‐shANXA2‐T2A‐EGFP‐WPRE (knockdown, shown as shANXA2) and AAV8‐scramble‐T2A‐EGFP‐WPRE (control, shown as shNC) were obtained from Genomeditech (Shanghai) Co. Ltd. To knock down the CB2R in cerebellums, AAV9‐shCB2R‐EGFP (knockdown, shown as AAV‐shCB2R) and AAV9‐scramble‐EGFP (control, shown as AAV‐shNC) were purchased from Genomeditech (Shanghai) Co. Ltd. The sequences of shANXA2 and shCB2R are listed in Table S3. For neuronal excitation, AAV9‐L7‐6‐hM3D(Gq)‐mCitrine and AAV9‐L7‐6‐mCitrine were provided by Genomeditech (Shanghai) Co. Ltd.

### 2.5 Adenovirus and AAV Infection

For C2C12 cell infection, when cell confluency reached 30%–50%, the adenovirus was added in the fresh medium at 1/2 volume. The medium was replenished after 4 h and changed at the second day. The myogenic induction medium was altered at 80% confluency. For muscle infection, 10^11^ VG was injected into bilateral quadriceps femoris (Q), gastrocnemius (G) muscle and tibialis anterior (TA) muscle by micro injection pump (Harvard Apparatus, USA). The needle was left in the tissue for 30 s before being withdrawn. For cerebellum 4/5 lobule (Cb4/5) infection, the micro syringe (33G, 20 mm, Hamilton, USA) was used to inject 300 nL AAV solution into Cb4/5. The methods in detail were described in the supplementary materials.

### 2.6 siRNA Transfection

The siRNA of ANXA (shown as siANXA2) and Neu2 (shown as siNeu2) was provided by Hanbio Biotechnology Co. Ltd. and stored in −20°C at the concentration of 20 μM in ddH_2_O. The sequences of siRNA are listed in Table S4. siRNA transfection was conducted when the cell confluency reached around 50% using Lipofectamine RNAiMAX (Thermo Scientific, USA), according to the instructions of the manufacturers. In brief, the siRNA and transfection reagents were diluted in Opti‐MEM (Thermo Scientific, USA). Then, the solution was mixed and incubated at room temperature for 5 min. Next, the mixture was added to the fresh cell medium. On the second day, the medium was changed for subsequent treatment.

### 2.7 Plasmid Preparation and Transfection

The plasmids pcDNA3.1‐EF1a‐mcs‐3flag‐CMV‐EGFP (shown as Neu2‐NC) and pcDNA3.1‐EF1a‐mNeu2‐3flag‐CMV‐EGFP (shown as Neu2‐OE) were offered by Hanbio Biotechnology Co. For plasmid transfection with adenovirus infection, we performed plasmid transfection with Lipofectamine 3000 (Thermo Scientific, USA) first. The DNA together with P3000 and transfection reagent in Opti‐MEM was prepared, respectively, and mixed to incubate for 10–15 min at room temperature. Twelve hours later, adenovirus infection was performed as abovementioned. On the second day, the subsequent treatment was conducted.

### 2.8 Statistical Analysis

Statistical analyses were performed using Prism 9 (GraphPad Software) and the data are presented as mean ± SEM. An unpaired two‐tailed Student's *t*‐test was used to investigate significance between two groups. For multiple groups, an ordinary one‐way ANOVA was used, followed by Tukey's test. Differences were considered significant when *p* < 0.05.

## 3 Results

### 3.1 Aged Mice Shows Impaired Motor Coordination and Muscle Strength

**FIGURE 1:**
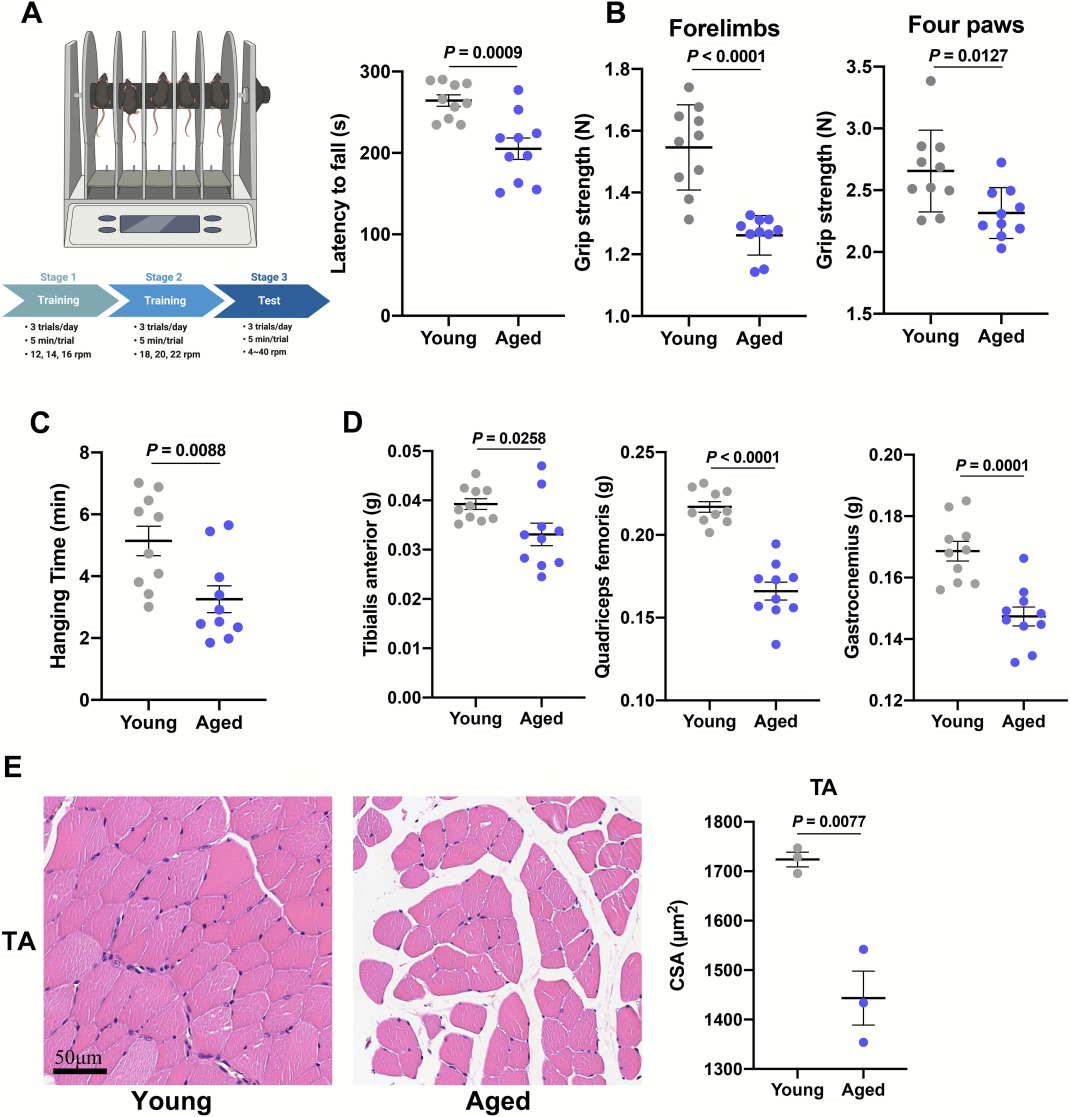
Motor coordination performance and muscle function of aged mice. (A) Schematic diagram on the timeline of accelerated rotarod (AR) procedure (left) and AR latency to fall of young and aged mice (right) (*n* = 10). (B) Grip strength of forelimbs and four paws of young and aged mice (*n* = 10). (C) Hanging time of young and aged mice in hanging grid tests (*n* = 10). (D) The masses of tibialis anterior, quadriceps femoris, gastrocnemius muscles of young and aged mice (*n* = 10). (E) Representative images (left) and cross sectional area (CSA) (right) of TA in young and aged mice (*n* = 3). Values are represented as means ± SEM. Exact *p* values are shown.

Aged mice showed motor incoordination, as reflected by reduced latency to fall in accelerated rotarod (AR) tests (Figure 1A). Balance beam test is a widely used assessment for evaluating balance and motor coordination [17]. In our study, aged mice demonstrated longer running time on both the 12‐ and 6 mm‐wide balance beams (Figure S1A). The pole test is used to gauge the combination of motor coordination and strength [18]. Compared with young mice, aged mice displayed longer return time and total time (Figure S1B). Gait analysis was employed to measure gait coordination. As shown in Figure S1C, aged mice manifested lower average speed, body speed and swing speed. Meanwhile, aged mice also showed fewer cadences, longer stand time and swing time (Figure S1D). In addition, single‐limb and diagonal‐limb support phases became shorter while three‐limb and four‐limb support phases got longer in the gait cycle, suggesting gait incoordination in aged mice (Figure S1E). Moreover, the grip strength of forelimbs and four paws declined in aged mice compared with young mice (Figure 1B). Simultaneously, hanging grid test was performed to assess the muscle strength [19]. Like the grip strength, the hanging time of aged mice also decreased (Figure 1C). The masses of TA, Q, G, extensor digitorum longus, soleus (SOL) and plantaris muscles were reduced in aged mice (Figures 1D and S1F). Consistent with muscle mass, the cross sectional areas (CSAs) of TA and G both fell in aged mice (Figures 1E and S1G). These results implied that aged mice showed impaired motor coordination and muscle strength and mass.

### 3.2 ANXA2 Increases in Both Muscles and Cerebellums of Aged Mice

**FIGURE 2:**
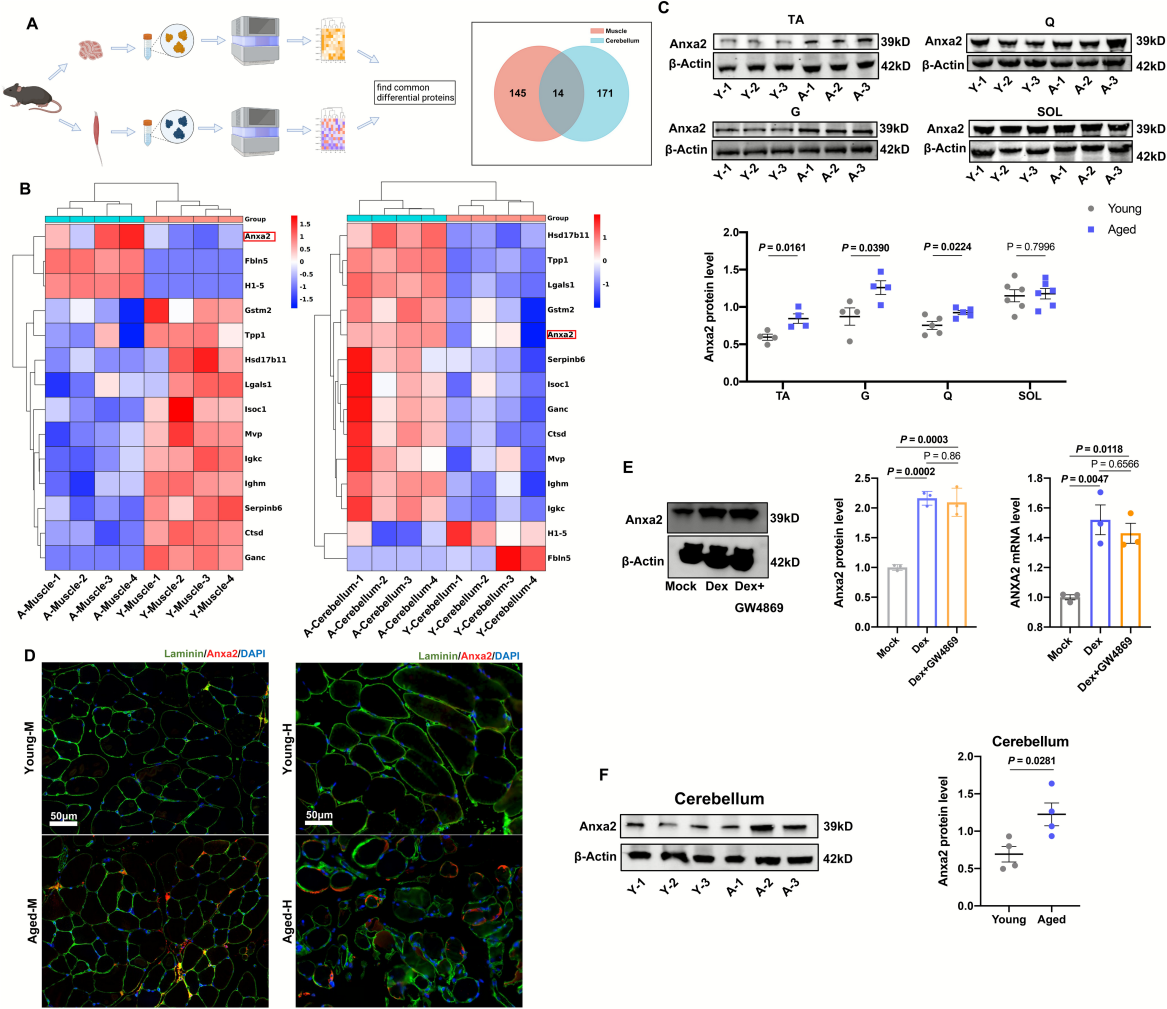
ANXA2 increased in both muscles and cerebellums of aged mice. (A) Schematic diagram (left) on the process of common differential protein identification and Venn diagram (right) of differential expressed protein from muscles and cerebellums in young and aged mice. (B) Expression of 14 common differential proteins in muscles and cerebellums. ‘A’ represents aged mice, ‘Y’ represents young mice. (C) The protein levels of ANXA2 in tibialis anterior (TA), gastrocnemius (G), quadriceps femoris (Q), soleus (SOL) and statistical results in young and aged mice by Western Blot (*n* = 4). (D) The expression of ANXA2 in tibialis anterior in young and aged mice (Young‐M, Aged‐M) and humans (Young‐H, Aged‐H) by immunofluorescence. (E) The protein and RNA levels of ANXA2 in C2C12 myotubes in Mock, Dexamethasone (Dex), Dex + GW4869 groups and statistical results by Western Blot and qRT‐PCR (*n* = 3). (F) The protein levels of ANXA2 in cerebellums from young and aged mice and statistical results by Western Blot (*n* = 4). Values are represented as means ± SEM. Exact *p* values are shown.

Next, we collected TA and cerebellums and employed 4D label‐free proteomics to detect common differential proteins (Figure 2A). The differential proteins of muscle were expressed in Figure S2A. In total, 70 proteins were upregulated and 89 were downregulated. The differential proteins of cerebellum were expressed in Figure S2B. It was found that 132 proteins were upregulated and 53 were downregulated. Among these differential proteins, fourteen were shared (Figure 2A). Further analysis found ANXA2 expression ascended in both muscles and cerebellums in aged mice (Figure 2B). As shown in Figure 2C, ANXA2 significantly soared in TA, G and Q muscles. However, ANXA2 did not change significantly in SOL muscle (Figure 2C). The ANXA2 RNA level also increased in TA and G muscles in aged mice (Figure S2C). Concurrently, the ANXA2 protein levels of liver, lung, kidney, spleen and heart did not show significant differences (Figure S2D). These results suggested that increased ANXA2 probably stemmed from secretion of muscles, especially fast muscles. Aged mice revealed a higher level of ANXA2 by immunofluorescence (Figure 2D). Notably, ANXA2 protein accumulated in the spaces between muscle fibres, suggesting its secretory nature. Similarly, aged people exhibited smaller CSA of muscles and higher ANXA2 level in muscle than young people (Figures S2E and 2D). After Dexamethasone (Dex) treatment, the myotubes formed by C2C12 cells turned thinner (Figure S2F). In parallel, the RNA levels of Atrogin‐1 and Muscle‐specific RING finger protein 1 (MuRF‐1) increased while Myogenin (MYOG) and Myogenic Differentiation 1 (MYOD) decreased (Figure S2G). At protein level, MuRF‐1 and Atrogin‐1 increased, whereas Myosin Heavy Chain (MyHC), MYOD and MYOG decreased (Figure S2H). All these results indicated that Dex could effectively induce atrophy of myotubes formed by C2C12 cells. As shown in Figure 2E, Dex treatment promoted production of ANXA2 in C2C12 myotubes at both RNA and protein levels. GW4869, an exosome inhibitor, did not repress the expression of ANXA2 in C2C12 myotubes (Figure 2E). Since ANXA2 is secreted by various cells [20], the ANXA2 in cell supernatant was examined by ELISA. Dex increased the ANXA level in cell supernatant and GW4869 inhibited its secretion (Figure S2I). To verify the secretion of ANXA2, we collected the serum samples from mice and humans. It was found that ANXA2 in serum of aged mice and humans both surged significantly (Figure S2J). Next, we investigated the ANXA2 level in cerebellums of young and aged mice. Immunofluorescence showed that there was a reduction in the number of Purkinje cells (marked by Calbindin) in Figure S2K. Elevated ANXA2 was also observed in Figure S2K, overlapping with the Purkinje cells (marked by Calbindin) but not with the astrocytes (marked by glial fibrillary acidic protein, GFAP). Likewise, Western Blot demonstrated higher expression of ANXA2 in aged cerebellums (Figure 2F). All these results proved that enhanced ANXA2 was possibly secreted by aged muscle tissues and transported towards cerebellum through blood, causing dysfunction of Purkinje cells.

### 3.3 ANXA2 Inhibits Myogenic Induction and Promotes Muscle Atrophy In Vitro

**FIGURE 3:**
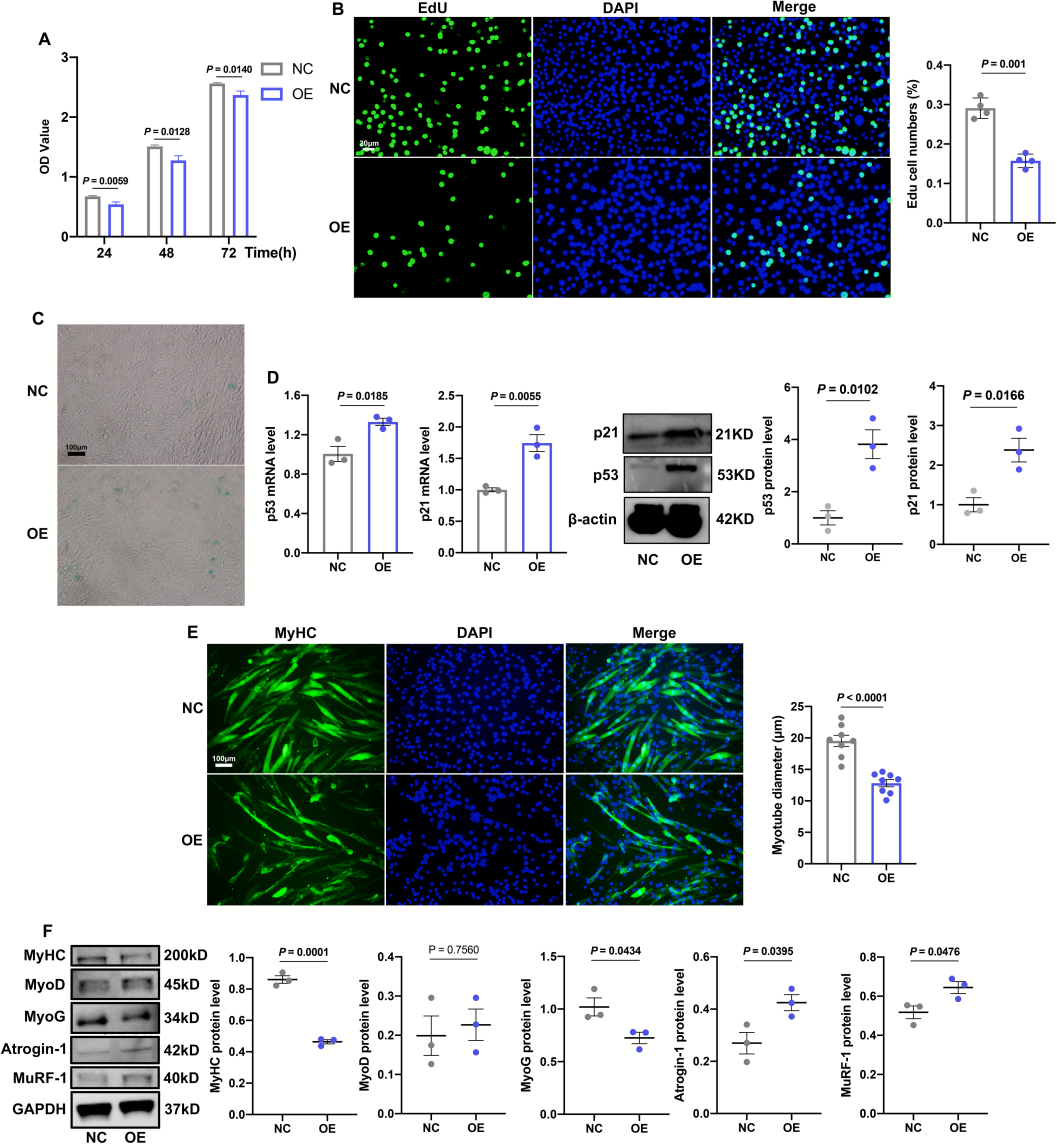
Effects of ANXA2 overexpression on proliferation, senescence and myogenic differentiation of C2C12 cells. (A) OD values of C2C12 cells in NC and OE groups at 24, 48 and 72 h by CCK‐8 (*n* = 8). (B) Representative images of EdU positive C2C12 cells in NC and OE groups and statistical results by immunofluorescence (*n* = 4). (C) S‐β‐Gal staining of C2C12 cells in NC and OE groups. (D) The RNA and protein levels and statistical results of p53 and p21 in C2C12 cells in NC and OE groups by qRT‐PCR and Western Blot (*n* = 3). (E) Representative images of MyHC staining of C2C12 myotubes in NC and OE groups and statistical results of diameters of myotubes by immunofluorescence (*n* = 8). (F) The protein levels and statistical results of MyHC, MyoD, MyoG, Atrogin‐1 and MuRF‐1 in C2C12 myotubes in NC an OE groups by Western Blot (*n* = 3). Values are represented as means ± SEM. Exact *p* values are shown.

To overexpress ANXA2, we constructed adenovirus‐encoding ANXA2 with Flag tag to infect C2C12 cells. As shown in Figure S3A, the high expression of EGFP indicated that the adenovirus had successfully infected the cells. Meanwhile, the RNA of ANXA and protein of Flag also increased, suggesting that ANXA2 was effectively overexpressed in C2C12 cells (Figure S3A). We first investigated the effects of ANXA2 overexpression on cell proliferation. The results of CCK‐8 showed that ANXA2 overexpression powerfully curbed cell proliferation of C2C12 cells within 72 h (Figure 3A). Furthermore, as shown in Figure 3B, the number of EdU+ cells in the ANXA2‐OE group was significantly lower than the control (NC) group. In terms of cell senescence, senescence β‐Galactosidase (S‐β‐Gal) staining showed that positive cells counted more in the OE group (Figure 3C). Besides, the aging proteins (p53, p21) also augmented, suggesting ANXA2 overexpression exacerbated cell senescence of C2C12 cells (Figure 3D). Then, we explored the effects of ANXA2 on myogenic differentiation. The width of myotubes, stained by MyHC, became thinner in the OE group (Figure 3E). The RNA levels of MYOG decreased while Atrogin‐1 and MuRF‐1 increased, although there was no significance in MYOD (Figure S3B). Similarly, the protein levels of MyHC and MyoG declined (no significance in MyoD), whereas MuRF‐1 and Atrogin‐1 climbed (Figure 3F).

In addition, we used recombinant ANXA2 (rA) to stimulate C2C12 cells or myotubes to detect the effects of exogenous ANXA2 on C2C12. Although rA led to the increase of RNA level of ANXA2, it did not cause changes in protein level in C2C12 cells (Figure S3C). rA also resulted in accumulation of S‐β‐Gal (Figure S3D) and elevated p53 and p21 (Figure S3E). As for the effects of rA on cell proliferation, 0.5 ng/mL rA inhibited cell proliferation at the third day, but 1 ng/mL rA showed inhibitory effect on cell proliferation within 3 days (Figure S3F). EdU assay also displayed that rA could hinder the proliferation at the concentration of 0.5 and 1 ng/mL (Figure S3G). Besides, we investigated the effects of rA on C2C12 myotubes. It was found that rA lowered the diameters of myotubes formed by C2C12 cells (Figure S3H), restrained MyoG and MyoD and upregulated MuRF‐1 and Atrogin‐1 at both RNA and protein levels (Figure S3I,J). All these results implied that both endogenous and exogenous ANXA2 promoted cell senescence, inhibited cell proliferation, repressed myogenic differentiation and exacerbated muscle atrophy.

### 3.4 ANXA2 Inhibits Myogenic Induction and Promotes Muscle Atrophy In Vivo

**FIGURE 4:**
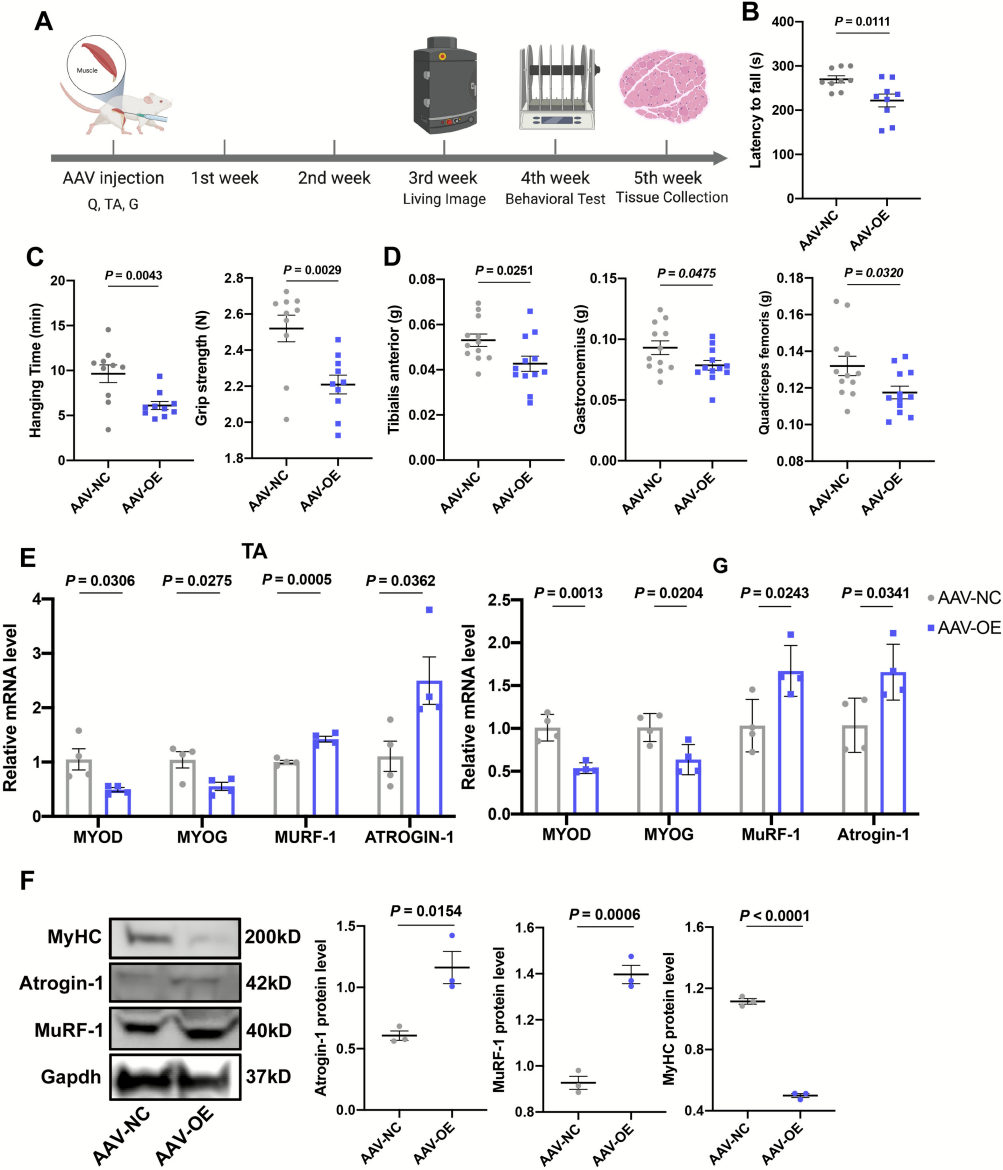
Effects of ANXA2 overexpression in muscles on behavioural performance. (A) Schematic diagram on AAV injection in muscles and subsequent experiments. ‘TA’ represents tibialis anterior; ‘Q’ represents quadriceps femoris; ‘G’ represents gastrocnemius. (B) AR latency to fall of mice in AAV‐NC and AAV‐OE groups (*n* = 9). (C) Hanging time and grip strength of mice in AAV‐NC and AAV‐OE groups in hanging grid tests (*n* = 10). (D) The mass of tibialis anterior, gastrocnemius and quadriceps femoris of mice in AAV‐NC and AAV‐OE groups (*n* = 12). (E) The RNA levels of MYOD, MYOG, MuRF‐1 and Atrogin‐1 in tibialis anterior (TA) and gastrocnemius (G) of mice in AAV‐NC and AAV‐OE groups by qRT‐PCR (*n* = 4). (F) The protein levels and statistical results of Atrogin‐1, MuRF‐1 and MyHC in tibialis anterior of mice in AAV‐NC and AAV‐OE groups by Western Blot (*n* = 3). Values are represented as means ± SEM. Exact *p* values are shown.

To evaluate the effects of ANXA2 on the performance of mice, we applied AAV (AAV8‐tMCK‐ANXA2‐Flag) to overexpress ANXA2 (AAV‐OE) in vivo. We performed a single intramuscular injection of AAV in the TA, G and Q muscles (Figure 4A). After 3 weeks, a living image system was used to assess the AAV infection efficiency. As shown in Figure S4A, the red fluorescence covered most parts of the lower limbs, suggesting successful infection. Then, we measured the physical performance at the fourth week. The motor coordination was impaired in mice in the AAV‐OE group, reflected by shorter latency to fall (Figure 4B), longer running time on 12 and 6‐mm balance beams (Figure S4B). The gait pattern was also altered by ANXA2 overexpression, manifesting as lower average speed and fewer cadences (Figure S4C). The gait coordination of AAV‐OE mice was like that of aged mice, namely, a smaller proportion of diagonal‐limb support and a larger proportion of three‐limb support (Figure S4D). The muscle strength declined, as evidenced by shorter hanging time and lower grip strength (Figure 4C). Correspondingly, the muscle masses dwindled significantly (Figure 4D) despite no significance in body mass (Figure S4E). We also found that ANXA2 increased in AAV‐OE mice at the molecular and histological levels (Figure S4F,G). Notably, the tag Flag was also detected in these muscles by Western Blot (Figure S4F). These results illustrated that AAV successfully upregulated ANXA2 in muscles. Meanwhile, ANXA2 rose in the liver and heart (Figure S4H). The protein expression of ANXA2 in the cerebellum was also elevated in the AAV‐OE group (Figure S4I,J). But the RNA of ANXA2 did not change in the cerebellum (Figure S4K). Notably, we also found that GFP was detected in the AAV‐OE group, suggesting that elevated ANXA2 protein in the cerebellum was transported from AAV‐infected muscles (Figure S4J). Simultaneously, the ANXA2 level in serum also increased in AAV‐OE mice (Figure S4L). We carried out further analysis of the influences of ANXA2 overexpression on muscle‐related markers. As shown in Figures 4E and S4M, MyoD and MyoG were downregulated, and MuRF‐1 and Atrogin‐1 were upregulated in the TA, G and Q muscles of AAV‐OE mice. Simultaneously, the protein levels of MuRF‐1 and Atrogin‐1 also elevated significantly (Figure 4F), accompanied by obviously reduced CSA (Figure S4N). We also performed loss‐of‐function studies in aged mice. After knocking down ANXA2 in muscles (shANXA2) (Figure S4O), the time on AR tests was obviously longer than that in the control group (shNC) (Figure S4P). Meanwhile, the strength performance improved, including hanging time and grip strength (Figure S4Q,R). Histologically, the myogenic markers (MYOD, MYOG) significantly increased in the shANXA2 group compared to the shNC group (Figure S4S). In terms of markers of muscle atrophy, the expression of Atrogin‐1 and MuRF‐1 significantly reduced in the shANXA2 group (Figure S4S). All these results implied that ANXA2 exacerbated muscle atrophy and impaired muscle function in vivo. Excessive ANXA2 protein could transfer to the cerebellum and cause motor incoordination.

### 3.5 ANXA2 Impairs Muscle Function via Downregulating Neu2

**FIGURE 5:**
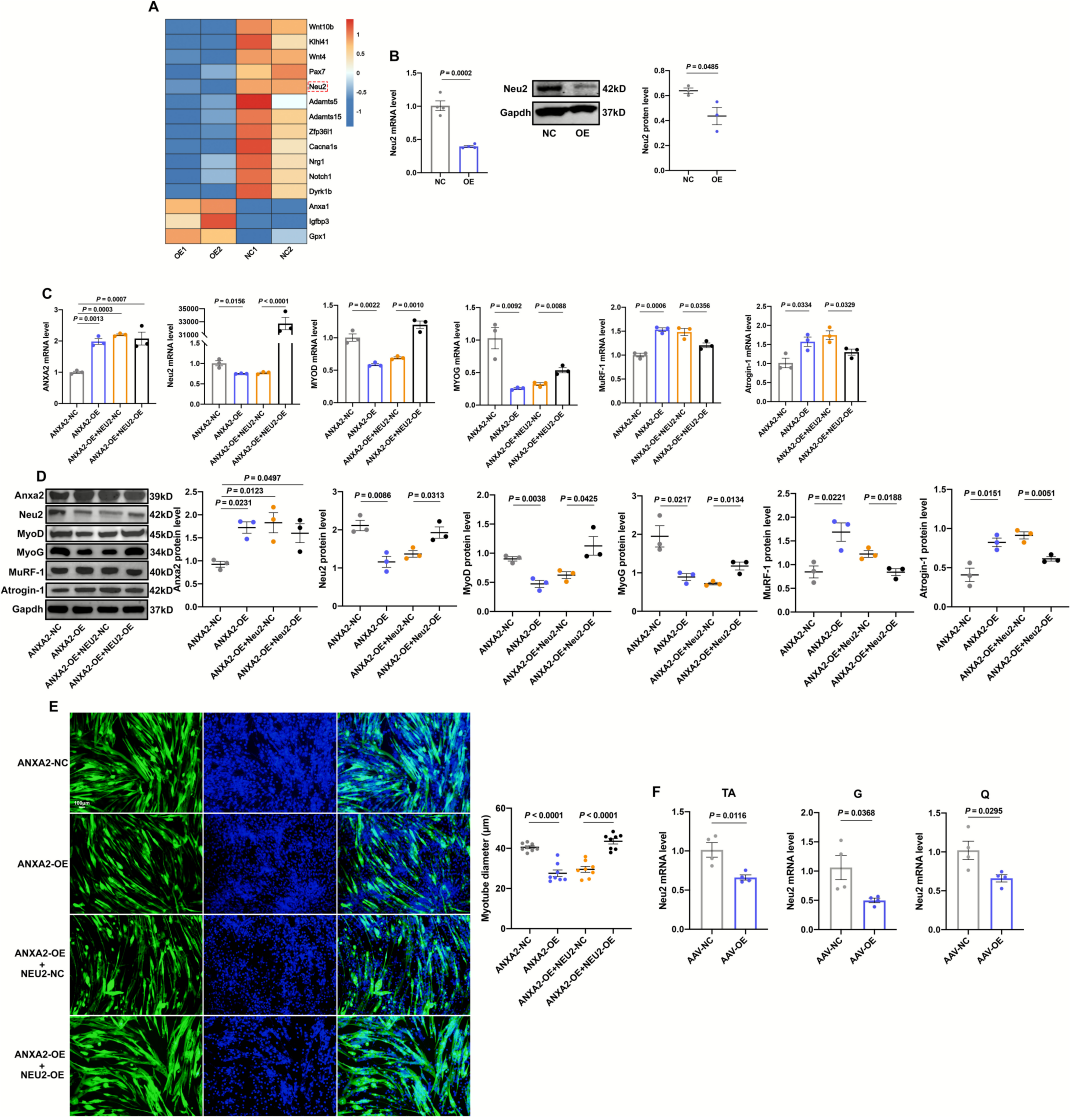
ANXA2 induces muscle atrophy via downregulating Neu2. (A) Heatmap of part of differential expressed genes in C2C12 myotubes in NC and OE groups. (B) The RNA and protein levels and statistical results of Neu2 in C2C12 myotubes in NC and OE groups by qRT‐PCR (*n* = 3–4). (C) The RNA levels of ANXA2, Neu2, MYOD, MYOG, MuRF‐1 and Atrogin‐1 in C2C12 myotubes in ANXA2‐NC, ANXA2‐OE, ANXA2‐OE + NEU2‐NC and ANXA2‐OE + NEU2‐OE groups by qRT‐PCR (*n* = 3). (D) The protein levels and statistical results of Anxa2, Neu2, MyoD, MyoG, MuRF‐1 and Atrogin‐1 in C2C12 myotubes in ANXA2‐NC, ANXA2‐OE, ANXA2‐OE + NEU2‐NC and ANXA2‐OE + NEU2‐OE groups by Western Blot (*n* = 3). (E) Representative images of MyHC staining of C2C12 myotubes in ANXA2‐NC, ANXA2‐OE, ANXA2‐OE + NEU2‐NC and ANXA2‐OE + NEU2‐OE groups and statistical results of diameters of myotubes by immunofluorescence (*n* = 8). (F) The RNA levels of NEU2 in tibialis anterior (TA), gastrocnemius (G), quadriceps femoris (Q) of mice in AAV‐NC and AAV‐OE groups by qRT‐PCR (*n* = 4). Values are represented as means ± SEM. Exact *p* values are shown.

To explore the mechanism of the effects of ANXA2 on muscles, we employed RNA‐sequencing. As shown in Figure S5A, ANXA2 overexpression in C2C12 myotubes led to 1255 upregulated genes and 1558 downregulated genes. Thereinto, numerous muscle‐related genes changed, suggesting ANXA2 regulated muscle functions (Figure S5B). Neuraminidase 2 (Neu2), required for lactate‐mediated myoblast differentiation [21], was obviously inhibited in the OE group (Figure 5A). The results of RT‐qPCR and Western Blot verified that ANXA2 overexpression inhibited Neu2 (Figure 5B). To confirm the relation between ANXA2 and Neu2, we used siRNA to lower the expression of ANXA2. siRNA effectively inhibited ANXA2 (Figure S5C). Overall, we selected the first sequence of siRNA (si1) for the following experiments. Contrary to ANXA2 overexpression, inhibiting ANXA2 led to upregulation of MyoD and MyoG and downregulation of MuRF‐1 and Atrogin‐1 (Figure S5D). siANXA2 also resulted in wider myotubes, indicating that inhibition of ANXA2 promoted muscle differentiation and ameliorated muscle atrophy (Figure S5E). Based on these results, we found that knockdown of ANXA2 was related to the increase of Neu2 (Figure S5F). We also explored the effects of Neu2 inhibition on C2C12. Based on the results of siRNA, we chose the third sequence for the following experiments (Figure S6A). Identical to ANXA2 overexpression, MyoD and MyoG decreased, whereas MuRF‐1 and Atrogin‐1 increased (Figure S6B,C). Neu2 knockdown inhibited myotubes as well (Figure S6D).

After, Figure 5C showed that Neu2‐overexpression (by plasmid, Neu2‐OE) based on ANXA2‐overexpression could upregulate Neu2. In the meantime, Neu2‐overexpression helped upregulate MyoD and MyoG and downregulate MuRF‐1 and Atrogin‐1 (Figure 5C,D). Likewise, Neu2 overexpression also increased the shortened diameters of myotubes caused by ANXA2 overexpression (Figure 5E). Besides, we found that the expression of Neu2 in muscles in ANXA2‐OE mice also dwindled (Figure 5F). All these results suggested that ANXA2 overexpression led to Neu2 inhibition and further caused muscle atrophy and inhibition of myogenic differentiation.

### 3.6 ANXA2 Causes Motor Incoordination

**FIGURE 6:**
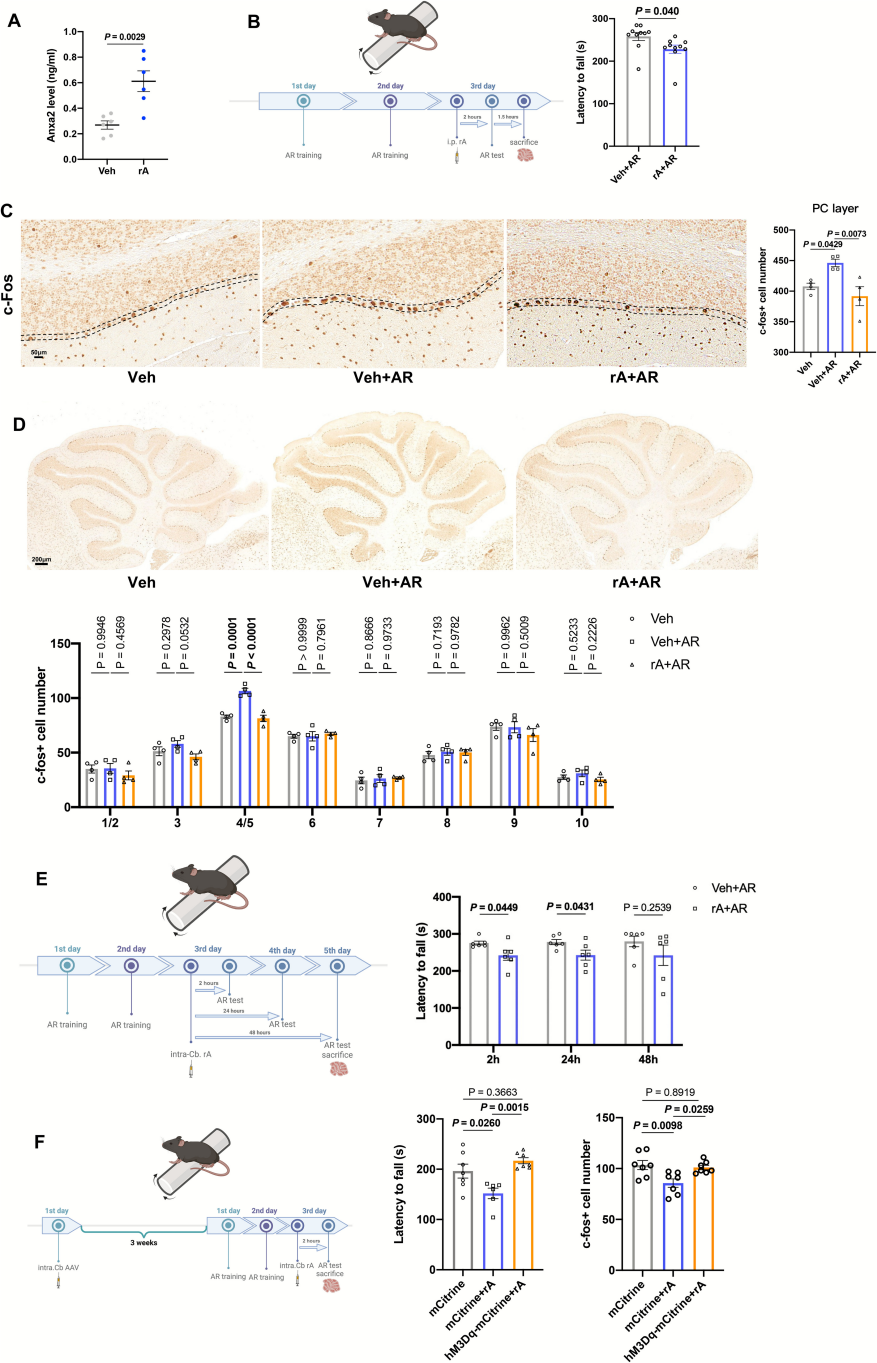
Effects of ANXA2 on motor coordination. (A) The ANXA2 level of serum in mice after intraperitoneal (i.p.) injection of saline (Veh) and rA (rA) by ELISA (*n* = 6). (B) Schematic diagram on i.p. injection of rA and timeline of AR tests (left) and AR latency to fall of mice after i.p. injection of saline (Veh + AR) and rA (rA + AR) (right) (*n* = 10). (C) Representative images (left) and numbers (right) of c‐Fos positive cells in Purkinje (PC) layers of mice in Veh, Veh + AR, rA + AR groups (*n* = 4). (D) Representative images (up) and numbers (down) of c‐Fos positive cells in PC layers of various cerebellar lobules in mice in Veh, Veh + AR, rA + AR groups (*n* = 4). (E) Schematic diagram on intracerebellumal (intra. Cb.) injection of rA and timeline of AR tests (left) and AR latency to fall of mice after intra. Cb. injection of saline (Veh + AR) and rA (rA + AR) (right) (*n* = 6–7). (F) Schematic diagram on intra. Cb. injection of AAV9‐L7‐6‐hM3D(Gq)‐mCitrine and rA and timeline of AR tests (left) and effects of virus and rA on AR latency to fall (middle) and numbers of c‐Fos positive cells (right) (*n* = 7). Values are represented as means ± SEM. Exact *p* values are shown.

We performed intraperitoneal (i.p.) injection of rA and found that the ANXA2 level in serum was elevated after rA i.p. injection (Figure 6A). rA injection also raised the ANXA2 level of cerebellum (Table S1). Meanwhile, i.p. injection of rA decreased latency to fall of mice (Figure 6B). Compared with vehicle (Veh) group, AR test increased the number of c‐Fos+ cells, whereas rA inhibited activation of Purkinje cells (PC) (Figure 6C). Dorsomedial striatum (DMS) and motor cortex (M) are key to motor coordination [22]. The number of c‐Fos+ cells in DMS, primary motor cortex (M1) and secondary motor cortex (M2) showed no significant difference in Figure S7A–F. Then, we further analysed the number of c‐Fos+ cells in different lobules. Notably, the c‐Fos+ cell number altered obviously in the cerebellum 4/5 lobule (4/5Cb) (Figure 6D). Thus, we injected rA into 4/5Cb through stereotactic injection (Figure 6E). At 2 and 24 h after injection, rA intra‐cerebellum injection significantly inhibited motor coordination. But the inhibitory effect disappeared at 48 h (Figure 6E).

To further verify the effects of rA on Purkinje cells in 4/5Cb, we constructed Purkinje cells–specific AAV (AAV9‐L7‐6‐hM3D(Gq)‐mCitrine) to activate Purkinje cells via clozapine *N*‐oxide (CNO) (Figure 6F). The fluorescence of mCitrine was explored to confirm the successful infection of AVV (Figure S7G). Our results showed that activation of Purkinje cells in 4/5Cb could attenuate the inhibitory effects of rA and increase the c‐Fos+ cells inhibited by rA (Figure 6F). Taken together, all these results suggested 4/5Cb is a pivotal brain region for the inhibitory effects of rA on motor coordination.

### 3.7 Cannabinoid Receptor 2 (CB2R) in the 4/5Cb Contributes to the Motor Incoordination Caused by ANXA2

**FIGURE 7:**
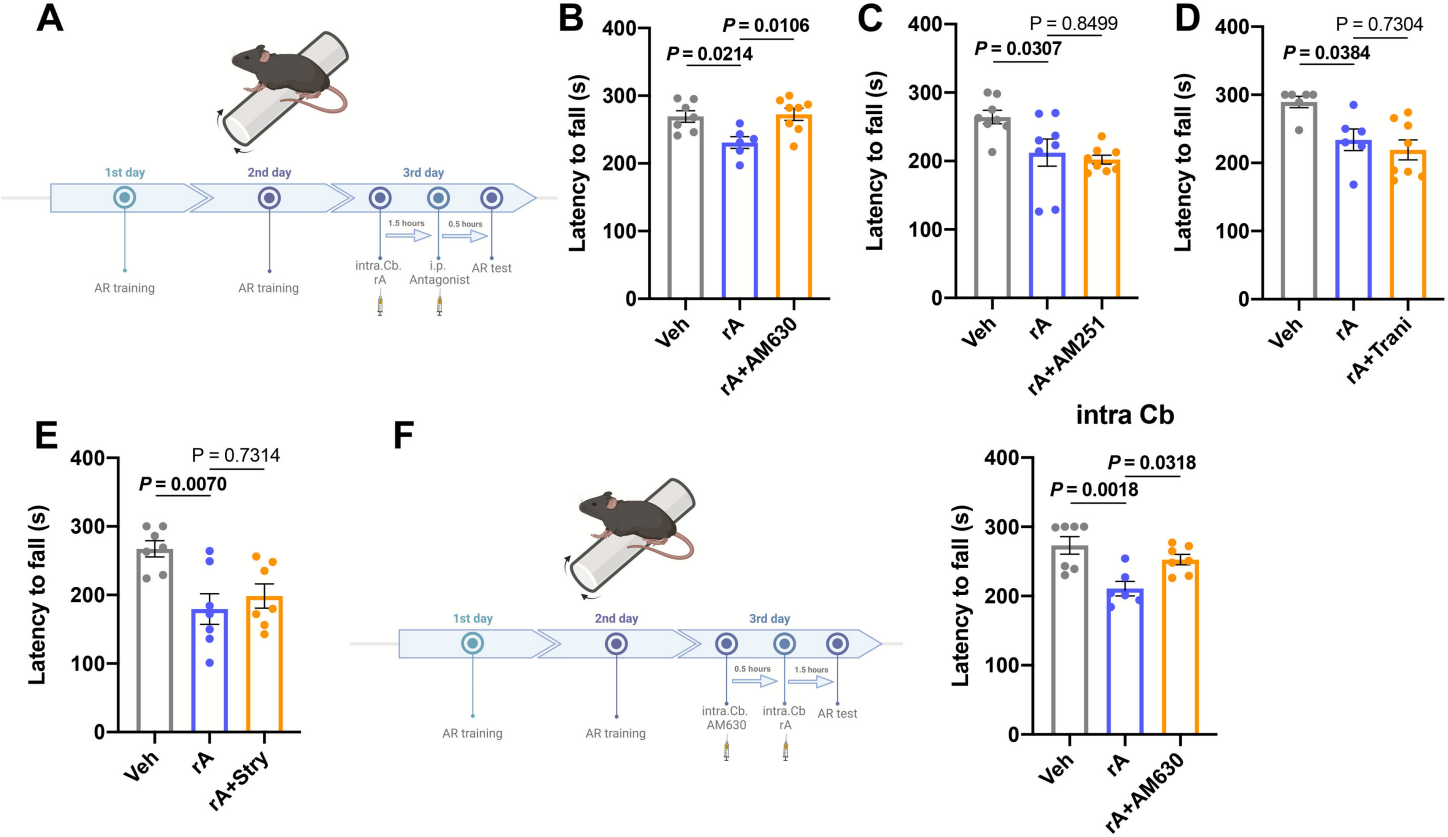
Pharmacological identification of ANXA2 targets in the 4/5 Cb. (A) Schematic diagram on the timeline of systemic drug administration and AR procedure. (B) Effects of AM630 on motor incoordination induced by ANXA2 in mice (*n* = 6–8). (C) Effects of AM251 on motor incoordination induced by ANXA2 in mice (*n* = 8). (D) Effects of Tranilast (Trani) on motor incoordination induced by ANXA2 in mice (*n* = 6 or 8). (E) Effects of Strychnine (Stry) on motor incoordination induced by ANXA2 in mice (*n* = 7). (F) Schematic diagram on the timeline of intra 4/5 Cb drug administration and AR procedure (left) and effects of AM630 via intra 4/5 Cb injection on motor incoordination induced by ANXA2 in mice (right) (*n* = 7). Values are represented as means ± SEM. Exact *p* values are shown.

We next investigated potential targets involved in motor incoordination caused by ANXA2 (Figure 7A). On cannabinoid receptors, we found that systemic administration of the CB2R antagonist AM630 diminished motor incoordination induced by ANXA2 (Figures 7B and S8A), whereas the CB1R antagonist AM251 did not improve (Figures 7C and S8B). In addition, TRPV2 antagonist tranilast (Figures 7D and S8C) and GlyR antagonist strychnine (Figures 7E and S8D) also did not enhance motor incoordination induced by rA. We next performed local microinjection to examine the role of CB2R in the 4/5Cb in the motor coordination induced by rA (Figure 7F). Intra‐4/5Cb injection of AM630 significantly alleviated motor incoordination caused by rA (Figure 7F). To further verify the function of CB2R, we injected AAV stereotactically to knock down the expression of CB2R (Figure S8E). It was found that mice in the rA + AAV‐shNC group showed worse performance in AR test than mice in the Veh group. Simultaneously, the stay time of mice in the rA + AAV‐shCB2R group was longer than that of mice in the rA + AAV‐shNC group (Figure S8F). Taken together, these results indicated that CB2R in the 4/5Cb may acted as the primary targets of ANXA2 to cause incoordination.

### 3.8 Isoliquiritigenin (ISL) Attenuates Motor Incoordination in Aged Mice

**FIGURE 8:**
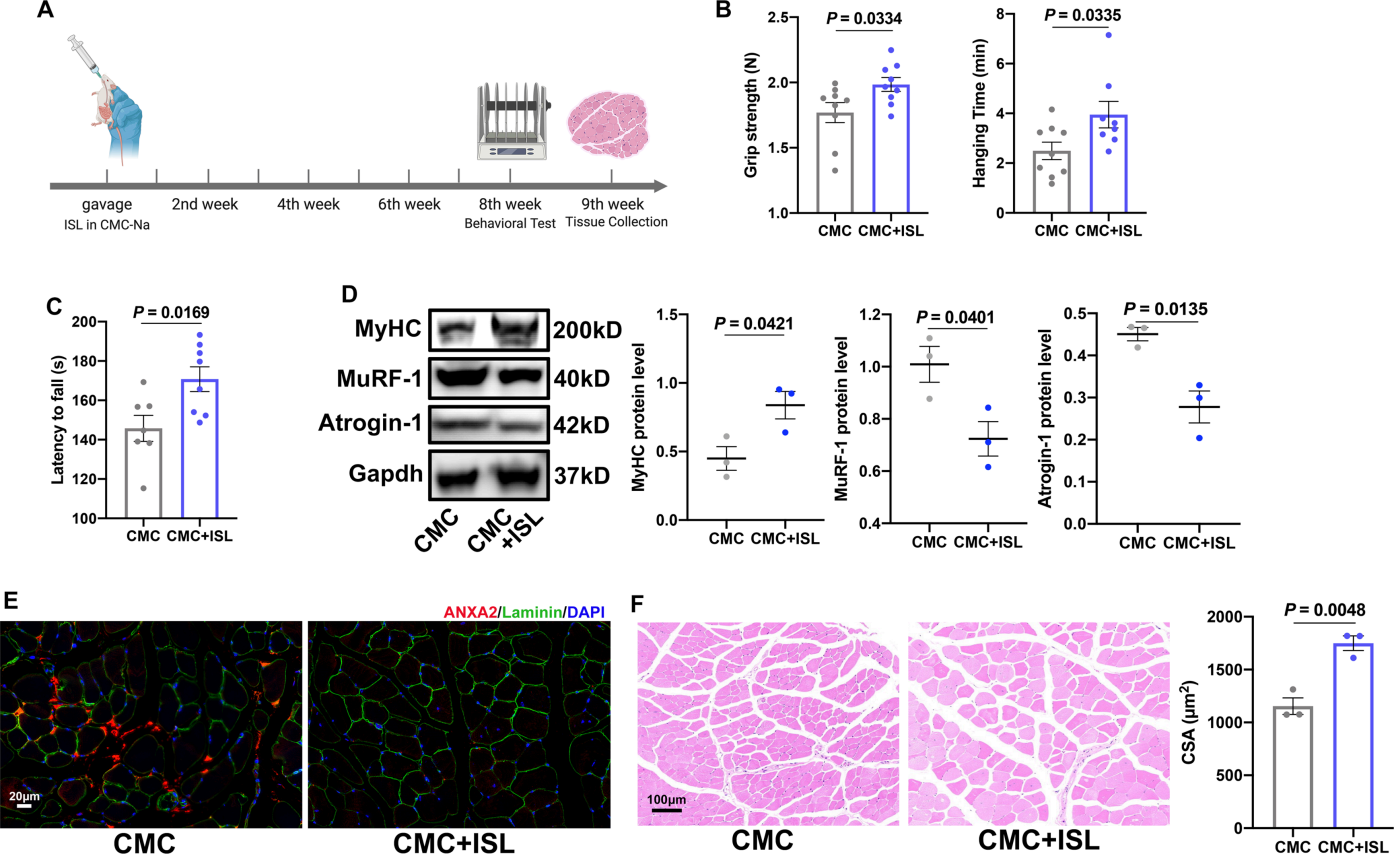
Effects of isoliquiritigenin (ISL) on motor coordination and muscle function of aged mice. (A) Schematic diagram on the timeline administration of ISL by gavage on aged mice. (B) The grip strength (left) and hanging time in hanging grid test (right) of mice in CMC and CMC + ISL groups (*n* = 8–9). (C) AR latency to fall of mice in CMC and CMC + ISL groups (*n* = 7–8). (D) The protein levels and statistical results of MyHC, MuRF‐1, and Atrogin‐1 in muscles of mice in CMC and CMC + ISL groups by Western Blot (*n* = 3). (E) The ANXA2 expression in muscles of mice in CMC and CMC + ISL groups by immunofluorescence. (F) Representative images and cross sectional area (CSA) of tibialis anterior of mice in CMC and CMC + ISL groups (*n* = 3). Values are represented as means ± SEM. Exact *p* values are shown. CMC represents 0.5% sodium carboxyl methyl cellulose (CMC‐Na). CMC + ISL represents 20 mg/kg ISL (in 0.5% CMC‐Na).

Isoliquiritigenin (ISL) was reported to suppress ANXA2, alleviating the development of alcoholic liver fibrosis [23]. ISL is a flavonoid compound extracted from licorice (Figure S9A). It was shown that 0.5 ng/mL ISL (Dex + LISL group) and 1 ng/mL ISL (Dex + HISL group) both could effectively reduce ANXA2 induced by Dex at RNA and protein level (Figure S9B). Based on these findings, we explored the in vivo effects of ISL in aged mice. We administered ISL by gavage for 8 weeks in 20‐month mice and evaluated its effects on a series of behavioural tests (Figure 8A). Although ISL showed toxicity on various cancer cells, it is safe at proper concentrations [24]. In this study, we found 20 mg/kg ISL (in 0.5% w/v sodium carboxyl methyl cellulose, CMC‐Na) showed no obvious impacts on the weights of mice (Figure S9C). ISL increased grip strength and hanging time of aged mice (Figure 8B). At the organ level, the mice in the CMC + ISL group exhibited higher weights of TA, G and Q muscles than those in the CMC group (Figure S9D). As for the performance of motor coordination, the mice in the CMC + ISL group showed better performance in AR rotarod tests (Figure 8C) and balance beam tests (Figure S9E). Mice in CMC + ISL group showed higher gait speeds and cadences (Figure S9F). Notably, the duration of diagonal support increased, whereas the duration of three‐limb support decreased (Figure S9G). Histologically, the expression of MyHC was higher, whereas the expression of MuRF‐1 and Atrogin‐1 was lower in the CMC + ISL group (Figure 8D). We also detected the expression of ANXA2 in the muscles. As shown in Figure 8E, ISL effectively inhibited the ANXA2 in muscles. Meanwhile, the CSA of the muscles also grew following ISL treatment (Figure 8F). Notably, ANXA2 also decreased in the cerebellums and serum after ISL gavage (Figure S9H,I).

Since CB2R is involved in endogenous anti‐anxiolytic activity [25], we explored the symptoms of anxiety in the mice. It was found that ISL had no impairments on the time in centre zone, four corners, four sides and total distance, centre distance, distance in four corners and four sides in open field tests (Figure S9J) and the time in open arms, centre and closed arms in elevated plus maze tests (Figure S9K). Furthermore, ISL did not affect the food intake (Figure S9L) and the histological performance of heart, liver, kidney, spleen and lung (Figure S10). Taken together, ISL is a promising and effective medication with excellent biosafety for motor incoordination in patients with sarcopenia.

## 4 Discussion

The present study reveals a significant muscle–cerebellum axis, in which upregulation of ANXA2 in aged muscles contributes to aging‐associated motor incoordination via the cerebellum. We found that ANXA2 was elevated in both aged skeletal muscles and cerebellums, but not in the other aged tissues. Local overexpression of ANXA2 further aggravated muscle atrophy and motor deficits. Importantly, ISL effectively suppressed ANXA2 expression and alleviated motor incoordination in aged mice. These findings extend the current understanding of motor dysfunction in sarcopenia and highlight herbal therapy targeting ANXA2 with ISL as a promising strategy to improve balance and reduce fall risks in sarcopenia patients.

We investigated muscle mass and muscle strength in our mouse models of sarcopenia. Both parameters were significantly reduced in aged mice, confirming the presence of sarcopenia. Given that motor incoordination and imbalance are widely reported in patients with sarcopenia [1], we further examined motor coordination in these mice. Compared with young controls, aged mice showed poorer performance, indicating impaired coordination. Gait analysis revealed not only reduced speed and cadence but also an increased proportion of three‐ or four‐limb support and a decreased proportion of single‐ or double‐limb support. These alterations suggested motor incoordination during gait in aged mice, reflecting a compensatory need for greater limb support to maintain stability, a pattern consistent with gait abnormalities observed in humans [26].

ANXA2 is a key member of the annexin family and is prominently expressed on the surface of various cell types [27]. It has been extensively studied in numerous cancers, promoting proliferation and invasion [28]. ANXA2 also plays a role in age‐related diseases. MicroRNA‐425‐5p could modulate osteoporosis by targeting ANXA2 [29]. In our study, proteomic analysis revealed that ANXA2 levels were elevated in both aged skeletal muscles and cerebellum. ANXA2 has been identified as a secretory protein and is known to be present in small extracellular vesicles [30]. Although ANXA2 is expressed in multiple tissues, we observed differential expression only in muscles. Considering that muscle atrophy is a hallmark of sarcopenia, we speculated that the elevated serum ANXA2 was secreted by aged muscles and subsequently transported to the cerebellum, where its levels were also increased.

Upregulation of ANXA2 promotes proliferation and invasion of breast cancer MCF‐7 cells [31]. In this study, ANXA2 overexpression inhibited C2C12 proliferation and promoted cellular senescence. As proliferation of myoblasts is a prerequisite for muscle regeneration and hypertrophy [32], the inhibitory effect of ANXA2 on C2C12 proliferation may contribute to muscle atrophy, contrasting with its pro‐proliferative role in cancer cells. In addition to proliferation, ANXA2 was found to suppress myogenic differentiation and promote muscle atrophy, highlighting its detrimental role in sarcopenia, regardless of whether it originates endogenously or exogenously. Consistently, overexpression of ANXA2 in multiple muscles via AAV in young mice led to decreased muscle mass and strength, recapitulating sarcopenia‐like symptoms.

Mechanistically, we found ANXA2 upregulation caused downregulation of Neu2, a mammalian cytosolic sialidase [33]. Neu2 has been reported to promote myoblast differentiation [34]. Beyond its direct role, insulin‐like growth factor‐1 (IGF‐1) can induce myoblast differentiation and hypertrophy through Neu2 upregulation [35], and lactate, a key muscle metabolite, promotes myogenesis via H3K9 lactylation‐dependent Neu2 expression [21]. Together, these findings underscore the critical role of Neu2 in myogenesis. Consistent with this, our results demonstrated that ANXA2 inhibited Neu2, thereby suppressing myogenesis and exacerbating muscle atrophy, whereas ANXA2 knockdown enhanced myogenesis through Neu2 upregulation.

Local administration of ANXA2‐overexpression AAV not only increased ANXA2 expression in muscle tissues but also raised its expression in other organs, including heart, liver, serum and cerebellum. Notably, ANXA2 RNA levels did not differ significantly, supporting the notion that the protein is secreted rather than transcriptionally upregulated in these tissues. Given the secretory characteristics of ANXA2, these findings suggest that ANXA2 is released from aged muscles into the bloodstream, transported to the cerebellum and influences cerebellar function. To assess this, we evaluated motor coordination using behavioural tests. ANXA2‐overexpressing mice displayed motor deficits similar to those observed in aged mice, including shorter rotarod endurance and longer balance‐beam traversal times. Collectively, these results indicate that muscle‐derived ANXA2 can reach the cerebellum and contribute to motor incoordination.

In central nervous system, motor coordination is primarily regulated by the DMS, motor cortex and cerebellum [22]. In our study, we observed that neuronal activation, as indicated by the number of c‐Fos+ cells, was altered only in the cerebellum—particularly within the PC layer of lobules IV/V, which were known to regulate motor coordination [36]. Consistently, local injection of rA further confirmed the involvement of lobules IV/V in motor incoordination. Purkinje cell protein 2 (Pcp2, also known as L7) is abundantly expressed in PCs [37], and we therefore employed the L7 promoter to construct a PC‐specific AAV [38]. Using chemogenetic approaches, we validated that lobules IV/V represent the critical cerebellar targets of ANXA2.

In the present study, presynaptic CB2Rs were identified as critical targets of rA‐induced motor incoordination. Pharmacological blockade of CB2Rs partially reversed the neuronal hyperexcitability triggered by rA and alleviated motor incoordination. However, whether other receptors—such as cerebellar adenosinergic A1 receptors or nicotinic cholinergic receptors—also contribute to ANXA2‐induced motor incoordination remains unclear and warrants further investigation [39].

According to previous studies, ISL has been shown to effectively inhibit ANXA2 in alcoholic liver fibrosis [23]. Building on this evidence, we examined the effects of ISL on muscle atrophy and motor coordination in sarcopenia. After 2 months of treatment in aged mice, ISL markedly attenuated muscle atrophy and improved motor coordination. Collectively, these findings suggest that ISL is a promising therapeutic candidate for ameliorating motor incoordination associated with sarcopenia.

Previous studies have highlighted the importance of muscle–brain axis, particularly focusing on myokines such as irisin that mediate muscle–cortex interactions. However, the role of muscle‐derived factors in regulating cerebellar function has remained largely unexplored. In this study, we provide evidence supporting a muscle–cerebellum axis, in which ANXA2 secreted from muscles enters the circulation, accumulates in the cerebellum and modulates motor function through CB2R signalling. These findings extend the concept of peripheral–central communication beyond the cerebral cortex to the cerebellum, thereby offering novel mechanistic insights into neuromuscular regulation in sarcopenia. Moreover, the identification of ISL as a modulator of ANXA2 adds translational relevance and suggests new therapeutic opportunities targeting this axis.

Several limitations should be acknowledged in our manuscript. First, the precise route and mechanisms by which ANXA2 is transported from muscle to the cerebellum remain incompletely understood, including how ANXA2 is secreted into the bloodstream, circulates systemically and crosses the blood–brain barrier. Second, the molecular basis of ANXA2‐mediated Neu2 regulation, such as the involvement of transcriptional repressors or chromatin modifications, has not yet been clearly delineated. Third, the mechanisms underlying the interaction between ANXA2 and CB2R require further investigation. In addition, how ISL inhibits ANXA2 represents another important question that merits future study.

In conclusion, ANXA2 is secreted by atrophic muscles and transported to the cerebellum, where it targets Purkinje cells in lobule IV/V and contributes to motor incoordination. ISL, a natural compound derived from licorice, suppresses ANXA2 expression in muscle, thereby alleviating muscle atrophy and improving motor coordination.

## Funding

This work was supported by the National Key Research and Development Program of China (2023YFC2414102, 2022YFC2009502), the National Natural Science Foundation of China (82172402), the Clinical Research Program of the 9th People's Hospital, Shanghai Jiao Tong University School of Medicine (JYLJ202101) and the Shanghai Key Laboratory of Orthopedic Implants (KFKT202210).

## Conflicts of Interest

The authors declare no conflicts of interest.

## Supporting information


**Data S1:** Supporting information.

**Data S2:** Supporting information.

**Figure S1:** Motor coordination and muscle function of aged mice. Related to Figure 1. (A) Schematic diagram on the timeline of balance beam test procedure (left) and running time of young and aged mice on 12 mm (middle) and 6 mm (right) balance beam tests (*n* = 10). (B) Schematic diagram on the timeline of pole test procedure (left) and running time (middle) and total time (right) of young and aged mice in pole test (*n* = 10). (C) The average speed (left), body speed (middle) and swing speed (right) of young and aged mice in gait analysis (*n* = 8–9). (D) Cadence (left), stand time (right) and swing time (right) of young and aged mice (*n* = 8–9). (E) Support proportion of young and aged mice in gait analysis (*n* = 8–9). (F) The masses of extensor digitorum longus, soleus and plantaris muscles of young and aged mice (*n* = 10). (G) Representative images and cross sectional area (CSA) of gastrocnemius in young and aged mice (*n* = 3). Values are represented as means ± SEM. Exact *p* values are shown. RF represents right front. RH represents right hind. LF represents left front. LH represents left hind.
**Figure S2:** ANXA2 increased both in muscles and cerebellums of aged mice. Related to Figure 2. (A) Heatmap (left) and volcano plot (right) of differential expressed proteins of muscles in young and aged mice. (B) Heatmap (left) and volcano plot (right) of differential expressed proteins of cerebellums in young and aged mice. (C) The RNA levels of ANXA2 in tibialis anterior (TA, left) and gastrocnemius (G, right) in young and aged mice by qRT‐PCR (*n* = 3). (D) The protein levels of ANXA2 in liver, lung, kidney, spleen and heart in young and aged mice (left) and statistical results (right) by Western Blot (*n* = 4 or 6). (E) HE staining of TA in young (Young‐H) and aged (Aged‐H) human. (F) Representative images of MyHC staining of C2C12 myotubes in Mock and Dexamethasone (Dex) groups (left) and statistical results of diameters of myotubes by immunofluorescence (right) (*n* = 8). (G) The RNA levels of Atrogin‐1, MuRF‐1, MYOG and MYOD in C2C12 myotubes in Mock and Dex groups by qRT‐PCR (*n* = 3). (H) The protein levels and statistical results of Atrogin‐1, MuRF‐1, MyHC, MyoG and MyoD in C2C12 myotubes by Western Blot (*n* = 3–4). (I) The level of ANXA2 in supernatant of C2C12 myotubes in Mock, Dex and Dex + GW4869 groups by ELISA (*n* = 3). (J) The level of ANXA2 in mice (Young‐M represents young mice, Aged‐M represents aged mice) and human (Young‐H represents young human, Aged‐H represents aged human) serum by ELISA (*n* = 7; *n* = 17 or 16). (K) The expression of ANXA2 in cerebellums from young and aged mice by immunofluorescence. Values are represented as means ± SEM. Exact *p* values are shown. ‘A’ represents aged mice, ‘Y’ represents young mice.
**Figure S3:** Effects of exogenous ANXA2 (recombinant ANXA2, rA) on proliferation, senescence of C2C12 cells and atrophy of C2C12 myotubes. (A) EGFP fluorescence of adenovirus (left) and the RNA level of ANXA2 (middle) and protein level of Flag (right) in C2C12 cells in NC and OE groups (*n* = 3). (B) The RNA levels of MYOD, MYOG, Atrogin‐1 and MuRF‐1 in C2C12 myotubes in NC and OE groups by qRT‐PCR (*n* = 3). (C) The RNA (left) and protein (middle) levels and statistical results (right) of ANXA2 in C2C12 cells in C, 0.5 ng/mL rA and 1 ng/mL rA groups (*n* = 3). (D) S‐β‐Gal staining of C2C12 cells in C, 0.5 ng/mL rA and 1 ng/mL rA groups. (E) The RNA and protein levels and statistical results of p53 and p21 in C2C12 cells in C, 0.5 ng/mL rA and 1 ng/mL rA groups (*n* = 3). (F) OD values of C2C12 cells in C, 0.5 ng/mL rA and 1 ng/mL rA groups by CCK‐8 (*n* = 6). (G) Representative images of EdU positive C2C12 cells in C, 0.5 ng/mL rA and 1 ng/mL rA groups (left) and statistical results (right) by immunofluorescence (*n* = 3). (H) Representative images of MyHC staining of C2C12 myotubes in C, 0.5 ng/mL rA and 1 ng/mL rA groups (left) and statistical results of diameters of myotubes (right) by immunofluorescence (*n* = 8). (I) The RNA levels of MYOG, MYOD, MuRF‐1 and Atrogin‐1 in C2C12 myotubes in C, 0.5 ng/mL rA and 1 ng/mL rA groups by qRT‐PCR (*n* = 3). (J) The protein levels and statistical results of MyHC, MyoD, MyoG, Atrogin‐1 and MuRF‐1 in C2C12 myotubes in C, 0.5 ng/mL rA and 1 ng/mL rA groups by Western Blot (*n* = 3). Values are represented as means ± SEM. Exact *p* values are shown. ‘C’ represents control group. ‘0.5 ng/mL’ represents 0.5 ng/mL rA group. ‘1 ng/mL’ represents 1 ng/mL rA group.
**Figure S4:** Effects of ANXA2 overexpression in muscles on behavioural performance. Related to Figure 4. (A) Representative fluorescence images of mice at the 3rd week after AAV intramuscular injection. (B) Running time of mice in AAV‐NC and AAV‐OE groups on 12 mm (left) and 6 mm (right) balance beam tests (*n* = 10). (C) The average speed (left) and cadence (right) of mice in AAV‐NC and AAV‐OE groups in gait analysis (*n* = 9). (D) The support proportion of mice in AAV‐NC and AAV‐OE groups in gait analysis (*n* = 9). (E) Body mass of mice in AAV‐NC and AAV‐OE groups (*n* = 10). (F) The RNA and protein levels of ANXA2 in tibialis anterior (TA), gastrocnemius (G) and quadriceps femoris (Q) of mice in AAV‐NC and AAV‐OE groups. (G) The expression of GFP, ANXA2 and laminin in TA of mice in AAV‐NC and AAV‐OE groups by immunofluorescence. (H) The protein levels of heart (left) and liver (right) of mice in AAV‐NC and AAV‐OE groups. (I) The protein level of ANXA2 in cerebellums of mice in AAV‐NC and AAV‐OE groups. (J) The expression of GFP, ANXA2 and Calbindin in cerebellums of mice in AAV‐NC and AAV‐OE groups by immunofluorescence. (K) The RNA levels of cerebellum of mice in AAV‐NC and AAV‐OE groups by qRT‐PCR (*n* = 3). (L) The ANXA2 level in serum of mice in AAV‐NC and AAV‐OE groups by ELISA (*n* = 6). (M) The RNA levels of MYOD, MYOG, MuRF‐1 and Atrogin‐1 in quadriceps of mice in AAV‐NC and AAV‐OE groups by qRT‐PCR (*n* = 4). (N) Representative images (left) and cross sectional area (CSA, right) of TA of mice in AAV‐NC and AAV‐OE groups (*n* = 3). (O) The RNA and protein level of ANXA2 in muscles of mice in shNC and shANXA2 groups. (P) AR latency to fall of mice in shNC and shANXA2 groups (*n* = 6). (Q) Hanging time of mice in shNC and shANXA2 groups (*n* = 6). (R) Grip strength of mice in shNC and shANXA2 groups (*n* = 6). (S) The RNA levels of MYOD, MYOG, Atrogin‐1 and MuRF‐1 in muscles of mice in shNC and shANXA2 groups (*n* = 4). Values are represented as means ± SEM. Exact *p* values are shown.
**Figure S5:** Effects of ANXA2 knockdown on myogenic differentiation of C2C12 cells. Related to Figure 5. (A) Volcano plot of differential expressed genes in C2C12 myotubes in NC and OE groups. (B) Heatmap of differential expressed genes related to muscles in C2C12 myotubes in NC and OE groups. (C) The RNA (left) and protein (middle) levels and statistical results (right) of ANXA2 in C2C12 cells in siNC, siANXA2‐1, siANXA2‐2 and siANXA2‐3 groups by qRT‐PCR and Western Blot (*n* = 3–4). (D) The RNA and protein levels and statistical results of MYOG, MYOD, MuRF‐1 and Atrogin‐1 in C2C12 myotubes in siNC and siANXA2 groups by qRT‐PCR and Western Blot (*n* = 3–4). (E) Representative images of MyHC staining of C2C12 myotubes in siNC and siANXA2 groups (left) and statistical results of diameters of myotubes (right) by immunofluorescence (*n* = 8). (F) The RNA (left) and protein (middle) levels and statistical results (right) of Neu2 in C2C12 myotubes in siNC and siANXA2 groups (*n* = 3–4). Values are represented as means ± SEM. Exact *p* values are shown.
**Figure S6:** Effects of NEU2 knockdown on myogenic differentiation of C2C12 cells. Related to Figure 5. (A) The RNA (left) and protein (middle) levels and statistical results (right) of NEU2 in C2C12 cells in siNC, siNEU2‐1, siNEU2‐2 and siNEU2‐3 groups by qRT‐PCR and Western Blot (*n* = 3). (B) The RNA levels of MYOD, MYOG, MuRF‐1 and Atrogin‐1 in C2C12 myotubes in siNC and siNEU2 groups by qRT‐PCR (*n* = 3). (C) The protein levels (left) and statistical results (right) of MyoD, MyoG, MuRF‐1 and Atrogin‐1 in C2C12 myotubes in siNC and siNEU2 groups by Western Blot (*n* = 3). (D) Representative images of MyHC staining of C2C12 myotubes in siNC and siNeu2 groups (left) and statistical results of diameters of myotubes (right) by immunofluorescence (*n* = 8). Values are represented as means ± SEM. Exact *p* values are shown.
**Figure S7:** Effects of ANXA2 on neuron activation in DMS, M1 and M2. Related to Figure 6. (A, B) Representative images (A) and numbers (B) of c‐Fos positive cells in DMS of mice in Vehicle (Veh), AR test (Veh + AR), rA injection and AR test (rA + AR) groups (*n* = 3). (C, D) Representative images (C) and numbers (D) of c‐Fos positive cells in M1 of mice in Veh, Veh + AR and rA + AR groups (*n* = 3). (E, F) Representative images (E) and numbers (F) of c‐Fos positive cells in M2 of mice in Veh, Veh + AR and rA + AR groups (*n* = 3). (G) Fluorescence of cerebellum after AAV9‐L7‐6‐hM3D(Gq)‐mCitrine infection. Values are represented as means ± SEM. Exact *p* values are shown. DMS represents dorsomedial striatum. M1 represents primary motor cortex. M2 represents secondary motor cortex.
**Figure S8:** Effects of various antagonists on motor coordination in mice. Related to Figure 7. (A) Effects of AM630 systemic injection on AR latency to fall (*n* = 6). (B) Effects of AM251 systemic injection on AR latency to fall (*n* = 6). (C) Effects of Tranilast (Trani) systemic injection on AR latency to fall (*n* = 6). (D) Effects of Strychnine (Stry) systemic injection on AR latency to fall (*n* = 6). (E) The RNA and protein level in cerebellums of mice in AAV‐NC and AAV‐shCB2R groups. (F) AR latency to fall of mice in Vehicle (Veh), AAV‐shNC infection and rA injection (rA + AAV‐shNC), AAV‐shCB2R infection and rA injection (rA + AAV‐shCB2R) groups (*n* = 6). Values are represented as means ± SEM. Exact *p* values are shown.
**Figure S9:** Effects of isoliquiritigenin (ISL) on motor coordination and muscle function of aged mice. Related to Figure 8. (A) Molecular structure of ISL. (B) The RNA (left) and protein (middle) level and statistical results (right) of ANXA2 in C2C12 myotubes in control (C) group, Dexamethasone (Dex) group, Dex and 0.5 ng/mL ISL (Dex + LISL) group and Dex and 1 ng/mL ISL (Dex + HISL) group by qRT‐PCR and Western Blot (*n* = 3–4). (C) The body mass of mice during ISL gavage in CMC and CMC + ISL groups (*n* = 9–10). (D) The mass of tibialis anterior, gastrocnemius and quadriceps femoris of mice in CMC and CMC + ISL groups (*n* = 9–10). (E) Running time of mice in CMC and CMC + ISL groups on 12 mm (left) and 6 mm (right) balance beam tests (*n* = 10). (F) The average speed (left) and cadence (right) of mice in CMC and CMC + ISL groups in gait analysis (*n* = 10). (G) The support proportion of mice in CMC and CMC + ISL groups in gait analysis (*n* = 6). (H) The ANXA2 expression in cerebellums of mice in CMC and CMC + ISL groups by immunofluorescence. (I) The ANXA2 level of serum in mice in CMC and CMC + ISL groups by ELISA (*n* = 8). (J) Representative images of open field test and time in centre zone, four corners and four sides, total distance, centre distance, distance in four corners, four sides of mice in CMC and CMC + ISL groups (*n* = 9–10). (K) Representative images of elevated plus maze and time in open arms, centre and closed arms of mice in CMC and CMC + ISL groups (*n* = 9–10). (L) Food intake of mice in CMC and CMC + ISL groups (*n* = 7). Values are represented as means ± SEM. Exact *p* values are shown. CMC represents 0.5% sodium carboxyl methyl cellulose (CMC‐Na). CMC + ISL represents 20 mg/kg ISL (in 0.5% CMC‐Na).
**Figure S10:** HE staining of heart, liver, kidney, spleen and lung of mice in CMC and CMC + ISL groups. Related to Figure 8.

**Table S1:** The ANXA2 level in cerebellums of mice after saline and rA i.p. injection (ng/mL).
**Table S2:** Sequences of primers for qRT‐PCR.
**Table S3:** Sequences of shRNA for ANXA2 and CB2R.
**Table S4:** Sequences of siRNA for ANXA2 and Neu2.

## Data Availability

The mass spectrometry proteomics data have been deposited to the ProteomeXchange Consortium via the iProX partner repository with the dataset identifier PXD068704. The data that support the results of this study are available from the corresponding author upon reasonable request.
