# Supplementary material for: Annexin A2 Causes Motor Incoordination via Muscle–Cerebellum Axis in Sarcopenia
Source: J Cachexia Sarcopenia Muscle. 2026 Jan 26;17(1):e70203. doi: 10.1002/jcsm.70203 (PMC12835187; doi:10.1002/jcsm.70203)
Supplement: Supplementary file 1 — Data S1: Supporting information. [file JCSM-17-e70203-s001.docx]

Supplementary Methods

**Animal Experiments**

Mice aged 3 months (young) or 20 months (aged) were used to investigate motor coordination and muscle strength. Following a one-week adaptation period, a series of behavioral tests assessing motor coordination were performed. Upon completion of the behavioral assessments, tissues were collected for subsequent histological analysis.

For adeno-associated virus (AAV) injection into muscles, 3-month-old male mice were used following a one-week adaptation period. The mice were then maintained under specific pathogen-free (SPF) conditions. Living animal imaging was performed at the third week after AAV injection and the behavior tests were conducted at the fourth week. Then, the mice were sacrificed at the fifth week for histological analysis.

For AAV injection on cerebellums, 3-month-old male mice were used after one-week adaption stage. AAV was administrated into cerebellum via stereotactic injection. Then, the mice were maintained for one month, after which behavior tests were carried out, followed by sacrifice for tissue collection.

For experiments aimed at screening receptor targets, 3-month-old mice were used. Finishing one-week adaption, the mice were trained for two consecutive days on the rotarod test. On the third day, the mice received intraperitoneal injections of rA and antagonists, after which the rotarod test were performed.

For isoliquiritigenin (ISL) administration by gavage, ISL (Targetmol, USA) was dissolved in 0.5% (w/v) sodium carboxyl methyl cellulose (CMC-Na, Targetmol, USA). The 20-month-old male C57BL/6J mice were treated with 20mg/kg ISL or equivalent-volume CMC-Na once per day. The treatment was continued for 8 weeks, and body weights were recorded every two weeks. After finishing gavage, the behavior tests were conducted and mice were sacrificed for tissue collection. Histological analysis was performed afterwards.

**Behavior Tests**

**(1)** **Accelerated Rotarod Test.** A rotarod system (YLS-4D, Yiyan Technology Development Co., Ltd., Jinan, China) was used to evaluate motor coordination of young and aged mice. Young and aged mice were trained for 2 consecutive days (three times with 5 min intervals; 12, 14, 16 rpm for the first day; 18, 20, 22 rpm for the second day). On day 3, testing was performed using an accelerating protocol in which the speed increased from 4 to 40 rpm. Each mouse underwent three test trials with at least 5-min inter-trial intervals. The latency to fall or to cling and rotate passively with the rod was recorded ^1^. If a mouse remained on the rod for 300 s, the trial was terminated, and a latency of 300 s was assigned. After each fall, mice were immediately returned to their home cages. Between the trials, the device was cleaned with 75% ethanol (v/v) and dried to eliminate the smell of the previous mice.

**(2)** **Balance Beam Test.** The protocol of balance beam test was similar to the previous study^2^. The Balance beams (1 m in length, 6 mm or 12 mm in width; FLS-BBT, Future Life Science (Shanghai) Co., Ltd., Shanghai, China) were used to assess the motor coordination of young and aged mice. The beams were positioned 80 cm above the ground with a dark box placed at one end. A safety harness was installed beneath the beams to catch mice that might fall. The latency to traverse the beam from the starting point to the dark box was recorded. This test lasted for four days. Training sessions were conducted three times each day, with 15-minute intervals, over the first three days. On day 4, the formal tests were performed and the time was recorded three times in total for average. The beams were cleaned with 75% ethanol (v/v) and dried to eliminate the smell of the previous mice between each test.

**(3) Pole Test.** The apparatus for pole test was purchased from Beijing Cinontech Co. Ltd, Beijing, China (POL5008M). The length of the pole was 45cm and the diameter was 1cm. After installation, the pole was wrapped with gauze to prevent the mice from slipping. The experiment process lasted for three days. During the first two days, mice underwent training consisting of three trials per day. For each trial, mice were placed at the top of the pole with their heads oriented upward, and the latency to turn downward and descend to the base of the pole was recorded. The final test was carried out at the third day. Likewise, the pole was cleaned with ethanol to dispel the influence of mice odor.

**(4) Gait Analysis.** Catwalk gait analysis system (Catwalk XT, Noldus Information Technology B.V, Netherlands) was utilized to capture gait patterns of mice. After pre-training (3 days) for uninterrupted walks, the mice were placed on the glass plate. When mice walked from one end to the other end, their footprints were captured by the high-speed camera under the walking platform. At least 6 step cycles were collected for each mouse. Then, the gait data were analyzed using the software (CatWalk XT 10.6).

**(5)** **Grip Strength.** The dynamometer (SH2000M, Beijing Cinontech Co. Ltd, Beijing, China) was used to measure the grip strength of forelimbs and four paws. Mice were placed on a metal grid, allowing them to grip it with forelimbs or all four paws. They were then gently pulled backward by the tail in parallel to the ground until they could no longer maintain their hold on the grid. Each mouse underwent three trials, and the average of these trials was used to determine the muscle grip strength of each mouse.

**(6)** **Hanging Grid Test.** In this assay, the inverted hanging time was measured using a 30 × 30cm grid with 2mm thick bars and 18mm mesh, mounted on a 55-cm-high frame. A thick cushion was placed 50cm below the grid to protect fallen mice. Each mouse was positioned at the center of the grid, which was then inverted so the mouse's head faced downward. The time taken for the mouse to fall was recorded as the hanging time. Each mouse underwent three trials, with intervals of over 30 minutes between tests, and the average hanging time was calculated.

**(7)** **Open Field Test.** Open field test (square box: 50 × 50 cm, height 40 cm) (BW-OFT, Shanghai Bio-will Co., Ltd., China) was used to measure the locomotor activity and anxiety-like behavior of mice. The mice were placed in the center of the open field and allowed to explore for 10 min freely. The time and distance in different areas of the open field was tracked by video-tracking system (ANYMAZE, V6.0). The parameters including time spent in the center, four corners, four sides and the distance were used to evaluate the anxiety of mice. Between each test, the apparatus was cleaned with 75% ethanol and dried to eliminate the smell of the previous mice.

**(8) Elevated Plus Maze.** Elevated Plus Maze (EPM) apparatus (BW-DSG, Shanghai Bio-will Co., Ltd., China) was used to measure the anxiety of mice. The apparatus consists of two open arms and two closed arms (with 15-cm wall) radiating out at 90-degree angles and was set at 50 cm height. The mice were placed in the center of the apparatus and allowed to explore freely for 5 min. The time spent in open arms, closed arms and center was measured using a digital camera and video-tracking system (ANYMAZE, V6.0).

**(9) Food Intake Analysis.** Male C57BL/6J (20 months old) were bred and housed under a SPF with a 12-hour dark/light cycle and ad libitum access to water and food. After oral gavage of ISL for 8 weeks, 24-hour food intake was monitored and recorded.

**Myogenic Induction, Dexamethasone Treatment and rA Treatment.** When the cell density reached around 70-80%, the medium was changed with the myogenic induction medium (DMEM supplemented with 2% Horse Serum (Gibco, USA) and 1% penicillin/streptomycin). Then, the myogenic induction medium was refreshed every day. On day 3-4 after differentiation, Dexamethasone (Dex, Sigma, USA) at the concentration of 50μM was added to induce the atrophy of myotubes formed by C2C12 cells. As for rA treatment, when the C2C12 myotubes were formed, rA with different concentrations was added into the myogenic induction medium to treat myotubes for 24 hours. For Dex and ISL treatment, when the C2C12 myotubes were formed, 50μM dexamethasone (Dex group), 50μM dexamethasone and 0.5ng/ml (Dex+LISL group) ISL or 50μM dexamethasone and 1ng/ml ISL (Dex+HISL group) were added into myogenic induction medium to treat myotubes for 24 hours.

**Protein Extraction and Western Blot.** For tissue protein extraction, the tissues were harvested and frozen immediately with liquid nitrogen. A small piece of the tissue was excised and homogenized in Radio Immunoprecipitation Assay (RIPA) lysis buffer (Beyotime, China) with protease and phosphatase inhibitor (Selleck, China), followed by centrifugation at a speed of 14000 RCF for 15 min. For cell protein extraction, the cells were washed with Phosphate Buffered Saline (PBS, Servicebio, China) three times, then lysed with RIPA buffer for 30min, followed by centrifugation. The supernatant was separated and mixed with SDS-PAGE sample loading buffer (5X) (Beyotime, China) and boiled for 5 min. The immunoblotting method was described in our previous study^3^. The used antibodies were as follows: Anxa2 (Proteintech, USA), β-Actin (Proteintech, USA), Gapdh (Cell Signaling Technology, USA), Atrogin-1 (Santa Cruz, USA), MuRF-1 (Santa Cruz, USA), MyHC (Santa Cruz, USA), MyoD (Santa Cruz, USA), MyoG (Santa Cruz, USA), Flag (Cell Signaling Technology, USA), p53 (Cell Signaling Technology, USA), p21 (Abclonal, China).

**RNA Extraction and qRT-PCR.** For tissue, after collection, the tissues were stored in RNAlater (ThermoFisher Scientific, USA) at -80 ℃ until extraction. Likewise, the excised tissues were pestled in TRIzol (Invitrogen, USA), followed by centrifugation to abandon tissue sedimentation. For cell, after washing with PBS, the cells were treated with TRIzol for 10min. The subsequent methods were identical to our previous study^4^. For qRT-PCR, the PrimeScript Master Mix (TaKaRa, Japan), 2× SYBR Green qPCR Master Mix (Low ROX) (Selleck, China) and Applied Biosystems 7500 Real-Time PCR System (Applied Biosystems, USA) were used according to the manufacturers’ instructions. Glyceraldehyde- 3-phosphate dehydrogenase (GAPDH) was used as the quantitative control for normalization. The 2−ΔΔCt method was used to calculate the relative mRNA levels. The primers used in this study are listed in **Table S2**.

**ELISA.** Mouse ANXA2 Elisa Kit and Human ANXA2 Elisa Kit were both purchased from MULTI SCIENCES. According to the instructions, the samples (cell supernatant or serum) were introduced into the designated wells (50μl/well) and mixed with 50μl biotin-labeled antigen solution. After incubation at 37℃ for 30 min, the plate was washed with Wash Buffer five times. Next, 50μl/well horseradish peroxidase-conjugated streptavidin was added and incubated again. After additional wash, Chromogenic Agent A and B were added, followed by 10-minute incubation at 37℃ in the darkness. Then the reaction was stopped and the OD value was measured at 450 nm using Microplate reader (Infinite M200 Pro, Tecan, Switzerland). The ANXA2 concentration was calculated by the software ELISAcalc.

**Human Sample.** This collection was approved by Shanghai Ninth People’s Hospital, Shanghai Jiao Tong University School of Medicine Ethics Committee (SH9H-2023-T493-2). Patient blood and muscle samples were obtained from Shanghai Ninth People’s Hospital, Shanghai Jiao Tong University School of Medicine. The participants were divided into two group, namely young-H group (age < 30 years) and aged-H group (age > 65 years). The peripheral blood was extracted and stored at 4°C. Then, the serum was separated with a refrigerated centrifuge (1,000g for 10 min) and were stored at −80 °C. The expression of ANXA2 in serum was detected by ELISA. For muscle samples, the samples were fixed in 4% Paraformaldehyde (PFA, Biosharp, China) immediately for 24 hours. Then the samples were embedded and the expression of ANXA2 was detected by immunofluorescence.

**Histological Analysis.** For paraffin section of tissues except cerebellum, the muscles were collected after mice were sacrificed and weighed immediately. Then, the samples were fixed with 4% paraformaldehyde (PFA, Biosharp, China) for 24h and cut to smooth. Then the samples were dehydrated with gradient alcohol and embedded in paraffin. Next, the specimens were sectioned at a thickness of 4 μm. For frozen section of muscles, the muscle tissues were fixed in 4% PFA for 24h and immersed in 15% sucrose (Sinopharm, China) at 4 °C until they sank. Then the samples were transferred to 30% sucrose at 4°C until they sank, followed by embedded in OCT (Sakura, Japan) and frozen to white. Then, the samples were sectioned. For paraffin section of cerebellum, the mice were deeply anesthetized with isoflurane and were then transcardially perfused with PBS solution from apex cordis, which was followed with 20 mL 4% PFA. After the mice twitched and became stiff, brains combined with cerebellums were extracted and post-fixed in PFA at 4 °C overnight and dehydrated using gradient alcohol, followed by embedded in paraffin. Sections were cut with 4 μm thickness.

**Hematoxylin-eosin (HE) Staining.** The paraffin sections were dewaxed in Environmentally friendly dewaxing transparent liquid (Servicebio, China) and rinsed with tap water. Hematoxylin-eosin (H&E) HD constant dye kit (Servicebio, China) was used to perform HE staining. In brief, hematoxylin and eosin staining were conducted, followed by dehydration and sealing. Then, the images were captured by light microscope (Leica, USA).

**Nissl Staining.** Consistent with HE staining, the sections were dewaxed first. The tissue slices were immersed in dye solution (Servicebio, China) for 2-5 minutes, followed by slightly differentiation in 0.1% glacial acetic acid (Sinopharm, China). Then, the sections were treated with Xylene (Sinopharm, China) and sealed. Then, the images were captured by light microscope (Leica, USA).

**Immunofluorescence.** For paraffin section, the sections were dewaxed in the Xylene (Sinopharm, China) and immersed in gradient alcohol. After antigen retrieval, the sections were boiled in the microwave oven, followed by cooling to room temperature. Then the samples were covered with 3% H_2_O_2_ (Sinopharm, China) and incubated for 25 min away from light. After washing in PBS, the samples were blocked in 3% BSA for 30min. Following removal of the blocking solution, the sections were incubated in the primary antibodies at 4°C overnight and incubated with fluorescent-labeled secondary antibodies at room temperature for 1 hour. Next, the nuclei were stained with 4',6-diamidino-2-phenylindole (DAPI) for 10 min, and anti-fading mounting solution (BioMed World, China) was added. For frozen section, the slices were fixed in cold acetone for 10 min after rewarming. Then, antigen retrieval and BSA blocking were performed. Primary antibodies, secondary antibodies and DAPI were incubated successively. The following antibodies were used: Anxa2 (Proteintech, USA), Laminin (Sigma, USA), Calbindin (Santa Cruz, USA), GFAP (Santa Cruz, USA), MyHC (Santa Cruz, USA). DAPI was purchased from Beyotime, China.

**Cell Count Kit-8 (CCK8).** CCK-8 (Dojindo, Japan) was used to explore proliferation of C2C12 cells according to the instructions of the manufacturers. CCK8 was performed on day 1 to day 3 after adenovirus infection or recombinant ANXA2 (rA, MedChemExpress, USA) stimulation on C2C12 cells. The cells were incubated in complete medium with 10% CCK-8 reagent for 1 h at 37°C. The absorbance of the supernatant at 450 nm and 630nm was measured using a microplate reader (Infinite M200 Pro, Tecan, Switzerland).

**EdU Assay.** For EdU positive cell detection, we used BeyoClick™ EdU Cell Proliferation Kit with Alexa Fluor 488 (Beyotime, China). EdU assay was conducted on day 3 after adenovirus infection or rA stimulation. At the end of stimulation, the cells were incubated in the medium with EdU for 2 hours. Then, the cells were fixed with 4% paraformaldehyde and treated with 0.3% Triton-X 100 (Biofroxx, German). After removing Triton-X 100 and wash with PBS, the cells were incubated with Click Reaction Buffer, composed of CuSO4 , Azide 488, Click Additive Solution, at room temperature away from light. After staining cell nuclei with DAPI for 5 min, the fluorescence was captured with a fluorescence microscope (Leica, USA). The number of EdU positive cells was calculated by ImageJ.

**Senescence β-Galactosidase Staining.** To investigate the senescence of C2C12, senescence β-galactosidase staining (S-β-Gal, Beyotime, China) was carried out on day 3 after adenovirus infection or rA stimulation. The cells were fixed at room temperature for 15min and wash with PBS three times, followed by incubated in staining solution at 37℃ overnight. The images were captured using light microscopy on the second day.

**RNA Sequencing.** Total RNA was extracted using TRIzol, followed by evaluating RNA purity and quantification with NanoDrop 2000 spectrophotometer (Thermo Scientific, USA). RNA integrity was assessed using the Agilent 2100 Bioanalyzer (Agilent Technologies, Santa Clara, CA, USA). The transcriptome sequencing and analysis were conducted by OE Biotech Co., Ltd. (Shanghai, China).

**Living Animal Imaging.** At the third week after AAV intramuscular injection, living animal imaging (IVIS Spectrum Imaging System, PerkinElmer) was conducted to detect the infectious efficiency of AAV. The mice were anesthetized with isoflurane (RWD, China) and placed on the platform with the lateral side upward. The excitation wavelength was set at 480nm and the emission wavelength was set at 520nm. Then, the images were acquired and quantified using Living Image v2.5 software (PerkinElmer).

**General Drug Administration Procedure.** For experiments evaluating intraperitoneal injection of rA on motor coordination, the ANXA2 level in serum reached peak at 2 hours after intraperitoneal injection, according to previous study^5^. In our study, 3-month-old mice were pre-trained for two days using the same protocol described in **Accelerated Rotarod Test** part. On the third day, 1μg/g rA dissolved in PBS was injected intraperitoneally. Two hours later, AR test was performed. Upon completion of the test, mice were sacrificed within 1.5h for cerebellum collection and c-Fos detection.

For experiments screening the receptor targets about ANXA2, various types of receptor antagonists were utilized. 3-month-old mice were trained for two days in advance. rA (0.5ug/g) was injected into 4/5Cb 2 hours before the tests on the 3^rd^ day. Then, Tranilast (300 mg/kg) against TRPV2, strychnine (1.0 mg/kg) against GlyR, AM251 (5.0 mg/kg) and AM630 (5.0 mg/kg) respectively against CB1R and CB2R were i.p. injected 20 min before tests.

**Surgery and Stereotactic Injection.** The male C57BL/6J were anesthetized with isoflurane and then mounted on a stereotaxic frame (RMD Life Science). Following disinfection, a scalp incision was made along the brain midline, and the craniotomies was performed using a high-speed drill at the stereotaxic coordinates (in mm from bregma): anteroposterior (AP), -5.80; later (LAT), 0.00; ventral (V), -1.25 from the dural surface. The skull was leveled by placing bregma and lambda in the same horizontal plane. Then the injection was performed. Antibiotics were administered to reduce the risk of infection. The body temperature of the mice was kept using a heating pad throughout the procedure.

For experiments involving local rA injection, a small hole was drilled at the previously described coordinates with a needle of 1ml syringe. rA (0.5μg/g) was injected into cerebellum using microsyringe, with the needle retaining in the cerebellum for 10 min to allow for diffusion of drugs. Then the microsyringe was pulled out gently. The skull was disinfected with odophor and the incision was sutured. The mice were immediately transported back to their home cages following the infusion process. After 2 hours, the behavioral tests were carried out.

For experiments involving local AM630 and rA injection, AM630 was dissolved in DMSO:saline solution (2:1). 3μg AM630 was injected into the aforementioned coordinates. Thirty minutes later, rA (0.5μg/g) was injected into the same location. Then, accelerated rotarod tests were performed at 1.5 hours after rA injection.

For experiments involving intracranial virus injection and manipulation, 300nL AAV9-L7-6-hM3D(Gq)-mCitrine (2.38E13 VG/ml) and AAV9-L7-6-mCitrine (1.31E13 VG/ml) was injected into cerebellum at the previously described coordinates. Post operational anti-infective treatments lasted for 3 days. Then the mice were kept under the condition of 12-hour dark–light cycle at 22 °C for 3 weeks and used for behavioral tests. rA was intra-Cb injected 2 hours before the rotarod tests. Next, mice received i.p. injections of 1.0 mg/kg CNO in sterile saline 20 min before the rotarod tests. After behavioral experiments, mice were perfused and the brains were collected and processed for mCitrine staining.

**Immunohistochemistry.** The paraffin sections were dewaxed and antigen retrieval was conducted. Then the sections were treated with 3% H_2_O_2_ for 25min. Then, the slices were blocked and incubated with c-Fos (Servicebio, China) primary antibody and Horseradish Peroxidase (HRP)-labeled secondary antibody (Servicebio, China). Diaminobenzidine (DAB) staining was carried out and the staining time was controlled under microscopy.

**References**

1. Eltokhi A, Kurpiers B, Pitzer C. Comprehensive characterization of motor and coordination functions in three adolescent wild-type mouse strains. *Sci Rep* 2021;**11**:6497.

2. Ji YR, Tona Y, Wafa T, Christman ME, Tourney ED, Jiang T *et al.* Function of bidirectional sensitivity in the otolith organs established by transcription factor Emx2. *Nat Commun* 2022;**13**:6330.

3. Jiao X, Zhang Y, Li W, Zhou X, Chu W, Li Y *et al.* HIF-1α inhibition attenuates severity of Achilles tendinopathy by blocking NF-κB and MAPK pathways. *Int Immunopharmacol* 2022;**106**:108543.

4. Jiao X, Wang Z, Li Y, Wang T, Xu C, Zhou X *et al.* Fullerenol inhibits tendinopathy by alleviating inflammation. *Front Bioeng Biotechnol* 2023;**11**:1171360.

5. Cheng C, Wang X, Jiang Y, Li Y, Liao Z, Li W *et al.* Recombinant Annexin A2 Administration Improves Neurological Outcomes After Traumatic Brain Injury in Mice. *Front Pharmacol* 2021;**12**:708469.
