# Supplementary material for: Annexin A2 Causes Motor Incoordination via Muscle–Cerebellum Axis in Sarcopenia
Source: J Cachexia Sarcopenia Muscle. 2026 Jan 26;17(1):e70203. doi: 10.1002/jcsm.70203 (PMC12835187; doi:10.1002/jcsm.70203)
Supplement: Supplementary file 2 — Data S2: Supporting information. [file JCSM-17-e70203-s004.pdf]

Fig 2C TA Anxa2

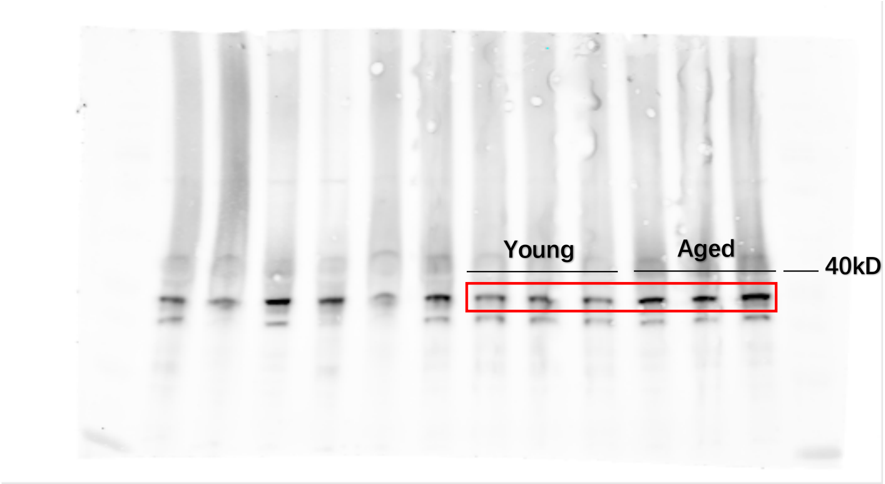

Fig 2C TA β-Actin

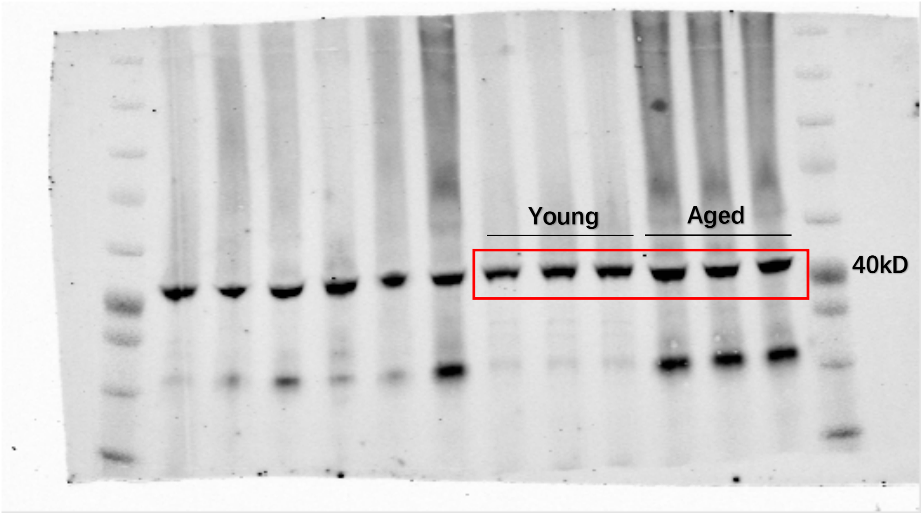

Fig 2C G Anxa2

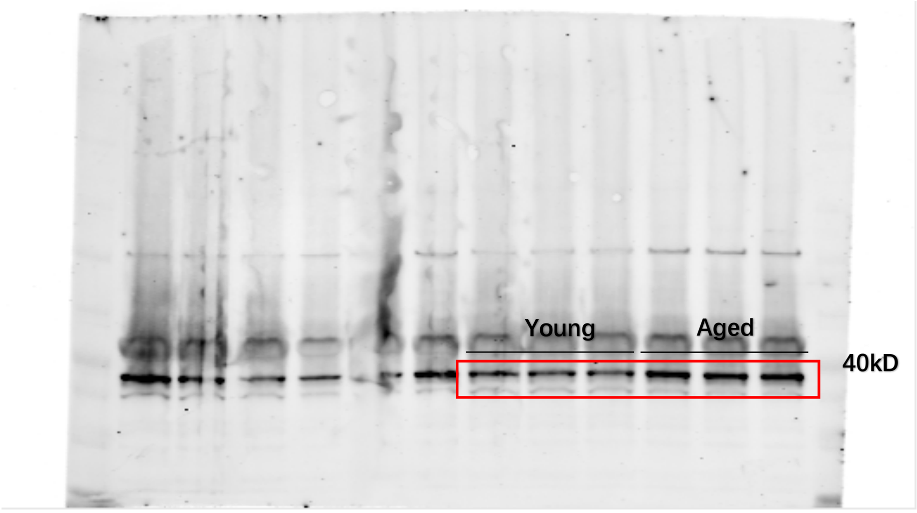

Fig 2C G β-Actin

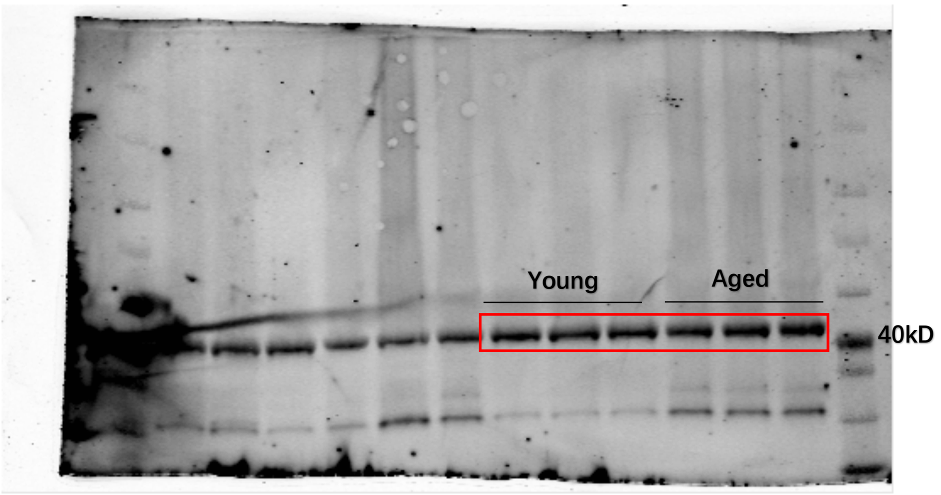

Fig 2C Q Anxa2

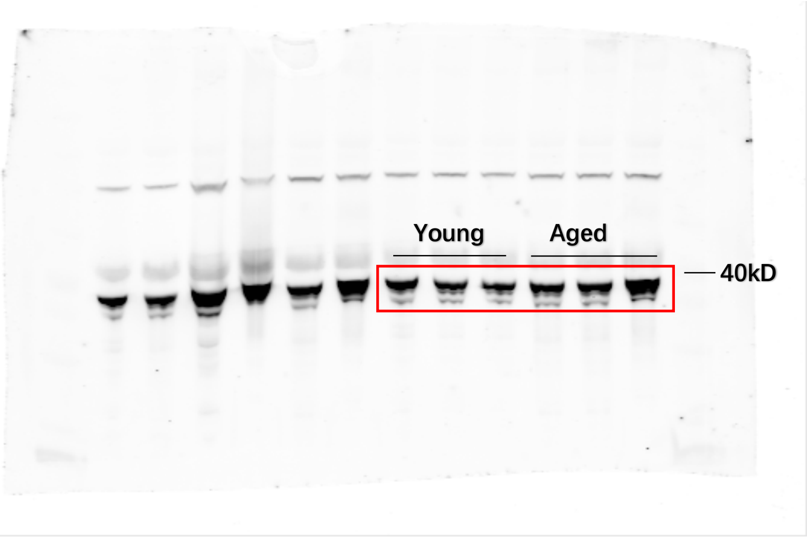

Fig 2C Q β-Actin

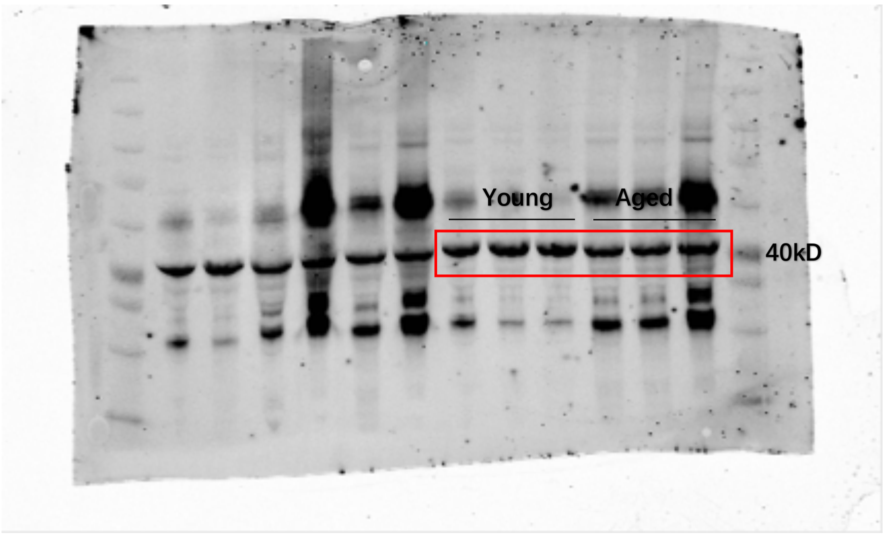

Fig 2C SOL Anxa2

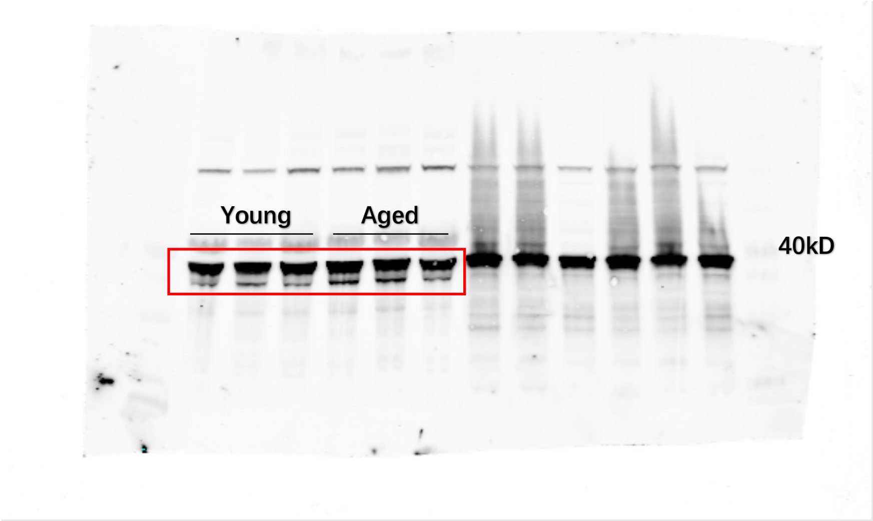

Fig 2C SOL β-Actin

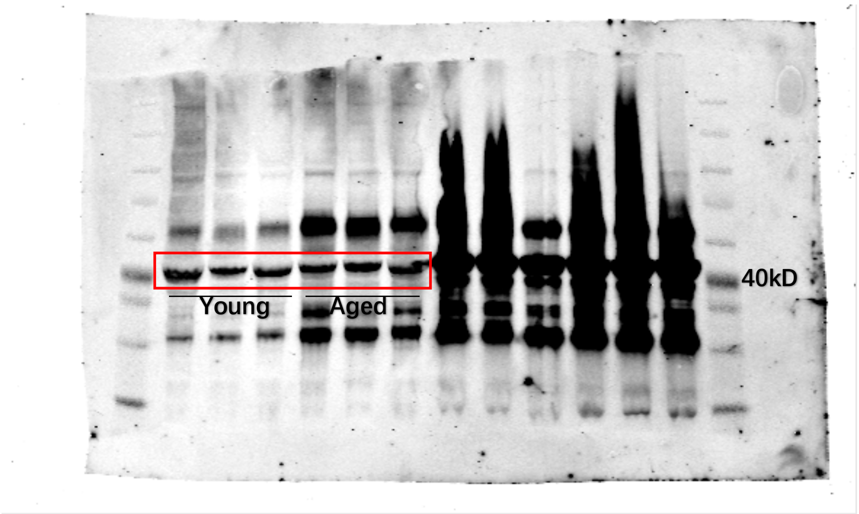

Fig 2E Anxa2

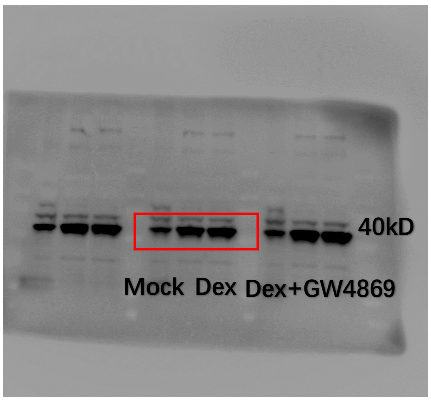

Fig 2E β-Actin

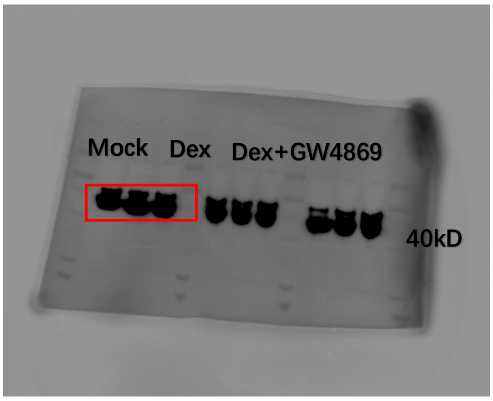

Fig 2F Anxa2

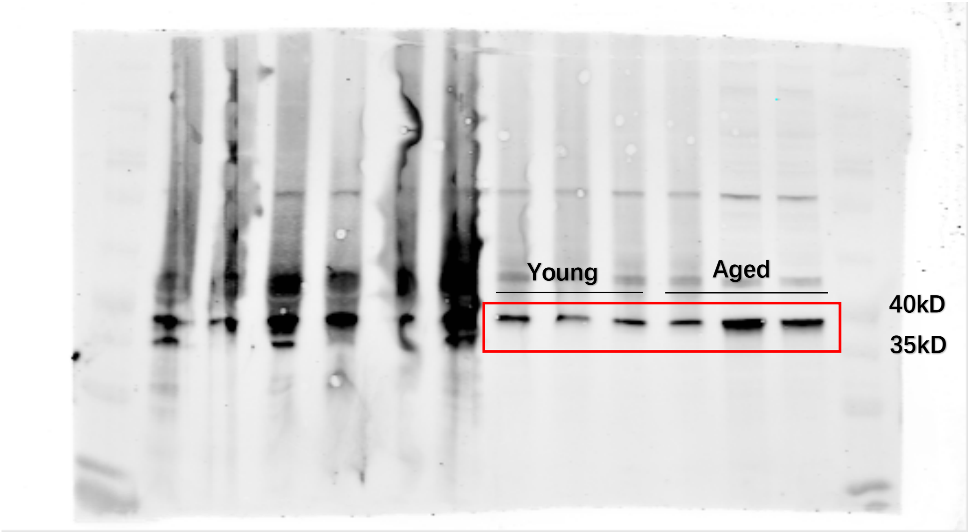

Fig 2F β-Actin

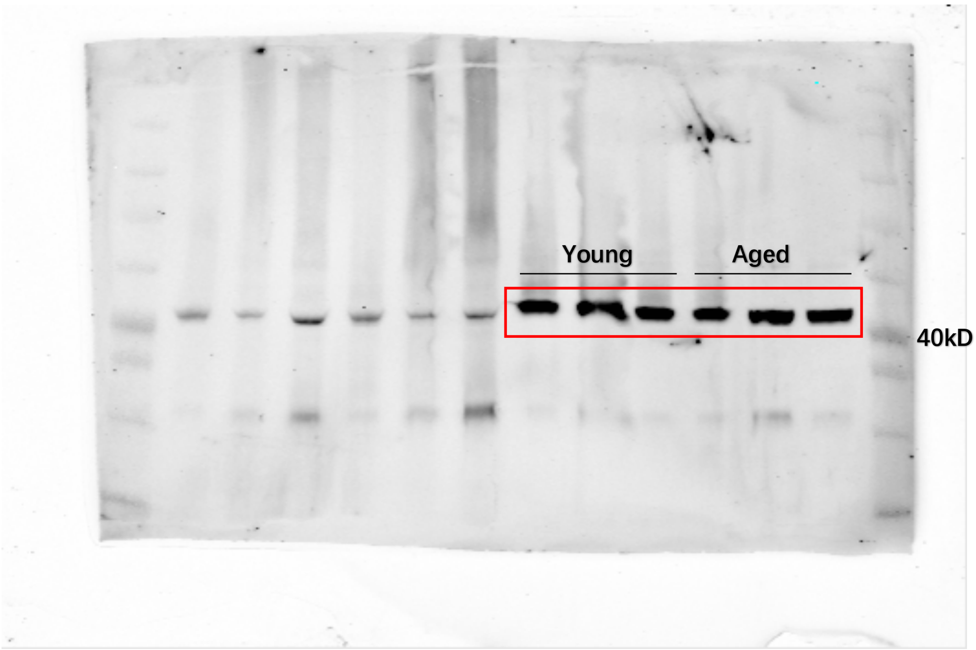

Fig 3D p21

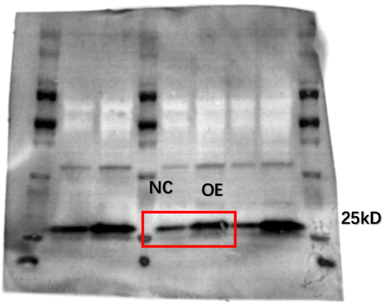

Fig 3D p53

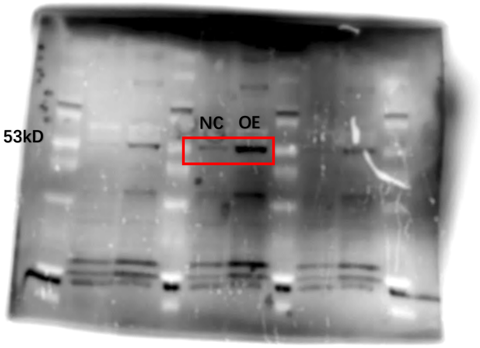

Fig 3D actin

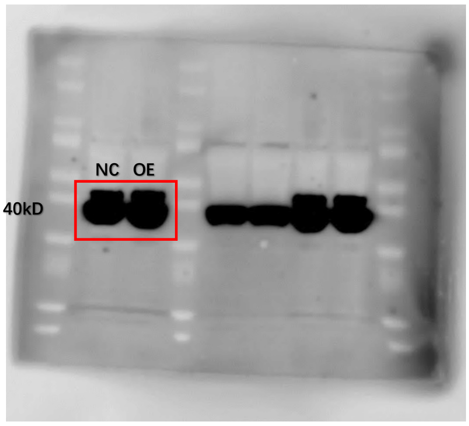

Fig 3F MyHC

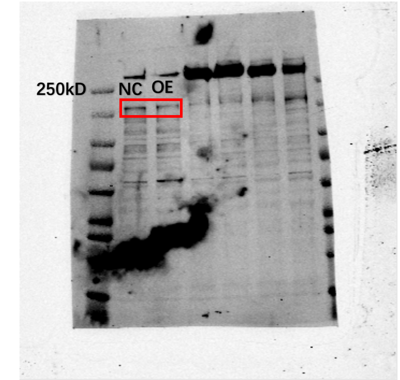

Fig 3F MyoD

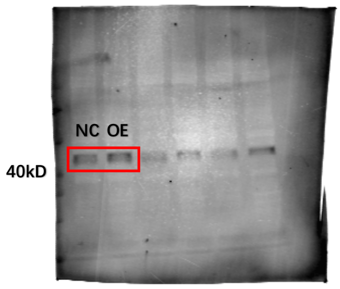

Fig 3F MyoG

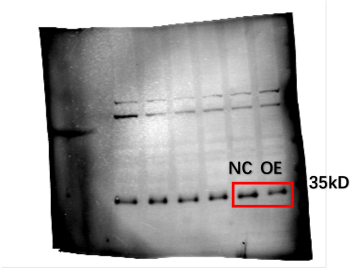

Fig 3F Atrogin-1

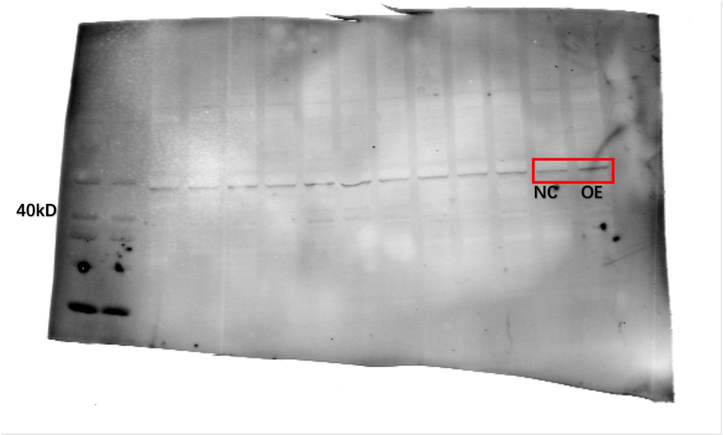

Fig 3F MuRF-1

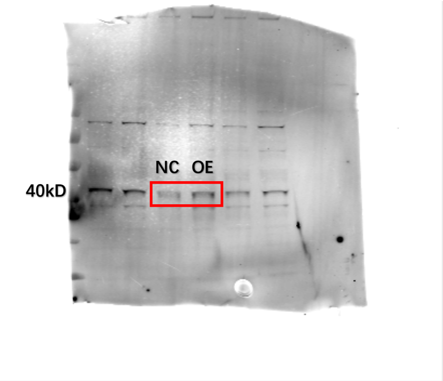

Fig 3F Gapdh

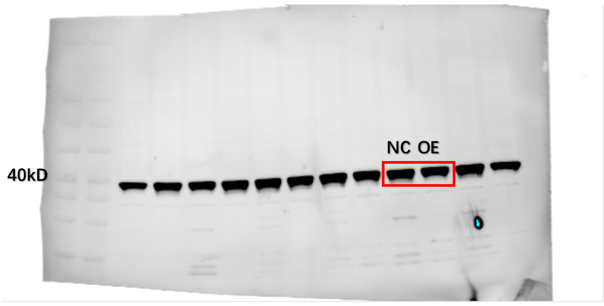

Fig 4F MyHC

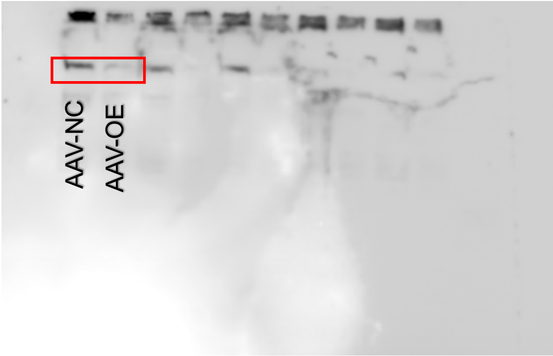

sample

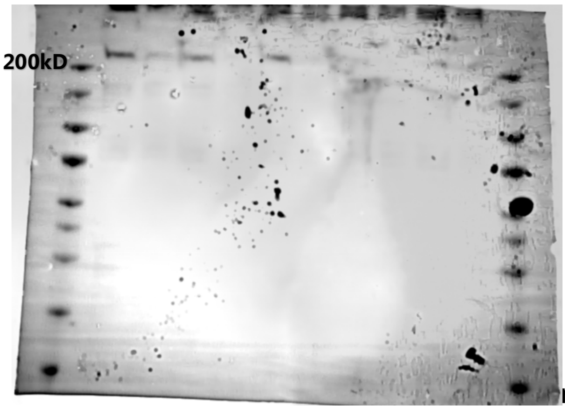

merge

Fig 4F Atrogin-1

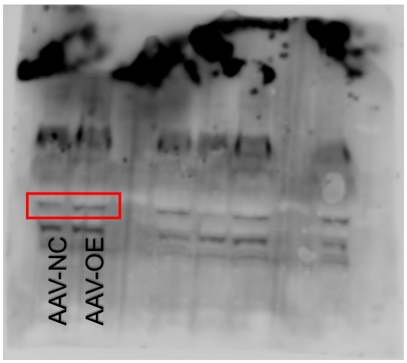

sample

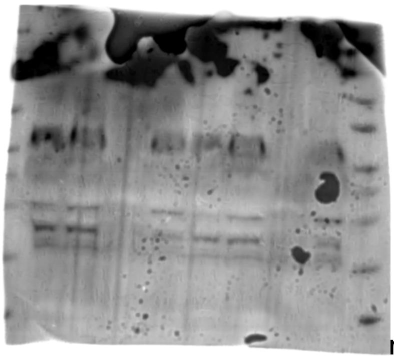

merge

Fig 4F MuRF-1

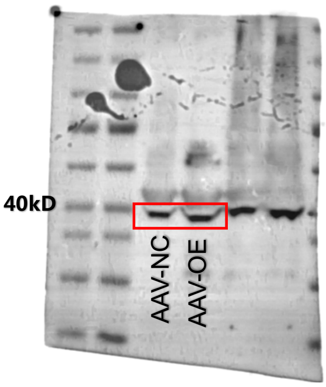

Fig 4F Gapdh

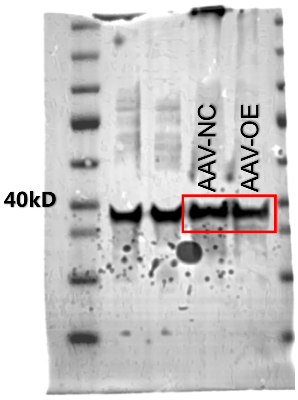

Fig5B Neu2

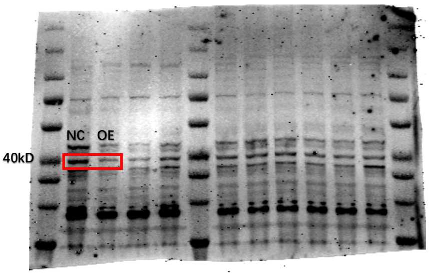

Fig5B Gapdh

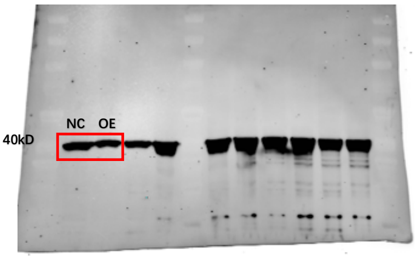

Fig 5D Anxa2

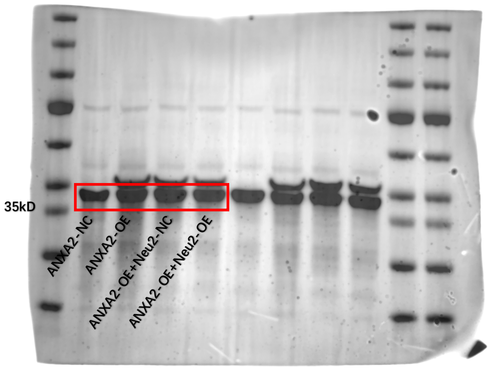

Fig 5D Neu2

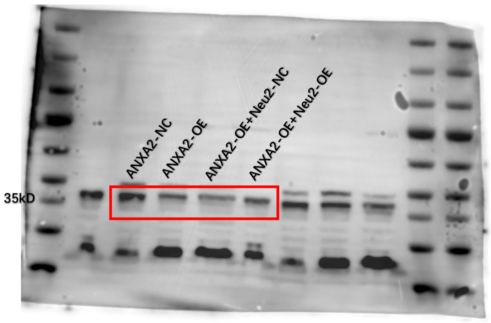

Fig 5D MyoD

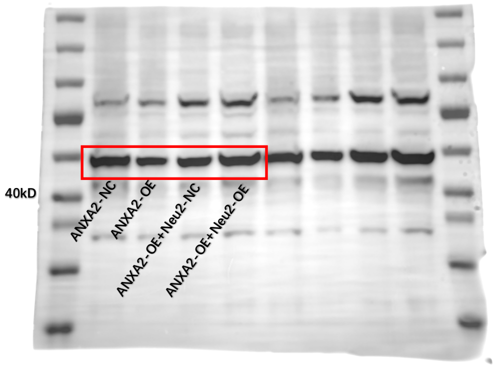

Fig 5D MyoG

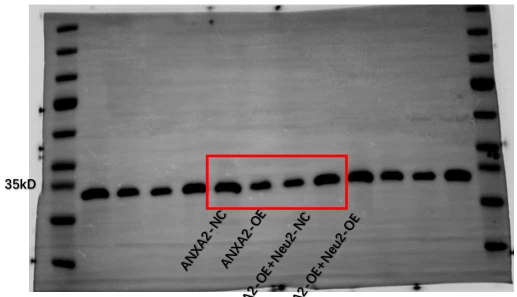

Fig 5D MuRF-1

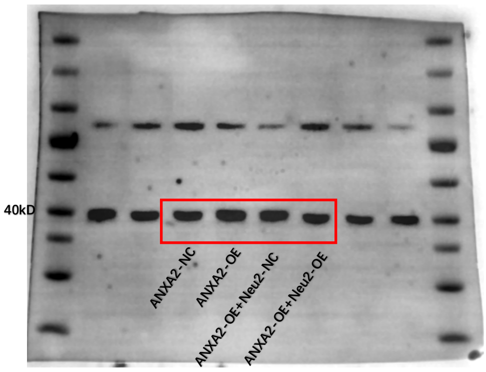

Fig 5D Atrogin-1

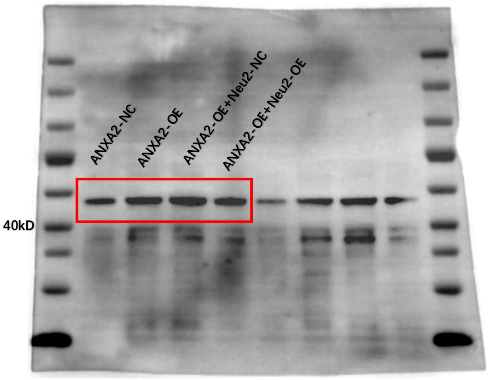

Fig 5D Gapdh

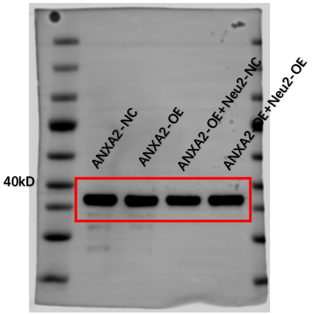

Fig 8D MyHC

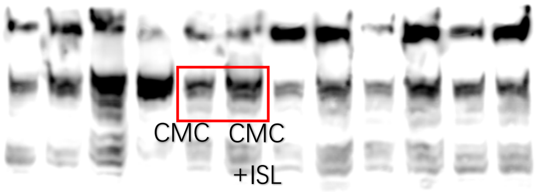

sample

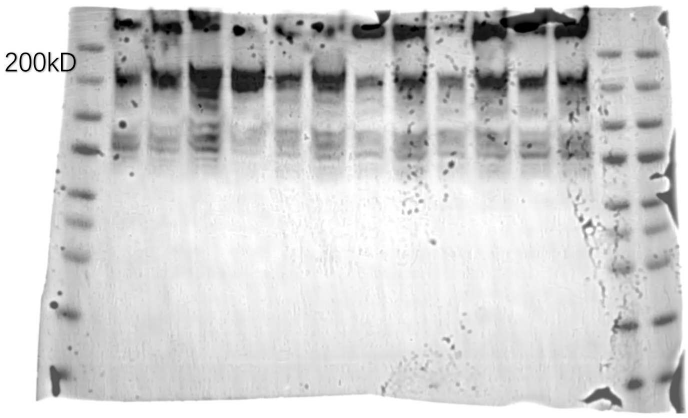

merge

Fig 8D MuRF-1

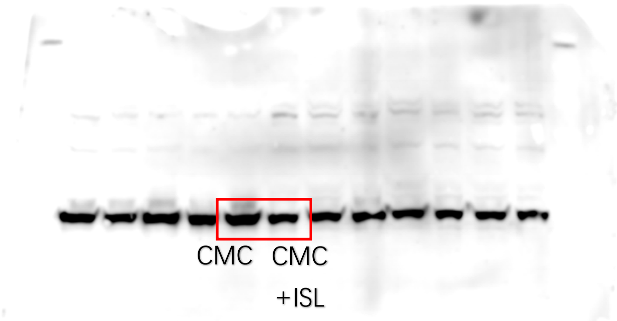

sample

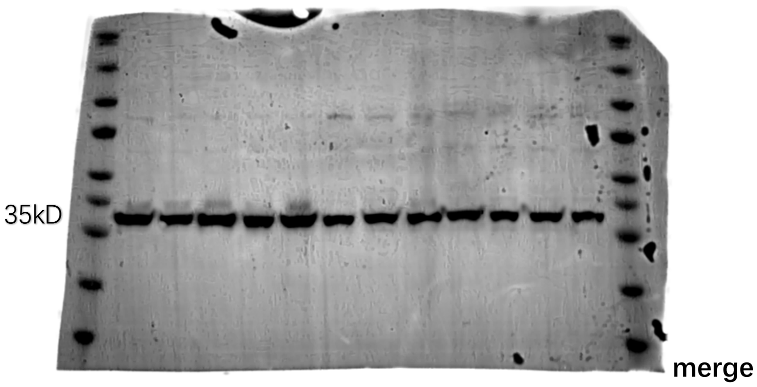

Fig 8D Atrogin-1

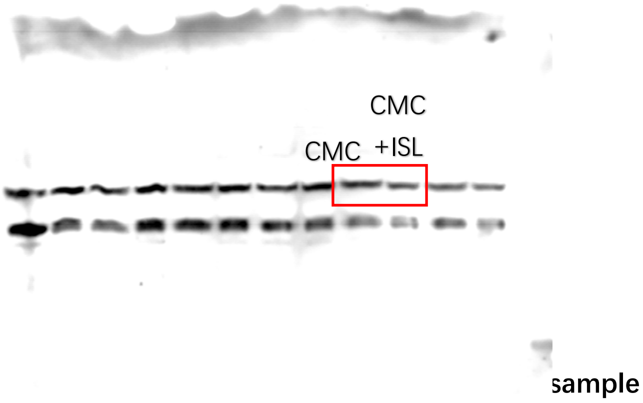

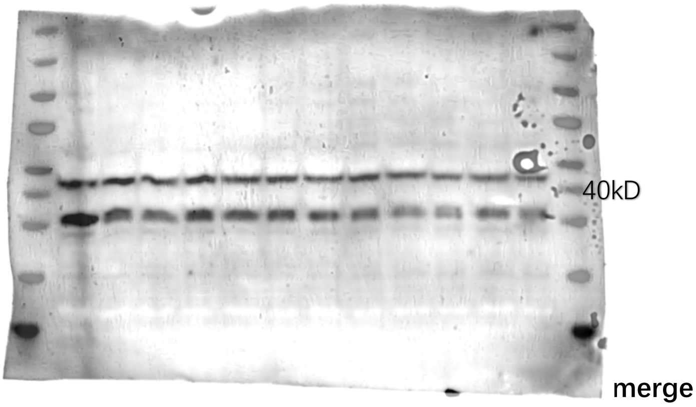

Fig 8D Gapdh

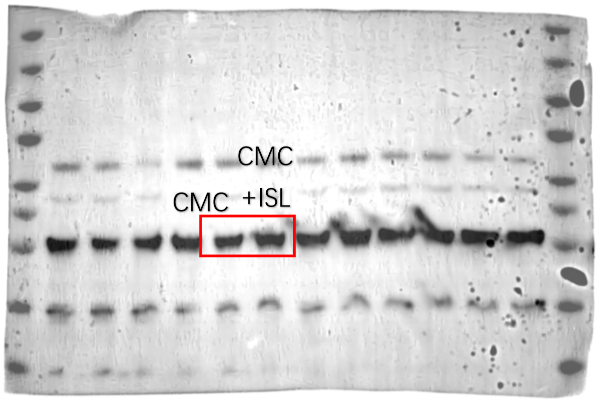

Fig S2D Liver Anxa2

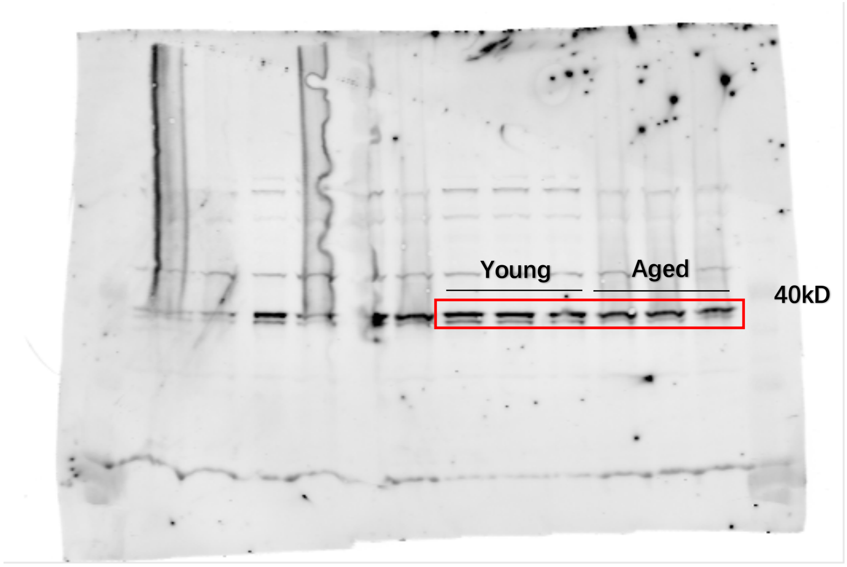

Fig S2D Liver β-Actin

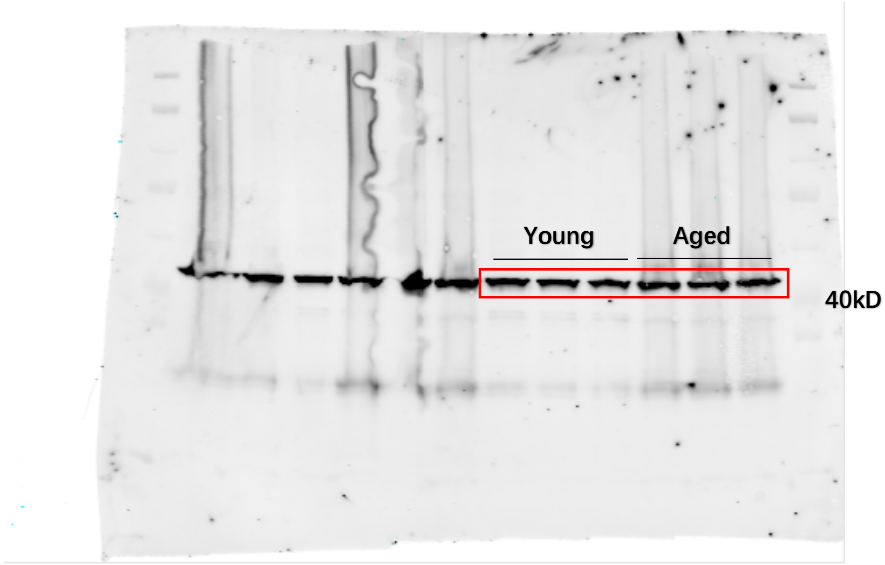

Fig S2D Lung Anxa2

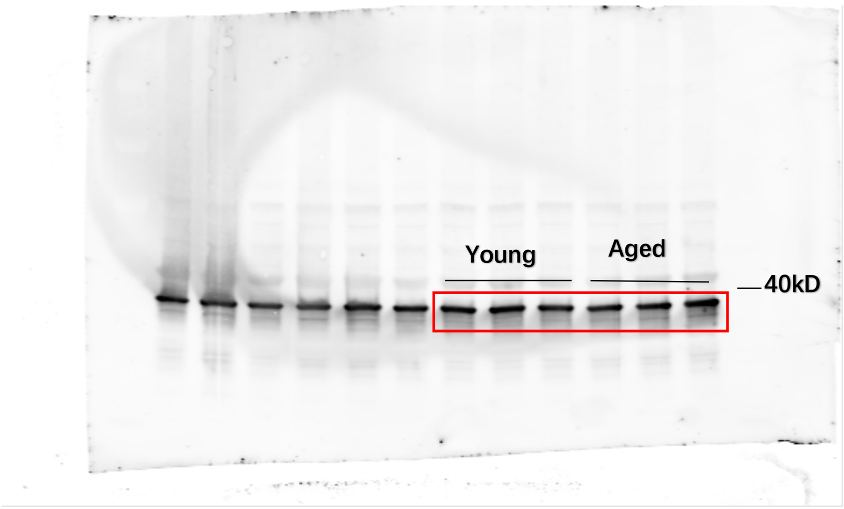

Fig S2D Lung β-Actin

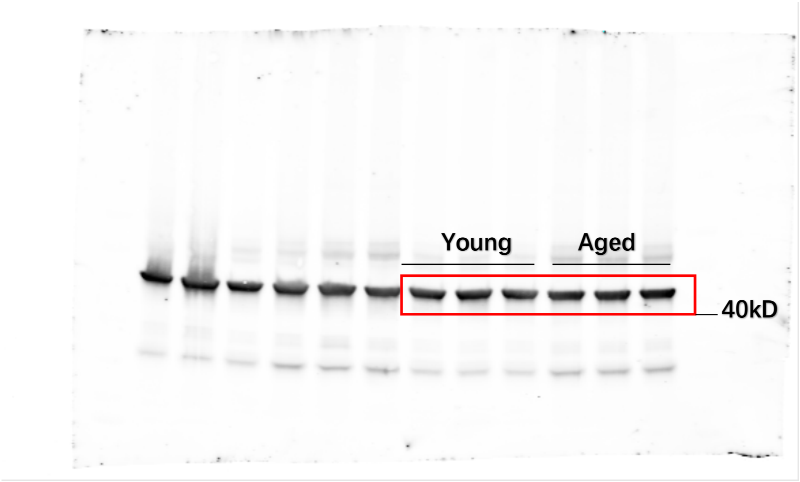

Fig S2D Kidney Anxa2

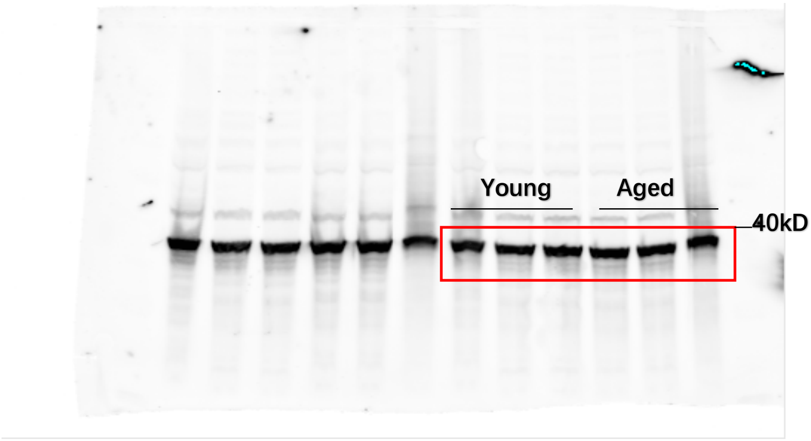

Fig S2D Kidney β-Actin

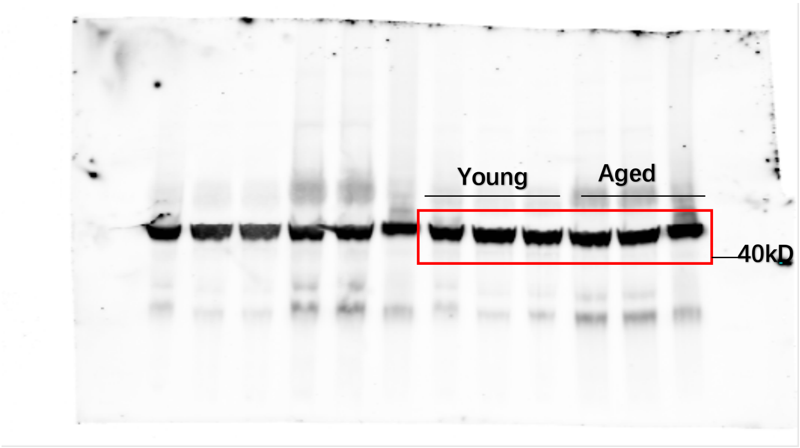

Fig S2D Spleen Anxa2

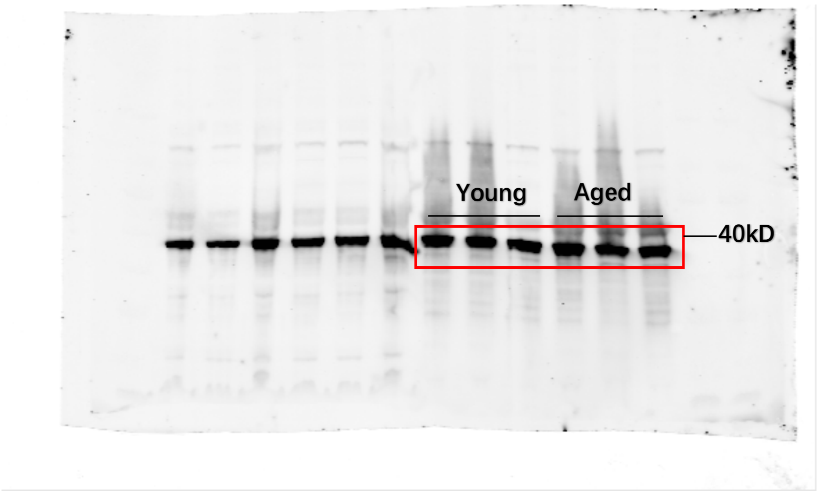

Fig S2D Spleen β-Actin

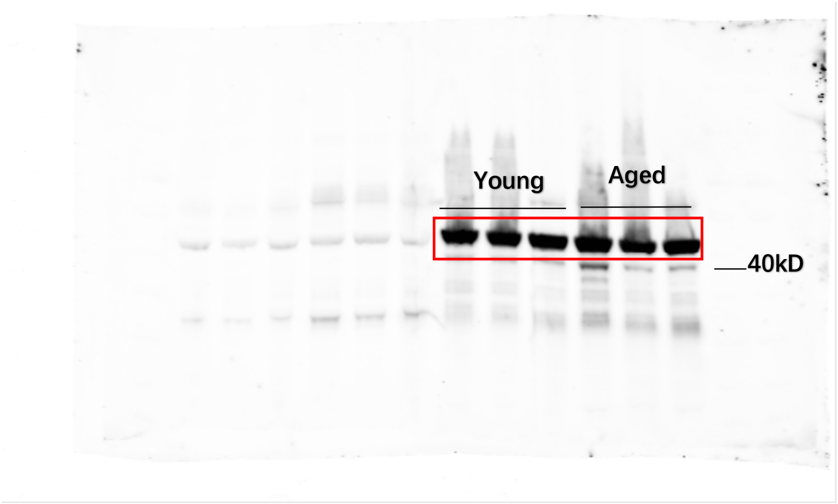

Fig S2D Heart Anxa2

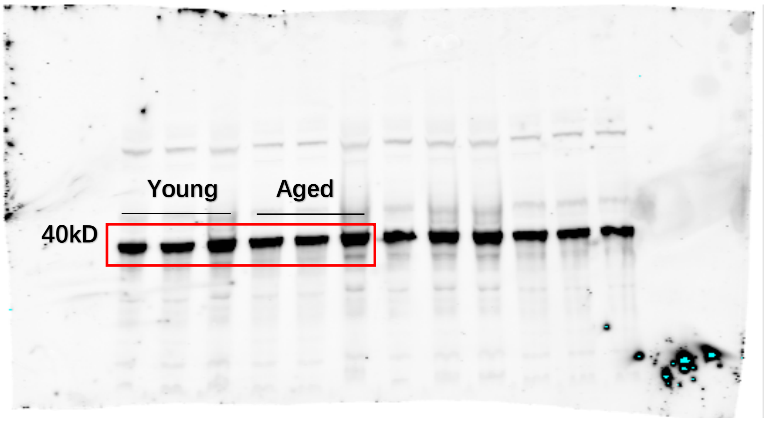

Fig S2D Heart β-Actin

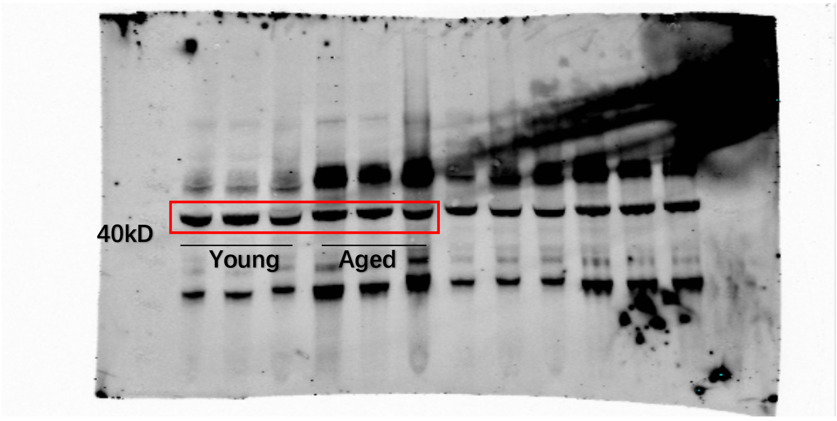

Fig S2H Atrogin-1

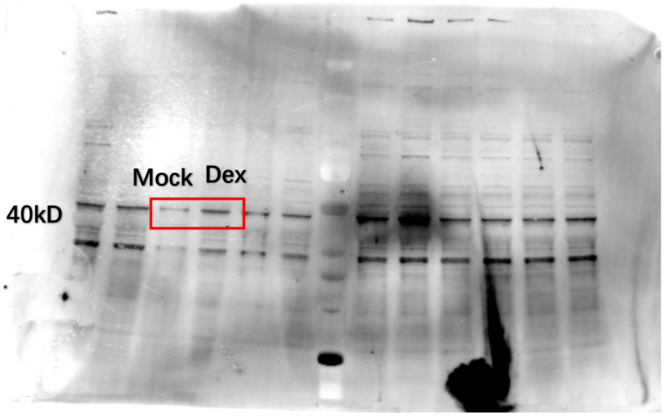

Fig S2H MuRF-1

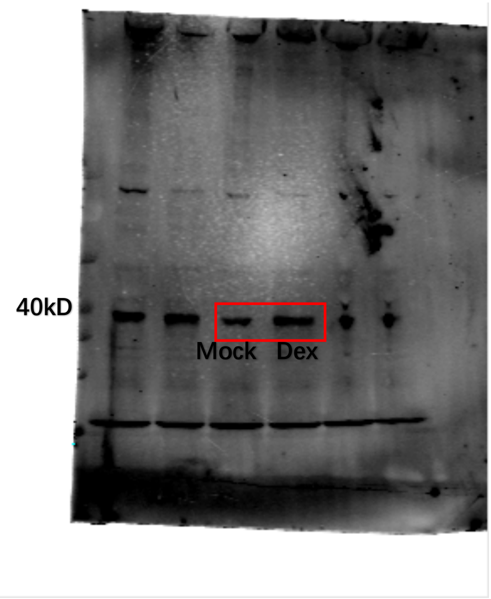

Fig S2H MyHC

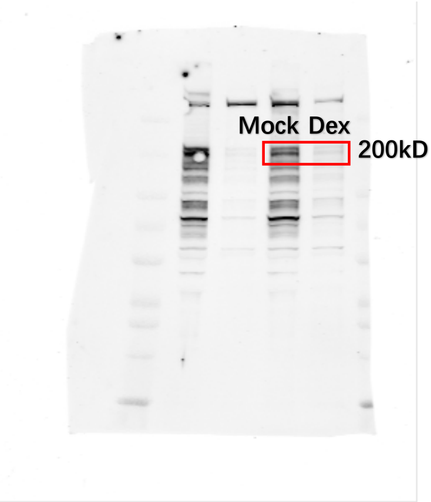

Fig S2H MyoD

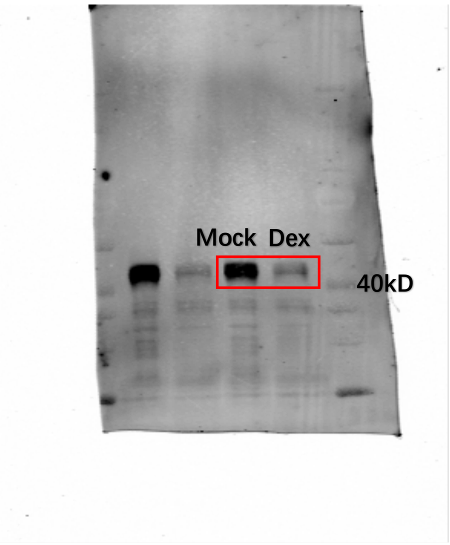

Fig S2H MyoG

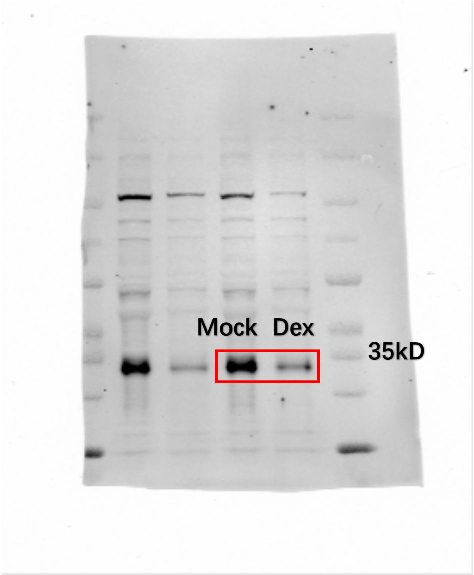

Fig S2H Gapdh

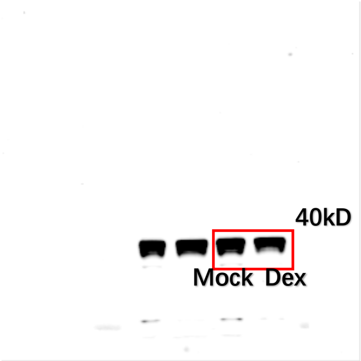

Fig S3A Flag

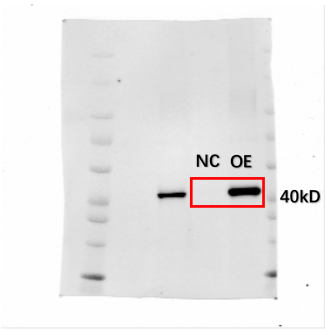

Fig S3A GAPDH

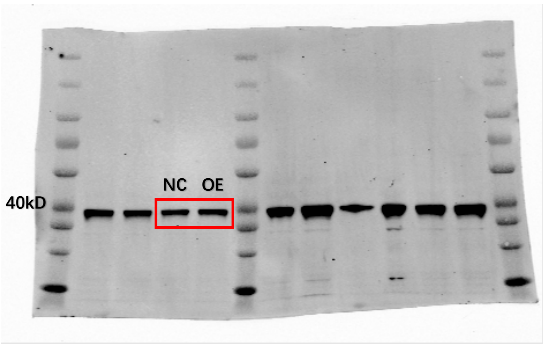

Fig S3C Anxa2

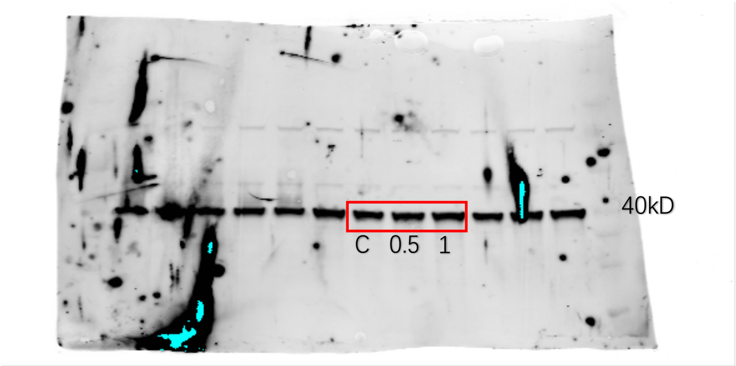

Fig S3C β-Actin

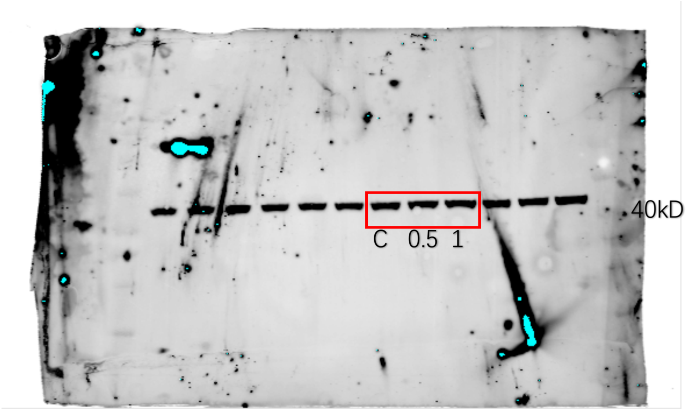

Fig S3E p21

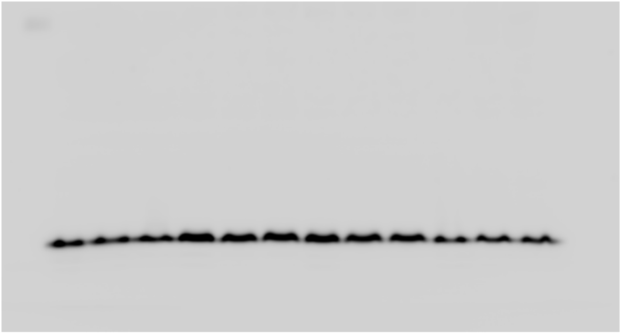

sample

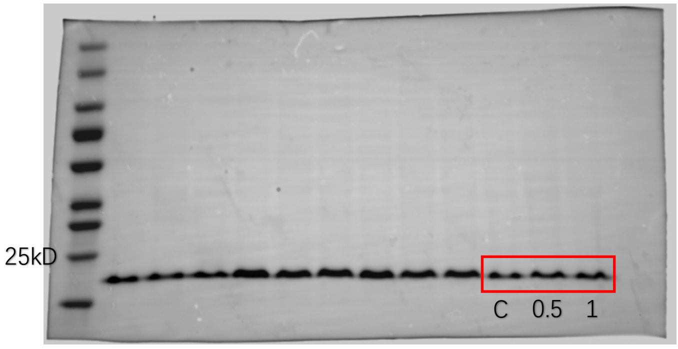

Merge

Fig S3E p53

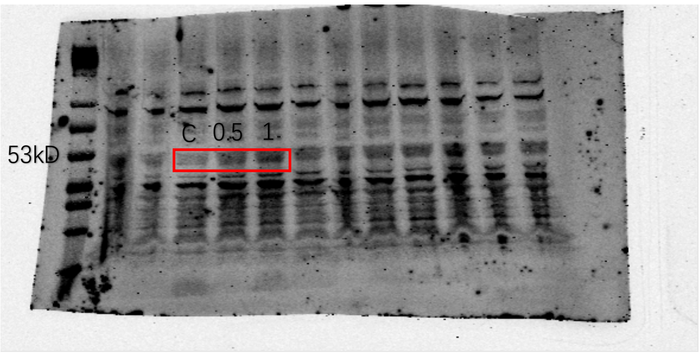

Fig S3E Gapdh

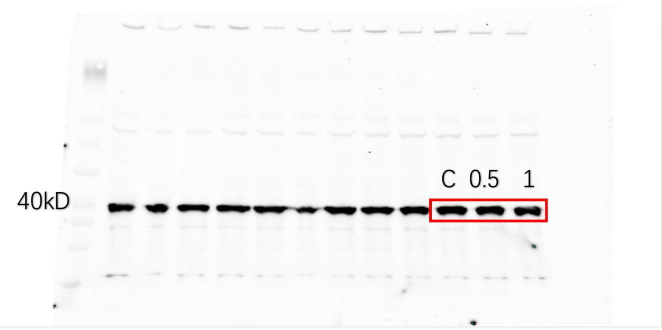

Fig S3J MyHC

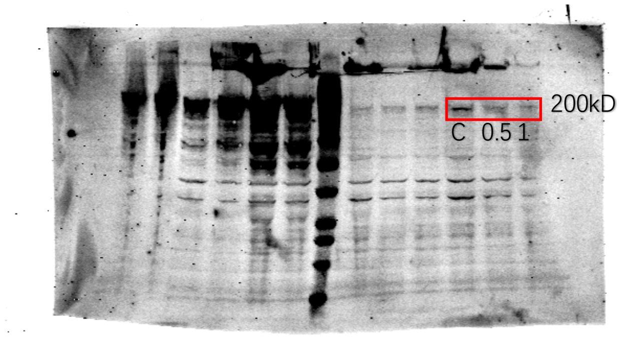

Fig S3J MyoD

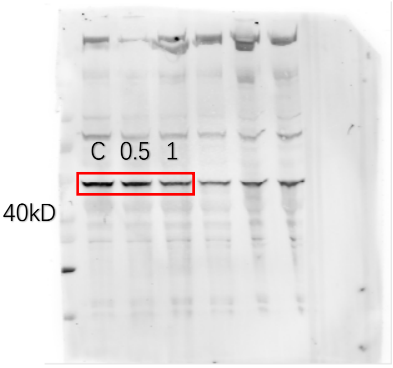

Fig S3J MyoG

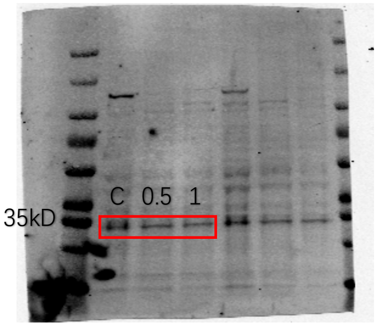

Fig S3J Atrogin-1

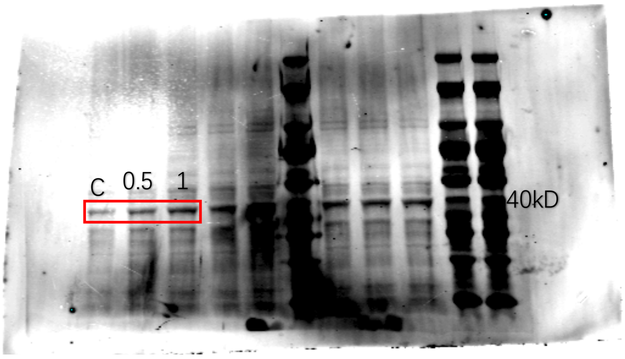

Fig S3J MuRF-1

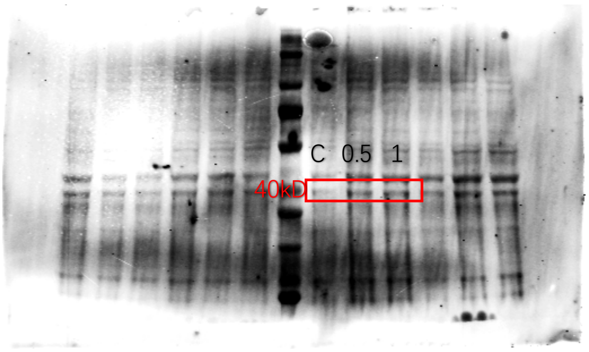

Fig S3J GAPDH

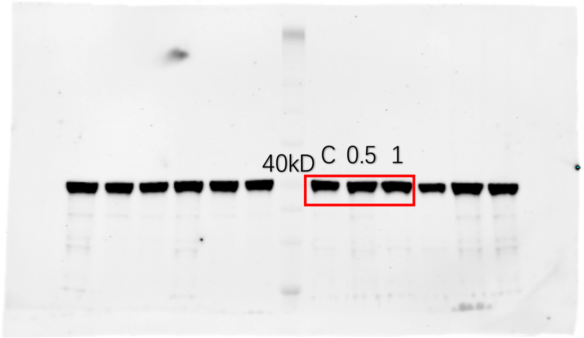

Fig S4F TA Anxa2

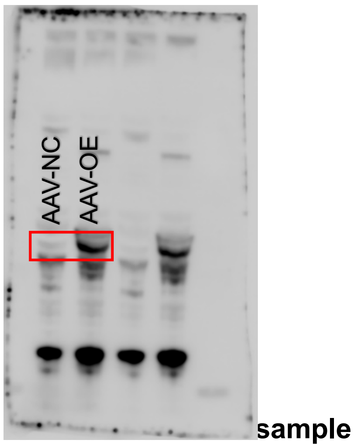

sample

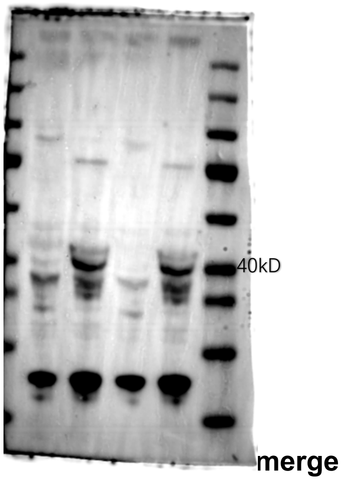

merge

Fig S4F TA Flag

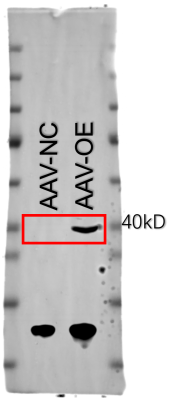

Fig S4F TA Gapdh

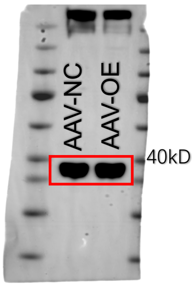

Fig S4F G Anxa2

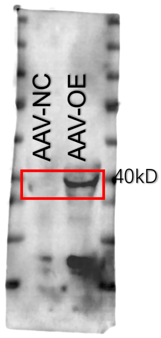

Fig S4F G Flag

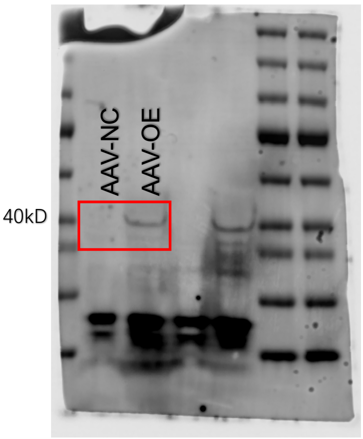

Fig S4F G Gapdh

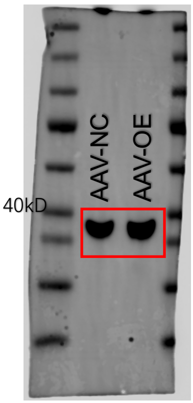

Fig S4F Q Anxa2

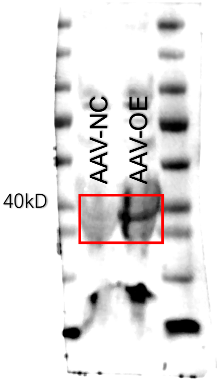

Fig S4F Q Flag

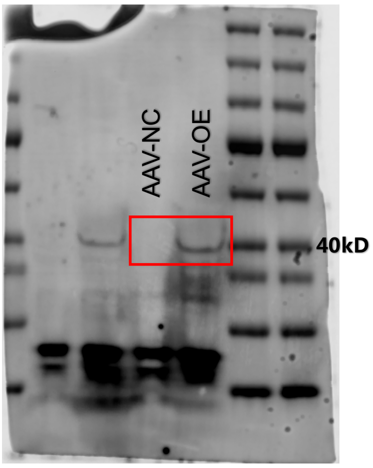

Fig S4F Q Gapdh

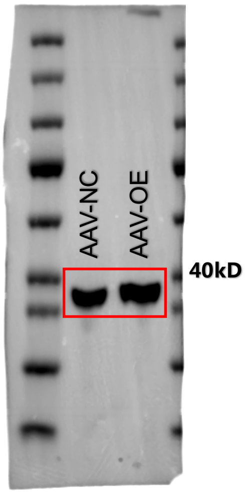

Fig S4H Heart Anxa2

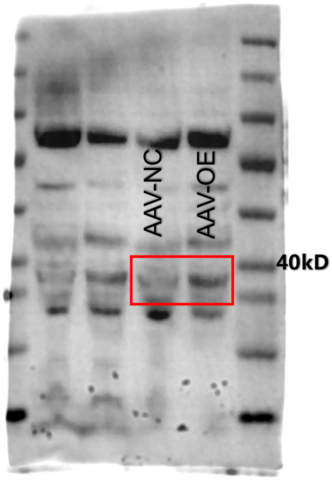

Fig S4H Heart Gapdh

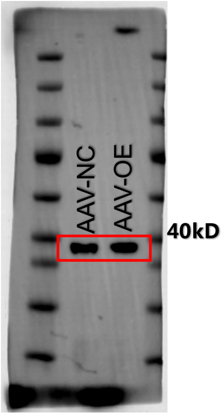

Fig S4H Liver Anxa2

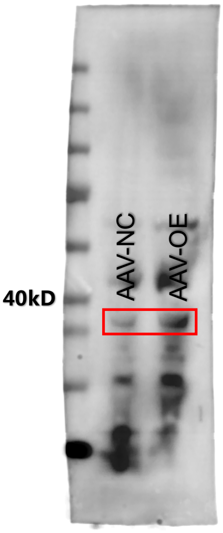

FigS4H Liver Gapdh

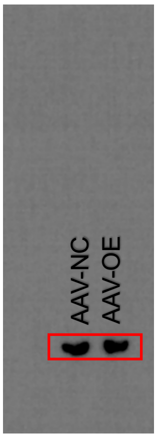

sample

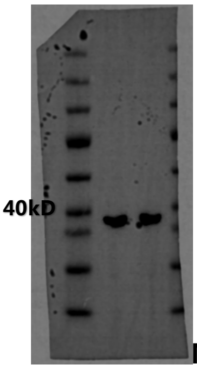

Merge

Fig S4I Anxa2

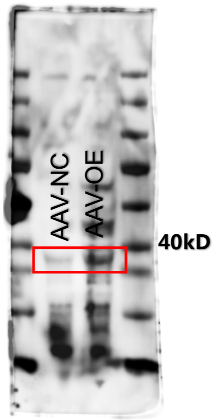

Fig S4I Gapdh

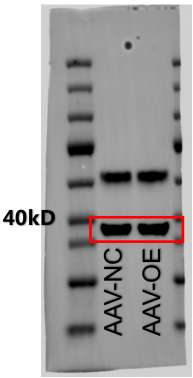

Fig S4O Anxa2

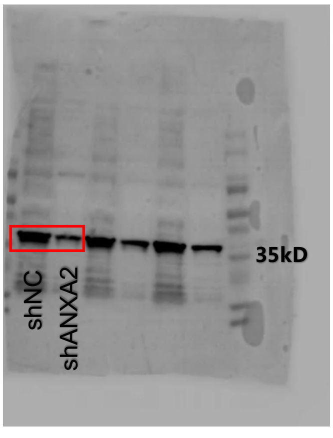

Fig S4O Gapdh

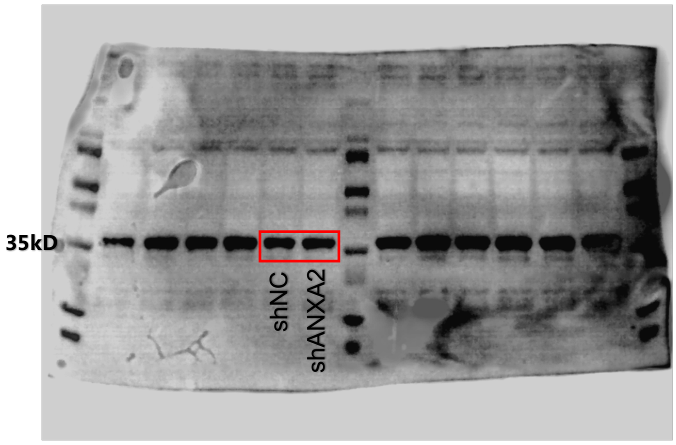

Fig S5C Anxa2

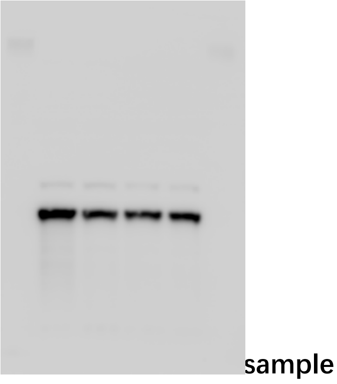

sample

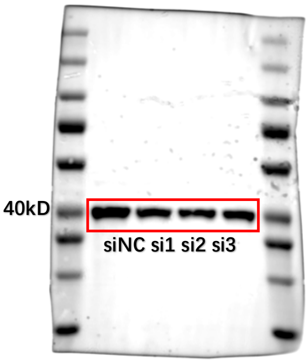

merge

Fig S5C gapdh

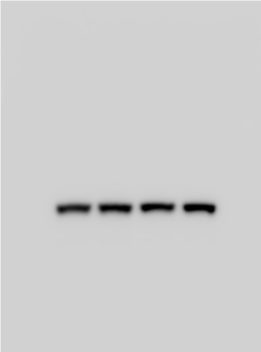

sample

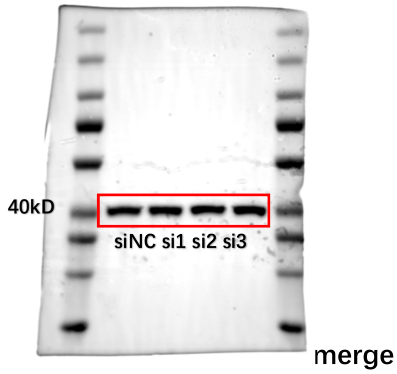

Fig S5D MyoD

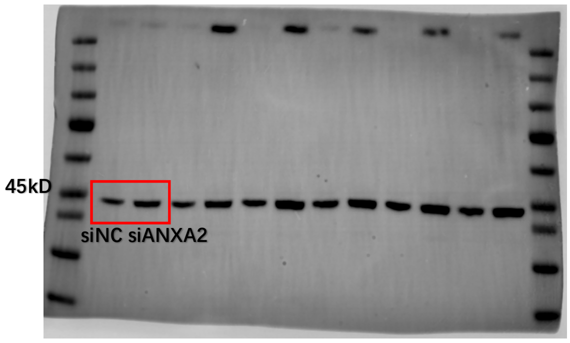

Fig S5D MyoG

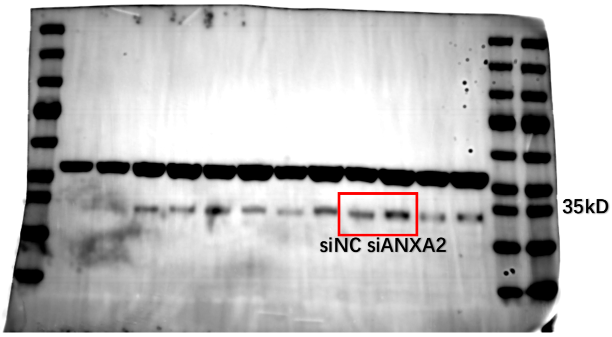

Fig S5D Atrogin-1

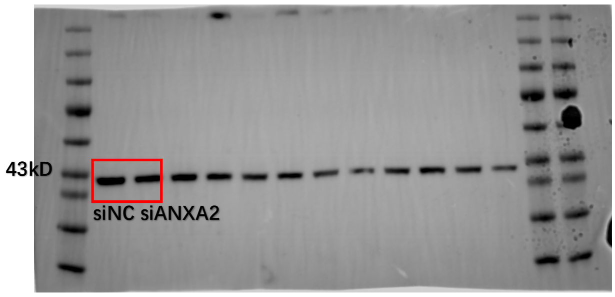

Fig S5D MuRF-1

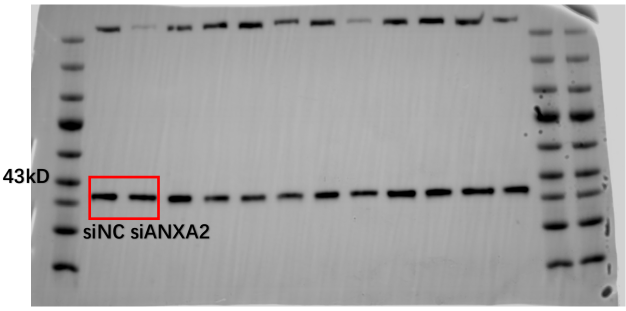

Fig S5D Gapdh

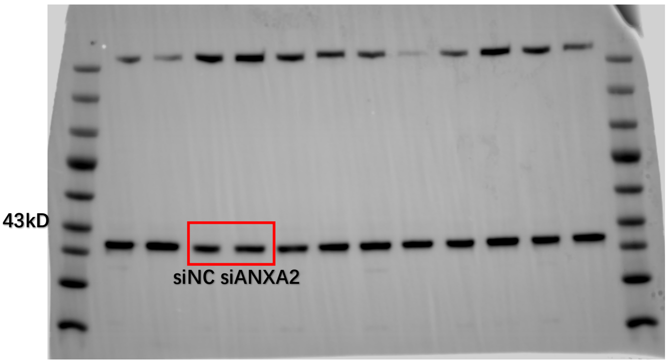

Fig S5F Neu2

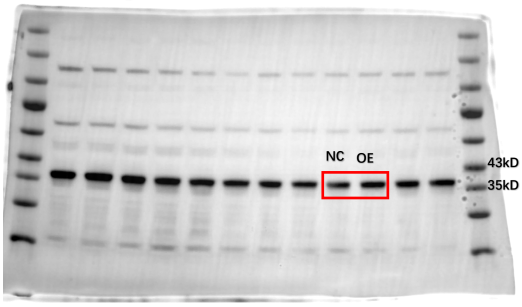

Fig S5F Gapdh

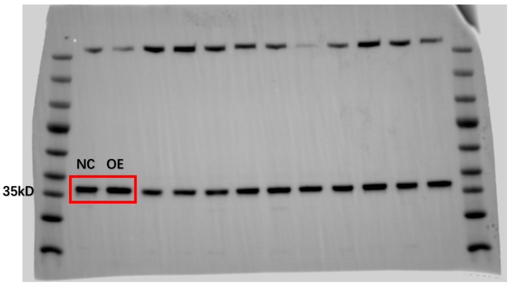

Fig S6A Neu2

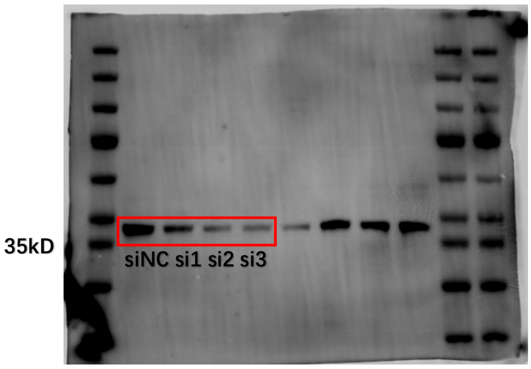

Fig S6A Gapdh

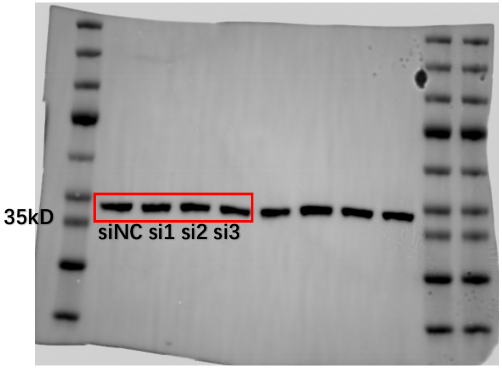

Fig S6C MyoD

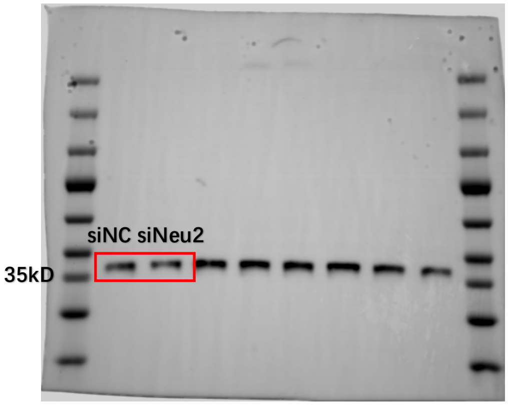

Fig S6C MyoG

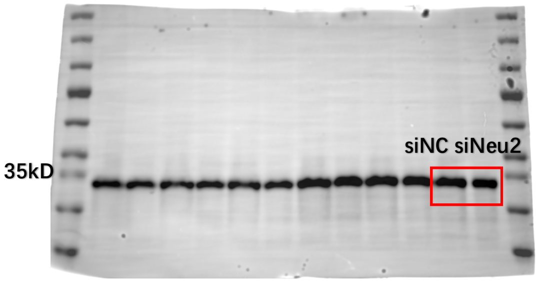

Fig S6C MuRF-1

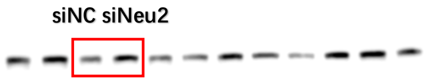

sample

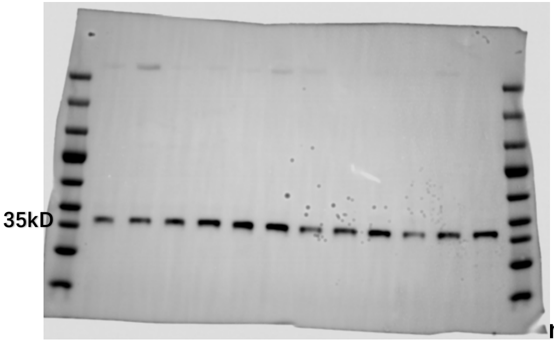

merge

Fig S6C Atrogin-1

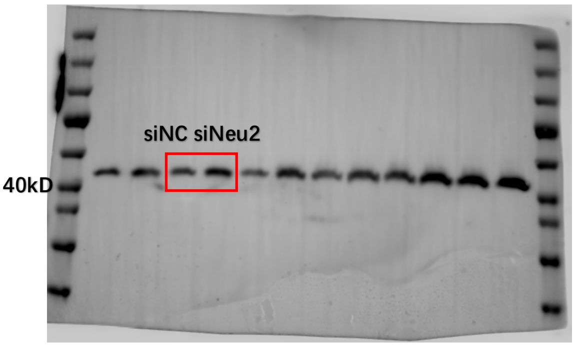

Fig S6C Gapdh

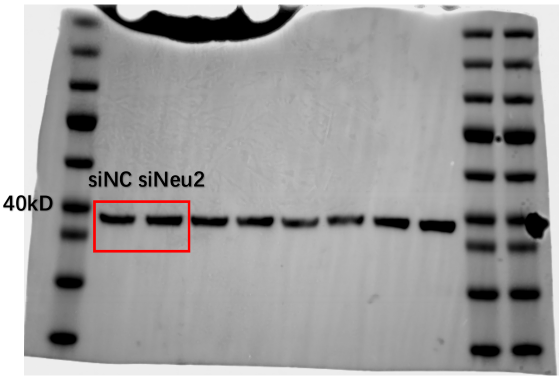

Fig S8E CB2R

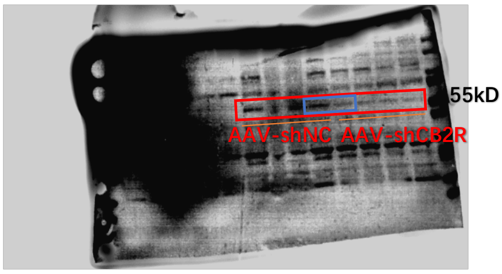

Fig S8E actin

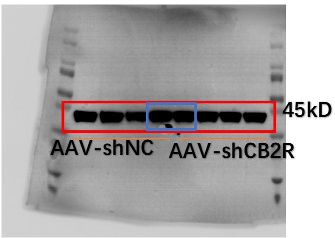

Fig S9B Anxa2

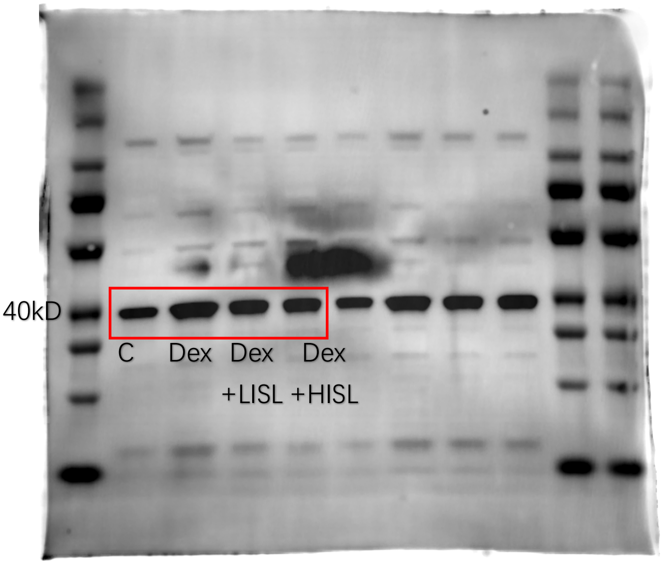

Fig S9B Gapdh

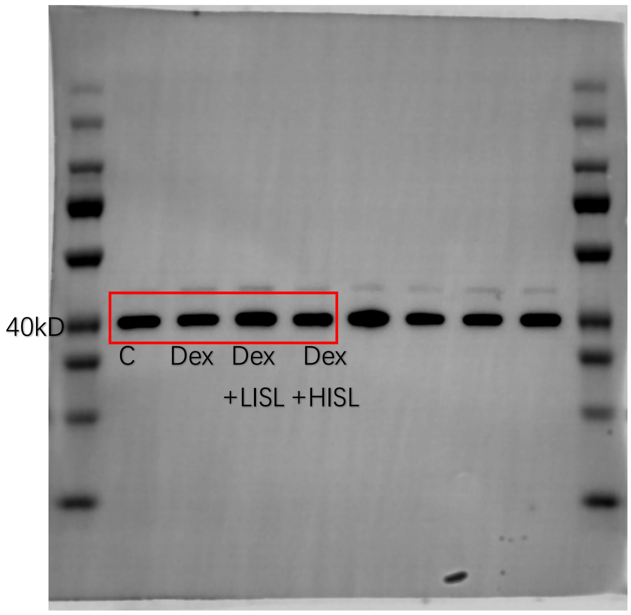
40kD
C
Dex
Dex
Dex
+LISL
+HISL
