# Supplementary material for: Annexin A2 Causes Motor Incoordination via Muscle–Cerebellum Axis in Sarcopenia
Source: J Cachexia Sarcopenia Muscle. 2026 Jan 26;17(1):e70203. doi: 10.1002/jcsm.70203 (PMC12835187; doi:10.1002/jcsm.70203)
Supplement: Supplementary file 3 — Figure S1: Motor coordination and muscle function of aged mice. Related to Figure 1. (A) Schematic diagram on the timeline of balance beam test procedure (left) and running time of young and aged mice on 12 mm (middle) and 6 mm (right) balance beam tests (n = 10). (B) Schematic diagram on the timeline of pole test procedure (left) and running time (middle) and total time (right) of young and aged mice in pole test (n = 10). (C) The average speed (left), body speed (middle) and swing speed (right) of young and aged mice in gait analysis (n = 8–9). (D) Cadence (left), stand time (right) and swing time (right) of young and aged mice (n = 8–9). (E) Support proportion of young and aged mice in gait analysis (n = 8–9). (F) The masses of extensor digitorum longus, soleus and plantaris muscles of young and aged mice (n = 10). (G) Representative images and cross sectional area (CSA) of gastrocnemius in young and aged mice (n = 3). Values are represented as means ± SEM. Exact p values are shown. RF represents right front. RH represents right hind. LF represents left front. LH represents left hind. Figure S2: ANXA2 increased both in muscles and cerebellums of aged mice. Related to Figure 2. (A) Heatmap (left) and volcano plot (right) of differential expressed proteins of muscles in young and aged mice. (B) Heatmap (left) and volcano plot (right) of differential expressed proteins of cerebellums in young and aged mice. (C) The RNA levels of ANXA2 in tibialis anterior (TA, left) and gastrocnemius (G, right) in young and aged mice by qRT‐PCR (n = 3). (D) The protein levels of ANXA2 in liver, lung, kidney, spleen and heart in young and aged mice (left) and statistical results (right) by Western Blot (n = 4 or 6). (E) HE staining of TA in young (Young‐H) and aged (Aged‐H) human. (F) Representative images of MyHC staining of C2C12 myotubes in Mock and Dexamethasone (Dex) groups (left) and statistical results of diameters of myotubes by immunofluorescence (right) (n = 8). (G) The RNA [file JCSM-17-e70203-s003.docx]

Supplementary Figures


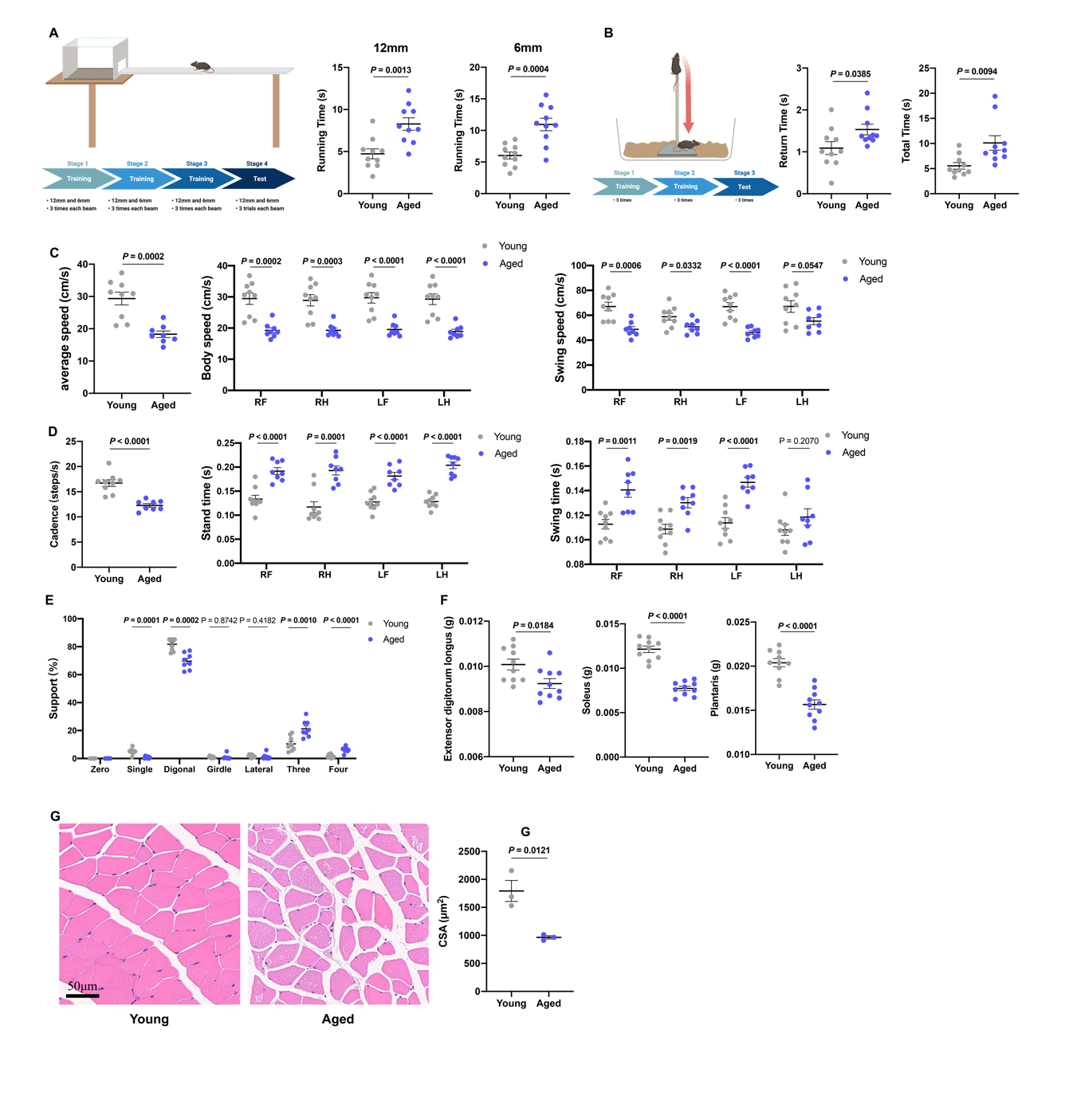


**Fig S1.** **Motor coordination and muscle function of aged mice. Related to Fig 1. A.** Schematic diagram on the timeline of balance beam test procedure (left) and running time of young and aged mice in 12mm (middle) and 6mm (right) balance beam tests (n=10). **B.** Schematic diagram on the timeline of pole test procedure (left) and Running time (middle) and Total time (right) of young and aged mice in pole tests (n=10). **C.** The average speed (left), body speed (middle), and swing speed (right) of young and aged mice in gait analysis. (n=8-9). **D.** Cadence (left), stand time (right), and swing time (right) of young and aged mice. (n=8-9). **E.** Support proportion of young and aged mice in gait analysis. (n=8-9) **F.** The masses of Extensor digitorum longus, Soleus, Plantaris muscles of young and aged mice. (n=10). **G.** Representative images and cross sectional area (CSA) of Gastrocnemius in young and aged mice (n=3). Values are represented as means ± s.e.m. Exact P values are shown. RF represents right front. RH represents right hind. LF represents left front. LH represents left hind.


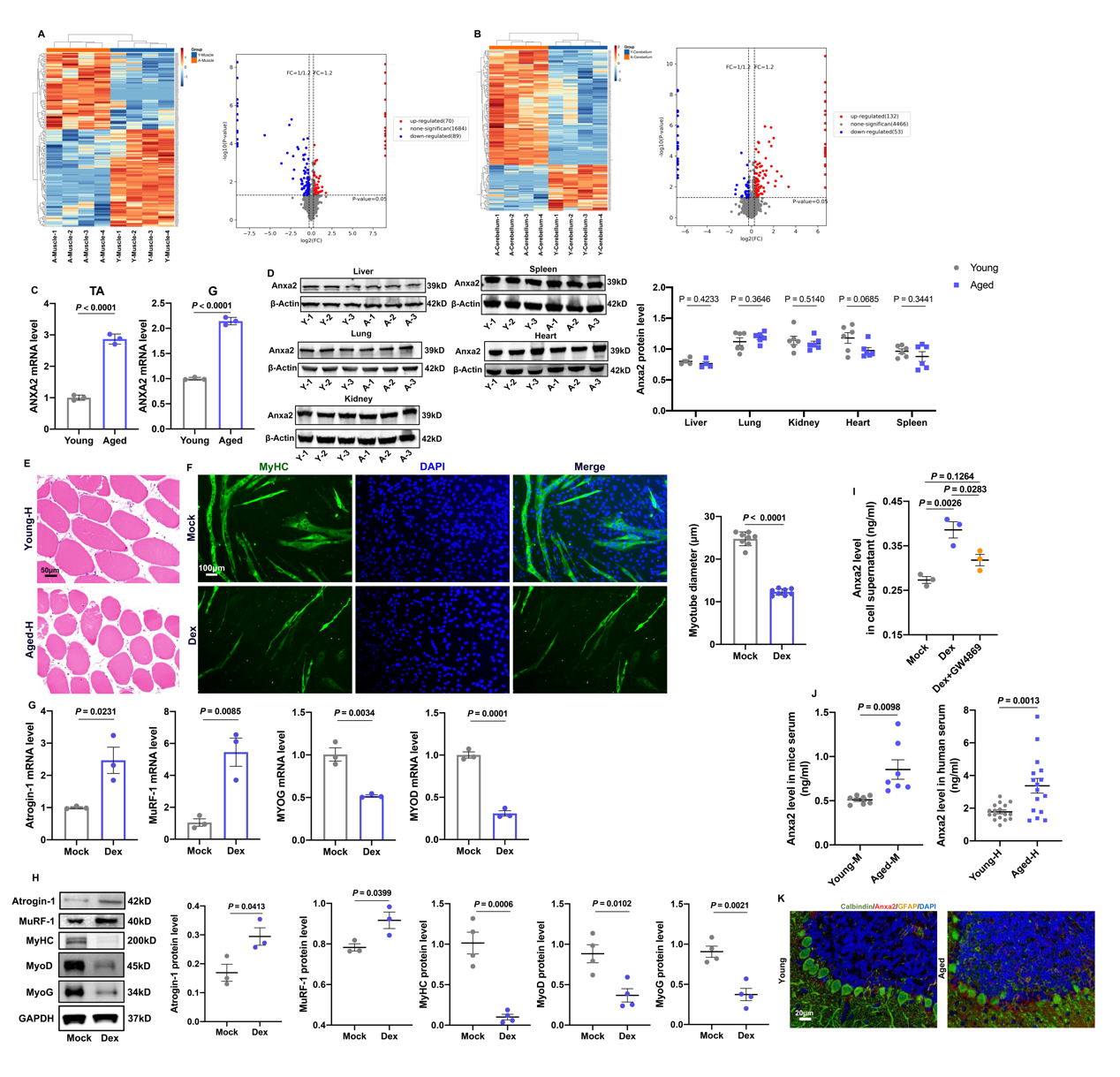


**Fig S2. ANXA2 increased in both muscles and cerebellums of aged mice. Related to Fig 2. A.** Heatmap (left) and Volcano Plot (right) of differential expressed proteins of muscles in young and aged mice. **B.** Heatmap (left) and Volcano Plot (right) of differential expressed proteins of cerebellums in young and aged mice. **C.** The RNA levels of ANXA2 in Tibialis anterior (TA, left) and Gastrocnemius (G, right) in young and aged mice by qRT-PCR (n=3). **D.** The protein levels of ANXA2 in livers, lungs, kidneys, spleens, hearts in young and aged mice (left) and statistical results (right) by Western Blot (n=4 or 6). **E.** HE staining of Tibialis anterior in young (Young-H) and aged (Aged-H) human. **F.** Representative images of MyHC staining of C2C12 myotubes in Mock and Dexamethasone (Dex) groups (left) and statistical results of diameters of myotubes by immunofluorescence (right) (n=8). **G.** The RNA levels of Atrogin-1, MuRF-1, MYOG, MYOD in C2C12 myotubes in Mock and Dex groups by qRT-PCR (n=3). **H.** The protein levels and statistical results of Atrogin-1, MuRF-1, MyHC, MyoG and MyoD in C2C12 myotubes by Western Blot (n=3-4). **I.** The level of ANXA2 in the supernatant of C2C12 myotubes in the Mock, Dex, Dex+GW4869 groups by ELISA (n=3). **J.** The level of ANXA2 in mice (Young-M represents young mice, Aged-M represents aged mice) and human (Young-H represents young human, Aged-H represents aged human) serum by ELISA (n=7; n=17 or 16). **K.** The expression of ANXA2 in cerebellums from young and aged mice by immunofluorescence. Values are represented as means ± s.e.m. Exact P values are shown. “A” represents aged mice, “Y” represents young mice.


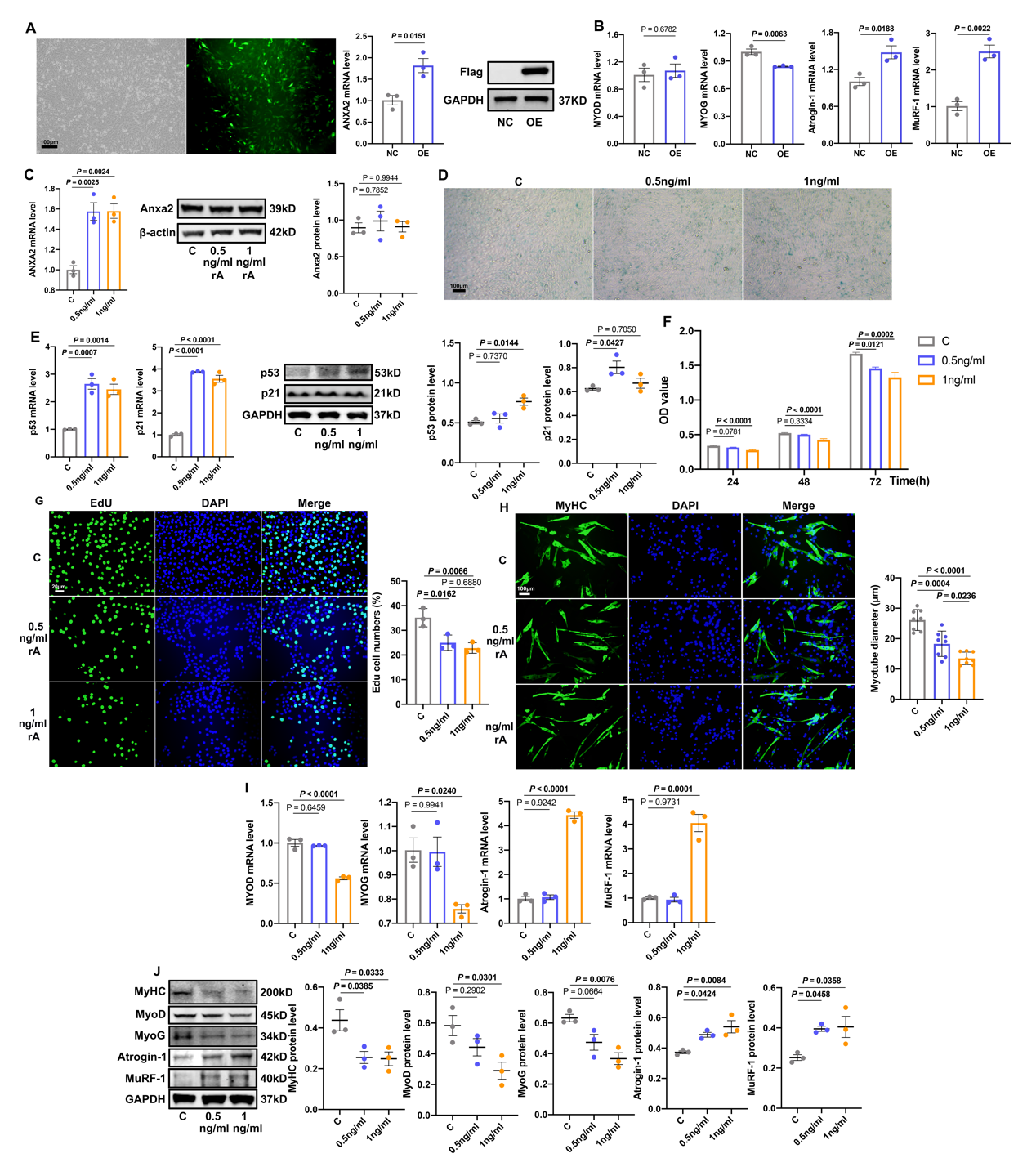


**Fig S3. Effects of exogenous ANXA2 (recombinant ANXA2, rA) on proliferation, senescence of C2C12 cells and atrophy of C2C12 myotubes. A.** EGFP fluorescence of Adenovirus (left) and the RNA level of ANXA2 (middle) and protein level of Flag (right) in C2C12 cells in NC and OE groups (n=3). **B.** The RNA levels of MYOD, MYOG, Atrogin-1, MuRF-1 in C2C12 myotubes in NC and OE groups by qRT-PCR (n=3). **C.** The RNA (left) and protein (middle) levels and statistical results (right) of ANXA2 in C2C12 cells in C, 0.5ng/ml rA and 1ng/ml rA groups (n=3). **D.** S-β-Gal staining of C2C12 cells in C, 0.5ng/ml rA and 1ng/ml rA groups. **E.** The RNA and protein levels and statistical results of p53 and p21 in C2C12 cells in C, 0.5ng/ml rA and 1ng/ml rA groups (n=3). **F.** OD values of C2C12 cells in C, 0.5ng/ml rA and 1ng/ml rA groups by CCK-8 (n=6). **G.** Representative images of EdU positive C2C12 cells in C, 0.5ng/ml rA and 1ng/ml rA groups (left) and statistical results (right) by immunofluorescence (n=3). **H.** Representative images of MyHC staining of C2C12 myotubes in C, 0.5ng/ml rA and 1ng/ml rA groups (left) and statistical results of diameters of myotubes (right) by immunofluorescence (n=8). **I.** The RNA levels of MYOG, MYOD, MuRF-1, Atrogin-1 in C2C12 myotubes in C, 0.5ng/ml rA and 1ng/ml rA groups by qRT-PCR (n=3). **J.** The protein levels and statistical results of MyHC, MyoD, MyoG, Atrogin-1, MuRF-1 in C2C12 myotubes in C, 0.5ng/ml rA and 1ng/ml rA groups by Western Blot (n=3). Values are represented as means ± s.e.m. Exact P values are shown. “C” represents control group. “0.5ng/ml” represents 0.5ng/ml rA group. “1ng/ml” represents 1ng/ml rA group.


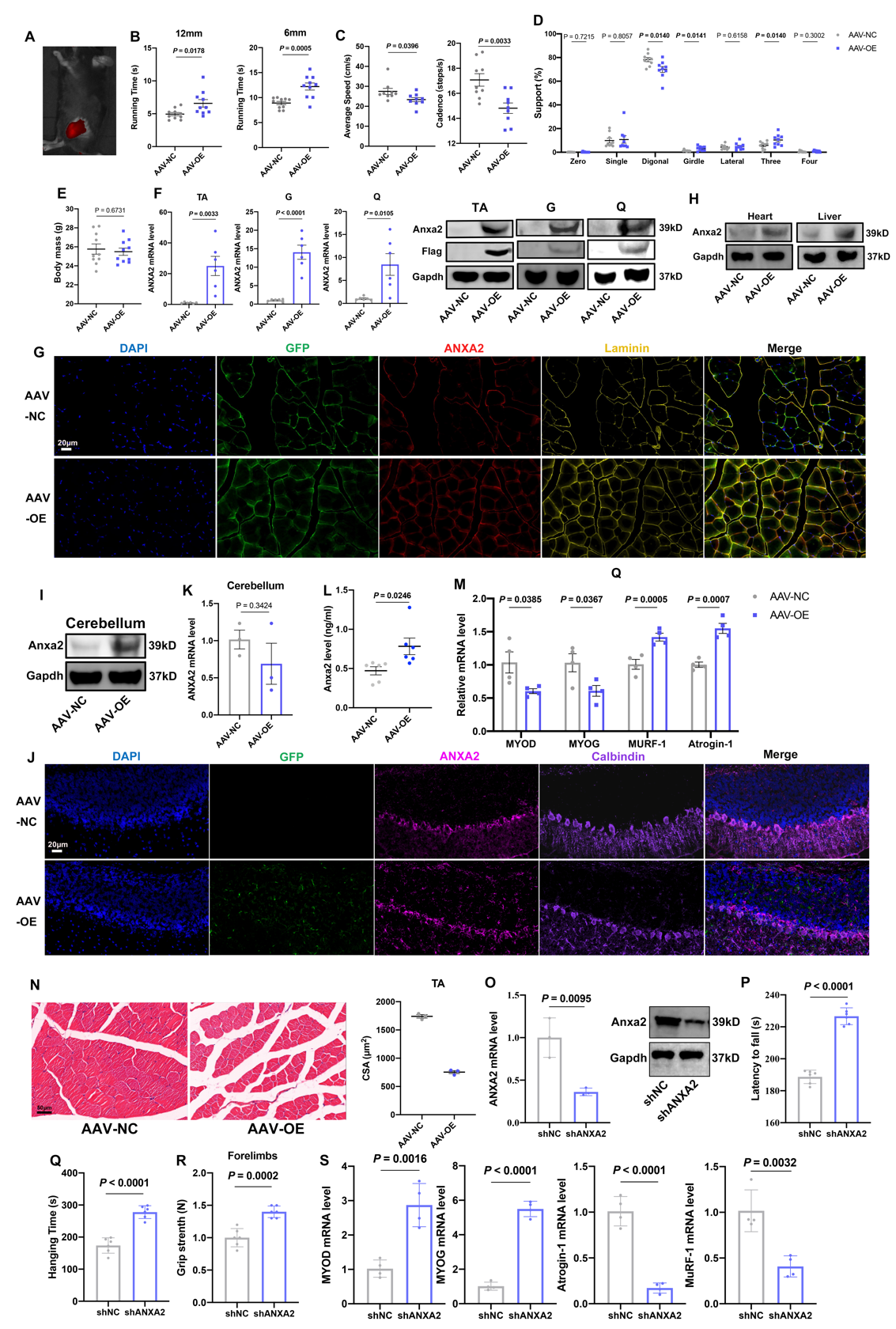


**Fig S4. Effects of ANXA2 overexpression in muscles on behavioral performance. Related to Fig 4. A.** Representative fluorescence images of mice at the 3rd week after AAV intramuscular injection. **B.** Running times of mice in AAV-NC and AAV-OE groups in 12mm (left) and 6mm (right) balance beam tests (n=10). **C.** The average speed (left) and cadence (right) of mice in AAV-NC and AAV-OE groups in gait analysis (n=9). **D.** The support proportion of mice in AAV-NC and AAV-OE groups in gait analysis (n=9). **E.** Body masses of mice in AAV-NC and AAV-OE groups (n=10). **F.** The RNA and protein levels of ANXA2 in Tibialis anterior (TA), Gastrocnemius (G), Quadriceps femoris (Q) of mice in AAV-NC and AAV-OE groups. **G.** The expression of GFP, ANXA2 and Laminin in Tibialis anterior of mice in AAV-NC and AAV-OE groups by immunofluorescence. **H.** The protein levels of hearts (left) and livers (right) of mice in AAV-NC and AAV-OE groups. **I.** The protein level of ANXA2 in cerebellums of mice in AAV-NC and AAV-OE groups. **J.** The expression of GFP, ANXA2 and Calbindin in Cerebellums of mice in AAV-NC and AAV-OE groups by immunofluorescence. **K.** The RNA levels of Cerebellum of mice in AAV-NC and AAV-OE groups by qRT-PCR (n=3). **L.** The ANXA2 level in serum of mice in AAV-NC and AAV-OE groups by ELISA (n=6). **M.** The RNA levels of MYOD, MYOG, MuRF-1, Atrogin-1 in Quadriceps muscles of mice in AAV-NC and AAV-OE groups by qRT-PCR (n=4). **N.** Representative images (left) and cross sectional area (CSA, right) of Tibialis anterior muscles of mice in AAV-NC and AAV-OE groups (n=3). **O.** The RNA and protein level of ANXA2 in muscles of mice in shNC and shANXA2 groups. **P.** AR latency to fall of mice in shNC and shANXA2 groups (n=6). **Q.** Hanging times of mice in shNC and shANXA2 groups (n=6). **R.** Grip Strengths of mice in shNC and shANXA2 groups (n=6). **S.** The RNA levels of MYOD, MYOG, Atrogin-1 and MuRF-1 in muscles of mice in in shNC and shANXA2 groups (n=4). Values are represented as means ± s.e.m. Exact P values are shown.


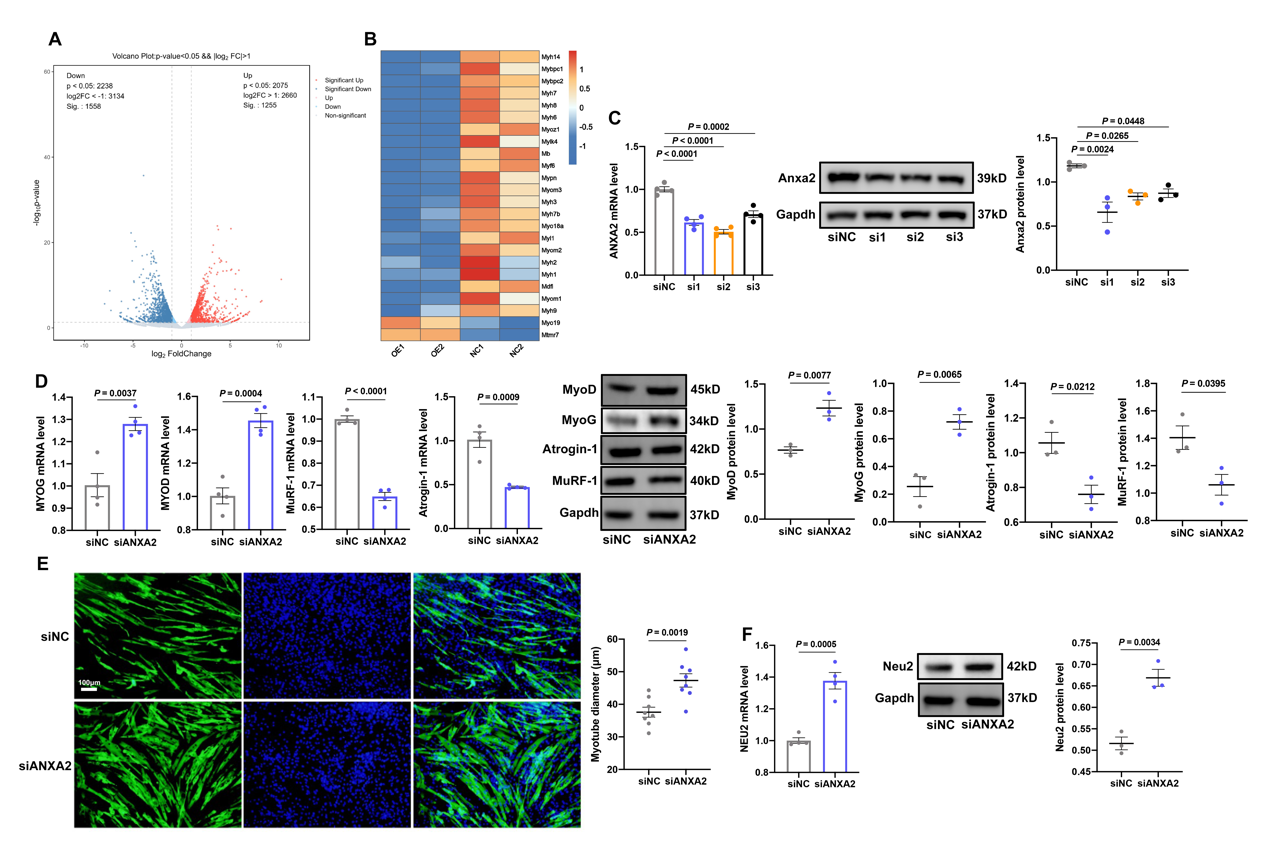


**Fig S5. Effects of ANXA2 knockdown on myogenic differentiation of C2C12 cells. Related to Fig 5. A.** Volcano Plot of differential expressed genes in C2C12 myotubes in NC and OE groups. **B.** Heatmap of differential expressed genes related to muscles in C2C12 myotubes in NC and OE groups. **C.** The RNA (left) and protein (middle) levels and statistical results (right) of ANXA2 in C2C12 cells in siNC, siANXA2-1, siANXA2-2 and siANXA2-3 groups by qRT-PCR and Western Blot (n=3-4). **D.** The RNA and protein levels and statistical results of MYOG, MYOD, MuRF-1 and Atrogin-1 in C2C12 myotubes in siNC and siANXA2 groups by qRT-PCR and Western Blot (n=3-4). **E.** Representative images of MyHC staining of C2C12 myotubes in siNC and siANXA2 groups (left) and statistical results of diameters of myotubes (right) by immunofluorescence (n=8). **F.** The RNA (left) and protein (middle) levels and statistical results (right) of Neu2 in C2C12 myotubes in siNC and siANXA2 group (n=3-4). Values are represented as means ± s.e.m. Exact P values are shown.


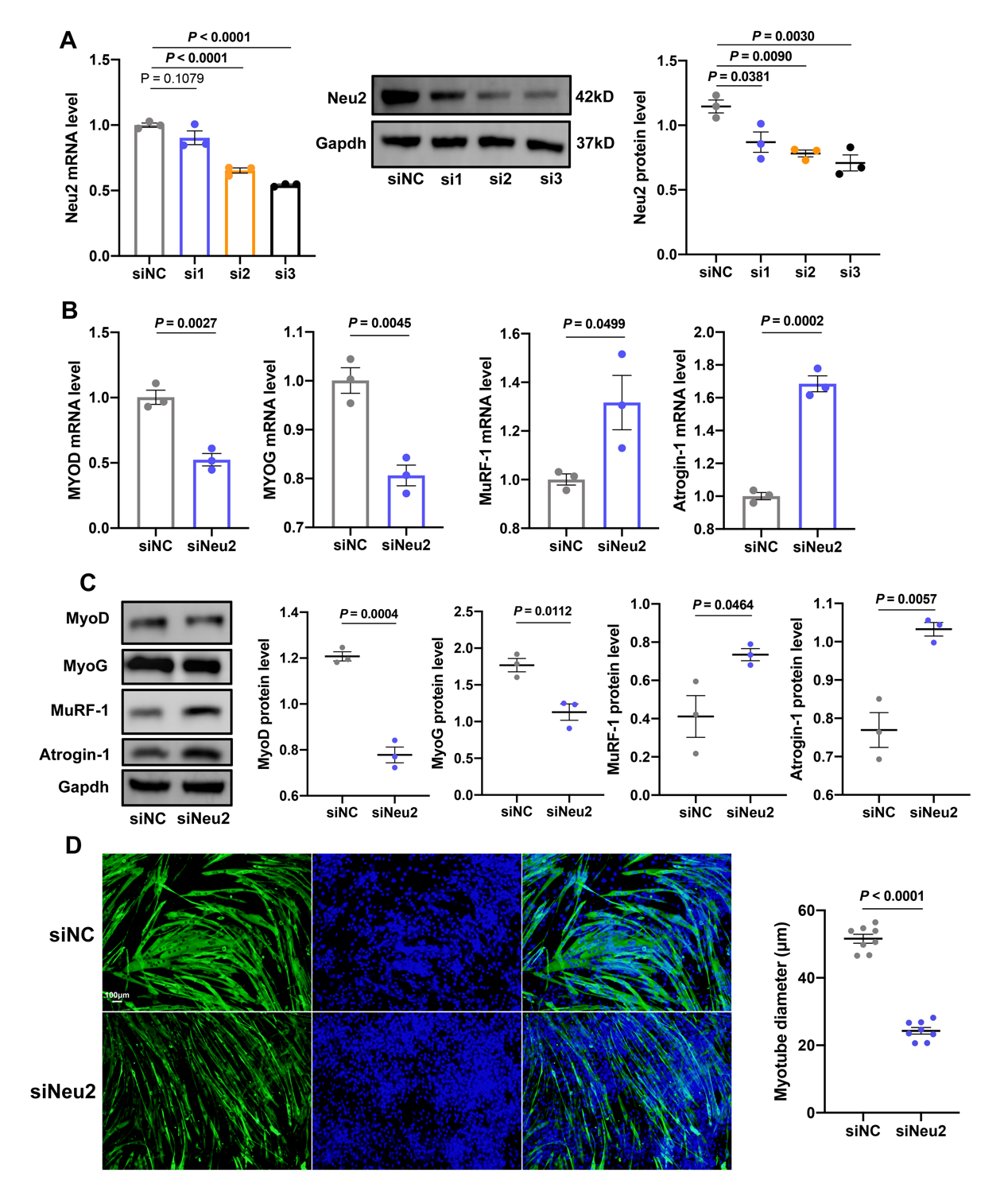


**Fig S6. Effects of NEU2 knockdown on myogenic differentiation of C2C12 cells. Related to Fig 5. A.** The RNA (left) and protein (middle) levels and statistical results (right) of NEU2 in C2C12 cells in siNC, siNEU2-1, siNEU2-2 and siNEU2-3 groups by qRT-PCR and Western Blot (n=3). **B.** The RNA levels of MYOD, MYOG, MuRF-1 and Atrogin-1 in C2C12 myotubes in siNC and siNEU2 groups by qRT-PCR (n=3). **C.** The protein levels (left) and statistical results (right) of MyoD, MyoG, MuRF-1, Atrogin-1 in C2C12 myotubes in siNC and siNEU2 groups by Western Blot (n=3). **D.** Representative images of MyHC staining of C2C12 myotubes in siNC and siNeu2 groups (left) and statistical results of diameters of myotubes (right) by immunofluorescence (n=8). Values are represented as means ± s.e.m. Exact P values are shown.


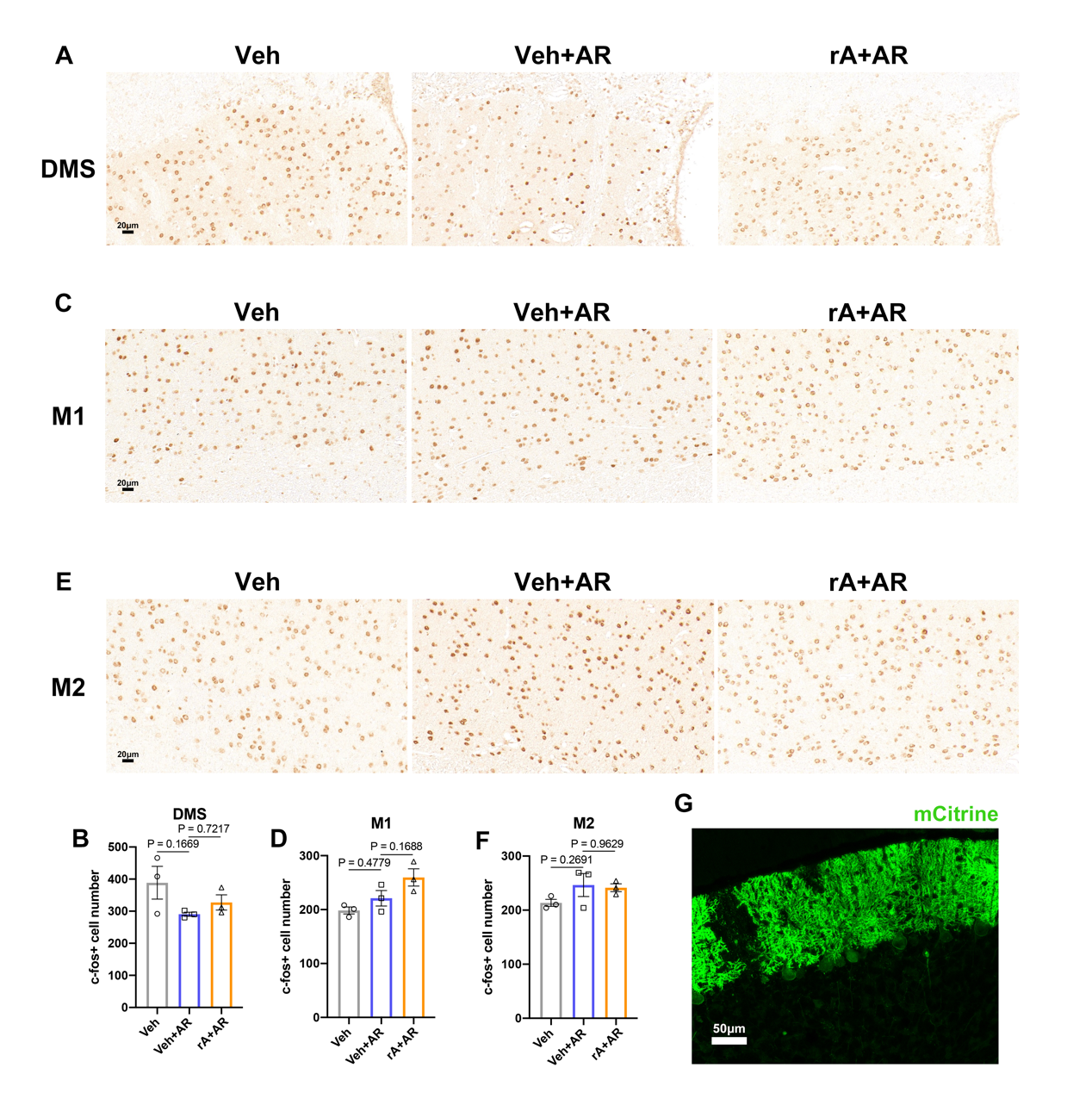


**Fig S7. Effects of ANXA2 on neuron activation in DMS, M1 and M2. Related to Fig 6.** **A, B.** Representative images (**A**) and numbers (**B**) of c-Fos positive cells in DMS of mice in Vehicle (Veh), AR test (Veh+AR), rA injection and AR test (rA+AR) groups (n=3). **C, D.** Representative images (**C**) and numbers (**D**) of c-Fos positive cells in M1 of mice in Veh, Veh+AR, rA+AR groups (n=3). **E, F.** Representative images (**E**) and numbers (**F**) of c-Fos positive cells in M2 of mice in Veh, Veh+AR, rA+AR groups (n=3). **G.** Fluorescence of cerebellum after AAV9-L7-6-hM3D(Gq)-mCitrine infection. Values are represented as means ± s.e.m. Exact P values are shown. DMS represents Dorsomedial Striatum. M1 represents primary motor cortex. M2 represents secondary motor cortex.


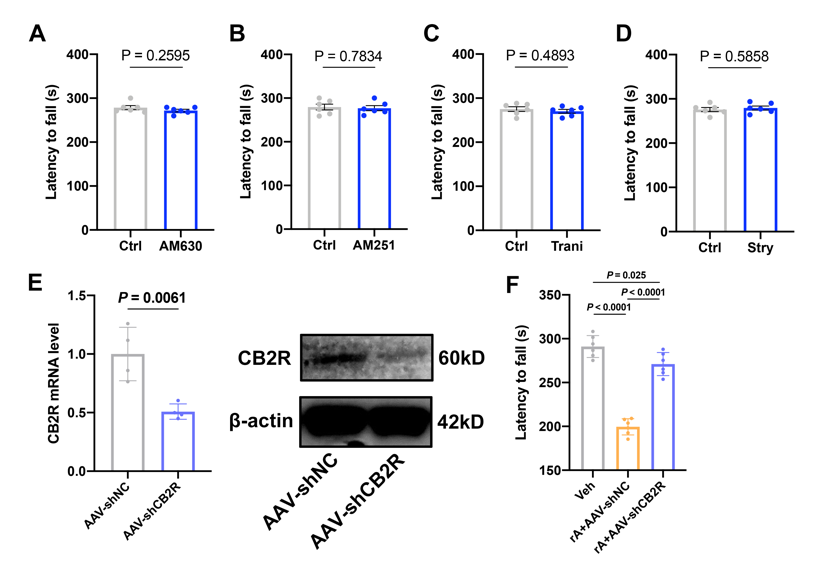


**Fig S8. Effects of various antagonists on motor coordination in mice. Related to Fig7.** **A.** Effects of AM630 systemic injection on AR latency to fall (n=6). **B.** Effects of AM251 systemic injection on AR latency to fall (n=6). **C.** Effects of Tranilast (Trani) systemic injection on AR latency to fall (n=6). **D.** Effects of Strychnine (Stry) systemic injection on AR latency to fall (n=6). **E.** The RNA and protein level in cerebellums of mice in AAV-NC and AAV-shCB2R groups. **F.** AR latency to fall of mice in Vehicle (Veh), AAV-shNC infection and rA injection (rA+AAV-shNC), AAV-shCB2R infection and rA injection (rA+AAV-shCB2R) groups (n=6). Values are represented as means ± s.e.m. Exact P values are shown.


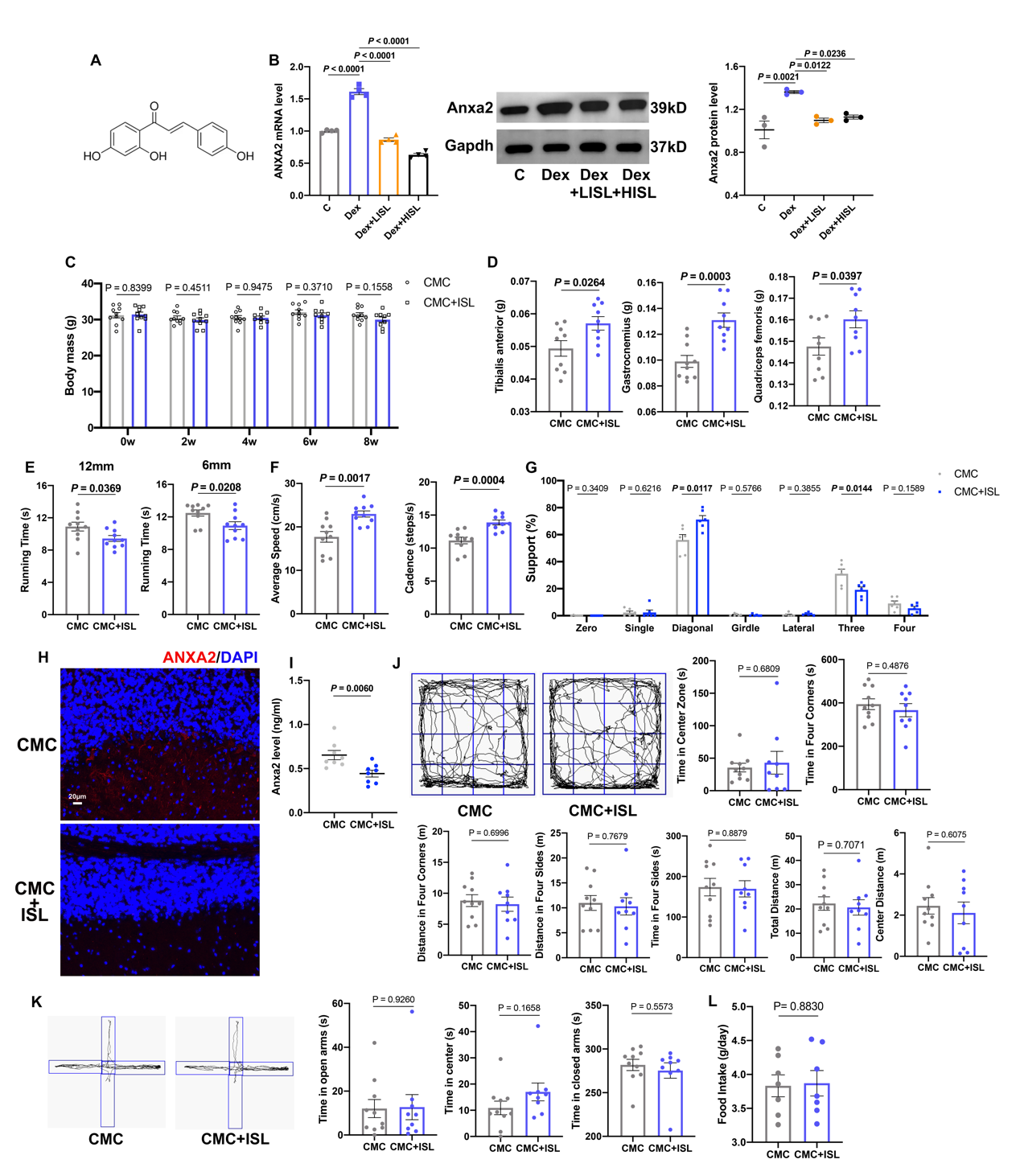


**Fig S9. Effects of Isoliquiritigenin on motor coordination and muscle function of aged mice. Related to Fig.8. A.** Molecular structure of Isoliquiritigenin (ISL). **B.** The RNA (left) and protein (middle) level and statistical results (right) of ANXA2 in C2C12 myotubes in Control (C) group, Dexamethasone (Dex) group, Dex and 0.5ng/ml ISL (Dex+LISL) group and Dex and 1ng/ml ISL (Dex+HISL) group by qRT-PCR and Western Blot (n=3-4). **C.** The body mass of mice during ISL gavage in CMC and CMC+ISL groups (n=9-10). **D.** The mass of Tibialis Anterior, Gastrocnemius and Quadriceps femoris of mice in CMC and CMC+ISL groups (n=9-10). **E.** Running time of mice in CMC and CMC+ISL groups on 12mm (left) and 6mm (right) balance beam tests (n=10). **F.** The average speed (left) and cadence (right) of mice in CMC and CMC+ISL groups in gait analysis (n=10). **G.** The support proportion of mice in CMC and CMC+ISL groups in gait analysis (n=6). **H.** The ANXA2 expression in cerebellums of mice in CMC and CMC+ISL groups by immunofluorescence. **I.** The ANXA2 level of serum in mice in CMC and CMC+ISL groups by ELISA (n=8). **J.** Representative images of open field test and time in center zone, four corners and four sides, total distance, center distance, distance in four corners, four sides of mice in CMC and CMC+ISL groups (n=9-10). **K.** Representative images of elevated plus maze and time in open arms, center and closed arms of mice in CMC and CMC+ISL groups (n=9-10). **L.** Food intake of mice in CMC and CMC+ISL groups (n=7). Values are represented as means ± s.e.m. Exact P values are shown. CMC represents 0.5% sodium carboxyl methyl cellulose (CMC-Na). CMC+ISL represents 20mg/kg ISL (in 0.5% CMC-Na).


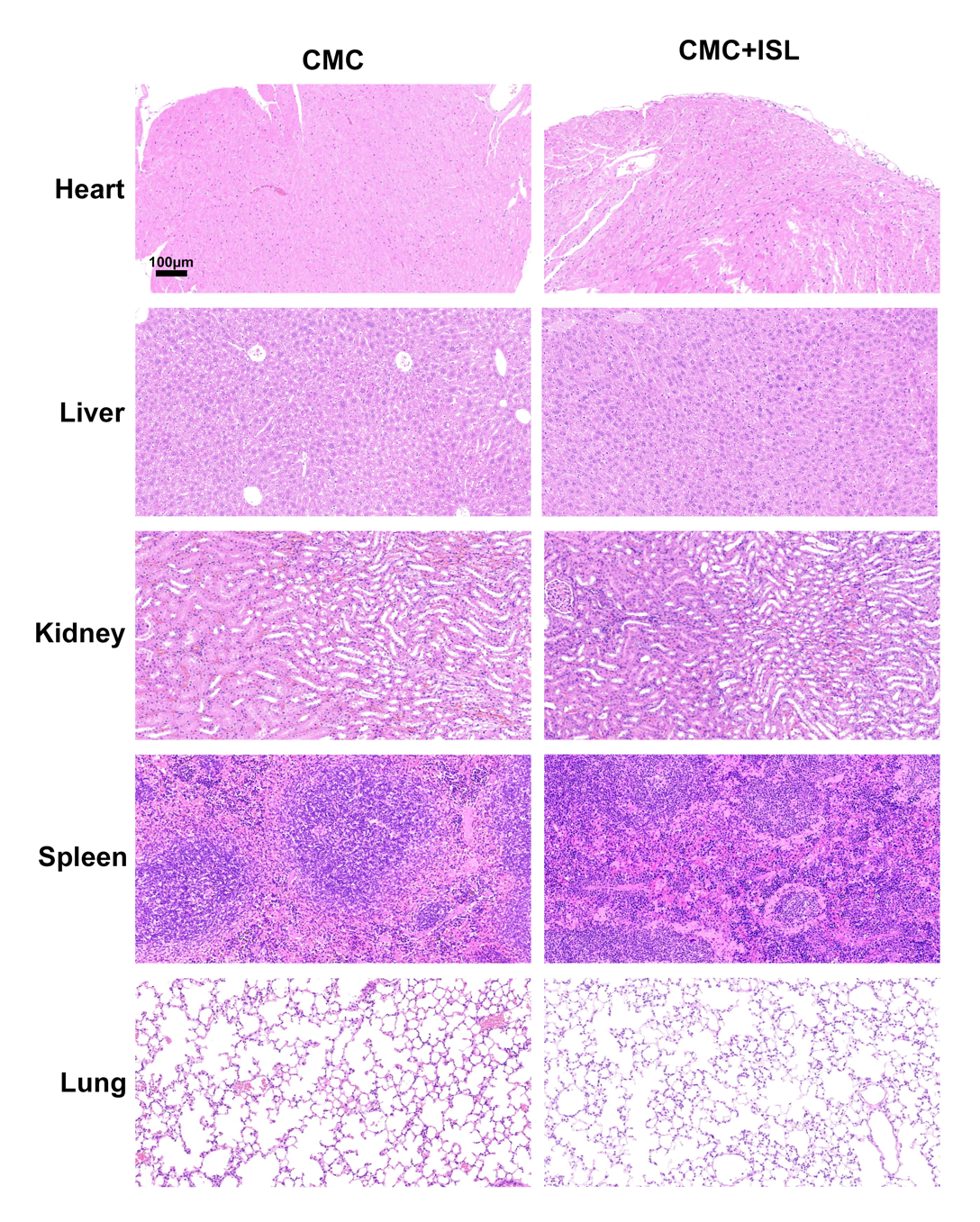


**Fig S10. HE staining of Heart, Liver, Kidney, Spleen and Lung of mice in CMC and CMC+ISL groups. Related to Fig. 8.**
