# Supplementary material for: Annexin A2 Causes Motor Incoordination via Muscle–Cerebellum Axis in Sarcopenia
Source: J Cachexia Sarcopenia Muscle. 2026 Jan 26;17(1):e70203. doi: 10.1002/jcsm.70203 (PMC12835187; doi:10.1002/jcsm.70203)
Supplement: Supplementary file 4 — Table S1: The ANXA2 level in cerebellums of mice after saline and rA i.p. injection (ng/mL). Table S2: Sequences of primers for qRT‐PCR. Table S3: Sequences of shRNA for ANXA2 and CB2R. Table S4: Sequences of siRNA for ANXA2 and Neu2. [file JCSM-17-e70203-s002.docx]

Supplementary Tables

**Table S1. The ANXA2 level in cerebellums of mice after saline and rA i.p. injection (ng/ml)**

| saline-cerebellum | rA-cerebellum |
| --- | --- |
| <0.05 | <0.05 |
| <0.05 | 0.064128 |
| <0.05 | 0.08083 |
| <0.05 | <0.05 |
| 0.085778 | 0.1286 |
| <0.05 | 0.100749 |

**Table S2. Sequences of Primers for qRT-PCR**

| Genes | Forward | Reverse |
| --- | --- | --- |
| GAPDH | AGGTCGGTGTGAACGGATTTG | GGGGTCGTTGATGGCAACA |
| ANXA2 | ACGACTCCATGAAGGGCAAG | CCTTCAGTCATCCCCACCAC |
| MuRF-1 | GGGCCATTGACTTTGGGACA | TCTCCTTCTTCATTGGTGTTCTTCT |
| Atrogin-1 | AGAGAGGCAGATTCGCAAGCGT | TGCAAAGCTGCAGGGTGACCC |
| MYOD | CTTCTATCGCCGCCACTC | AAGTCGTCTGCTGTCTCAA |
| MYOG | CCAACCCAGGAGATCATTTG | ACGATGGACGTAAGGGAGTG |
| p21 | CCTGGTGATGTCCGACCTG | CCATGAGCGCATCGCAATC |
| p53 | CCCCTGTCATCTTTTGTCCCT | AGCTGGCAGAATAGCTTATTGAG |

**Table S3 Sequences of shRNA for ANXA2 and CB2R**

| Gene | Sequence |
| --- | --- |
| ANXA2-shNC (Shown as shNC in Fig.S4) | ACGUGACACGUUCGGAGAATT |
| ANXA2-shANXA2 (Shown as shANXA2 in Fig.S4) | UUCCUCUUGAAUUCAGAUCTT |
| CB2R-shNC (Shown as AAV-shNC in Fig.S8) | ACGUGACACGUUCGGAGAATT |
| CB2R-shCB2R (Shown as AAV-shCB2R in Fig. S8) | AAUCUCUCCACUCCGCAGGTT |

**Table S4 Sequences of siRNA for ANXA2 and Neu2**

| Gene | Sense Strand | Antisence Strand |
| --- | --- | --- |
| Anxa2 (m)-si1 | GAGUGUACAAGGAAAUGUATT | UACAUUUCCUUGUACACUCTT |
| Anxa2 (m)-si2 | GCAUCAAGAAAGAGGUCAATT | UUGACCUCUUUCUUGAUGCTT |
| Anxa2 (m)-si3 | GCAAGUCCCUGUACUACUATT | UAGUAGUACAGGGACUUGCTT |
| neu2 (m)-si1 | GAGUUGAUUGUCCUGAGAATT | UUCUCAGGACAAUCAACUCTT |
| neu2 (m)-si2 | CAAAGACCCUCUUCCUUUUTT | AAAAGGAAGAGGGUCUUUGTT |
| neu2 (m)-si3 | GUUUGGGUGUCUGUAUGAATT | UUCAUACAGACACCCAAACTT |
